# Single- and Multiple-Group Penalized Factor Analysis: A Trust-Region Algorithm Approach with Integrated Automatic Multiple Tuning Parameter Selection

**DOI:** 10.1007/s11336-021-09751-8

**Published:** 2021-03-26

**Authors:** Elena Geminiani, Giampiero Marra, Irini Moustaki

**Affiliations:** 1 Department of Statistical Sciences, University of Bologna, Via Delle Belle Arti 41, 40126 Bologna, Italy; 2 Department of Statistical Science, University College London, London, UK; 3 Department of Statistics, London School of Economics and Political Science, London, UK

**Keywords:** effective degrees of freedom, generalized information criterion, measurement invariance, penalized likelihood, simple structure

## Abstract

Penalized factor analysis is an efficient technique that produces a factor loading matrix with many zero elements thanks to the introduction of sparsity-inducing penalties within the estimation process. However, sparse solutions and stable model selection procedures are only possible if the employed penalty is non-differentiable, which poses certain theoretical and computational challenges. This article proposes a general penalized likelihood-based estimation approach for single- and multiple-group factor analysis models. The framework builds upon differentiable approximations of non-differentiable penalties, a theoretically founded definition of degrees of freedom, and an algorithm with integrated automatic multiple tuning parameter selection that exploits second-order analytical derivative information. The proposed approach is evaluated in two simulation studies and illustrated using a real data set. All the necessary routines are integrated into the R package penfa.

**Supplementary Information:**

The online version contains supplementary material available at 10.1007/s11336-021-09751-8.

## Introduction

Factor analysis has been extensively applied in the social, behavioral and natural sciences as a data reduction method. For a given set of observed variables $x_1, \ldots , x_p$ one would like to find a set of latent factors $f_1, \ldots , f_r$, fewer in number than the observed variables ($r < p$), that contain essentially the same information. Factor analysis can be conducted in an exploratory (EFA; Mulaik, 2009) or confirmatory (CFA; Jöreskog, 1979) way. EFA analyzes a set of correlated observed variables without knowing in advance either the number of factors that are required to explain their interrelationships or their meaning. Depending on the *r*-factor model finally chosen (based on goodness-of-fit criteria and fit measures) as well as the rotation applied, an interpretation and labelling of the factors are given. CFA postulates certain relationships among the observed and latent variables by assuming a pre-specified pattern for the model parameters (factor loadings, structural parameters, unique variances). It is mainly concerned with testing hypotheses about the values of the factor loadings (usually, that some of them are zero).

In data reduction techniques such as factor analysis, the interest is in obtaining factor solutions that exhibit a “simple structure” (Thurstone, 1947), that is, with many zero loadings and pure measures (i.e., each variable loads only on a single factor). In EFA this is accomplished with orthogonal or oblique factor rotations. However, rotations often do not generate loadings precisely equal to zero, so users have to manually set to zero those loadings that are smaller than a threshold (e.g., 0.30; Hair et al., 2010). Secondly, because each rotation is based on a specific optimization criterion, different rotations often lead to different factor structures which may all be far from “simple”. In CFA, one usually resorts to modification indices (Chou & Huh, 2012) instead, but, if used extensively, they can lead to higher risks of capitalization on chance (MacCallum et al., 1992), and a lower probability of finding the best model specification (Chou & Bentler, 1990).

Penalized factor analysis is an alternative technique that produces parsimonious models using largely an automated procedure. The resulting models are less prone to instability in the estimation process and are easier to interpret and generalize than their unpenalized counterparts. It is based on the use of penalty functions that allow a subset of the model parameters (typically the factor loadings) to be automatically set to zero. The penalty is usually non-differentiable (Fan & Li, 2001), so that it produces a sparse factor structure, that is, a loading matrix where the number of nonzero entries is much smaller than the total number of its elements. This definition does not impose any pattern on the nonzero entries, so a simple structure is not enforced if it is not supported by the data. These sparsity-inducing penalties can reduce model complexity, enhance the interpretability of the results, and produce more stable parameter estimates. These benefits come, however, with a loss in model fit (i.e., a nonzero bias), so it is crucial to balance goodness of fit and sparsity appropriately. This can be achieved via the selection of a tuning parameter, which controls the amount of sparsity introduced in the model. A grid-search over a range of tuning values is generally conducted, and the optimal model is picked on the basis of information criteria or cross-validation.

In the last few years, several works have applied penalized estimation and regularization methods to models with latent variables. Choi, Oehlert and Zou (2010) used lasso (“least absolute shrinkage and selection operator”; Tibshirani, 1996) and adaptive lasso penalties in EFA. Since the lasso leads to biased estimates and overly dense factor structures, Hirose and Yamamoto (2014a; b) employed non-convex penalties, such as the scad (“smoothly clipped absolute deviation”) and the mcp (“minimax concave penalty”). Trendafilov, Fontanella and Adachi (2017) penalized a reparametrized loading matrix, whereas Jin, Moustaki and Yang-Wallentin (2018) considered a quadratic approximation of the objective function. Regularized methods have also been applied to structural equation models (SEM) for which CFA is a special case. Jacobucci, Grimm and McArdle (2016) developed the regularized SEM (RegSEM) using a reticular action model formulation and coordinate descent or general optimization routines. Huang, Chen and Weng (2017) and Huang (2020) examined the same problem of penalizing a SEM but employed a modification of the quasi-Newton algorithm.

Penalized estimation can be also extended to multiple-group analyses, such as cross-national surveys or cross-cultural assessments in psychological or educational testing. Recently, Huang (2018) and Lindstrøm and Dahl (2020) developed a penalized approach for multiple-group SEM, showing the benefits of using regularization techniques as alternatives to factorial invariance testing procedures (Meredith, 1993) to ascertain the differences and similarities of the parameter estimates across groups (see Bauer, Belzak & Cole, 2020 for a regularized approach for moderated non-linear factor analysis).

In this paper, we propose a penalized-estimation strategy for single- and multiple-group factor analysis models based on a carefully structured trust-region algorithm. The penalized optimization problem requires the availability of second-order analytical derivative information and thus twice-continuously differentiable functions. Because a sparse solution can be only achieved with non-differentiable penalties, we employ differentiable approximations of them. In particular, we locally approximate several convex and non-convex penalties, including lasso, adaptive lasso, scad and mcp. We also provide a theoretically founded definition of degrees of freedom (required when performing model selection) and present an efficient automatic procedure for the estimation of the tuning parameters, hence eliminating the need for computationally intensive grid searches as done in the literature. The proposed methodology is integrated into the R package penfa (a short form for *PENalized Factor Analysis*).

The paper is organized as follows. Sect. 2 briefly discusses the classical linear factor analysis model. In Sect. 3 we review and develop penalized likelihood estimation via locally approximated penalties. The extension of the model and the penalized approach for the case of multiple groups are described in Sects. 4 and 5, respectively. The derivation of the model degrees of freedom is presented in Sect. 6. Parameter estimation and the automatic selection of the tuning parameters are detailed in Sect. 7. The performance of the model is evaluated in two simulation studies (Sect. 8) and an empirical application (Sect. 9). Lastly, Sect. 10 concludes the paper and gives directions for future research. Additional details can be found in the Online Resources.

## The Normal Linear Factor Analysis Model

The classical linear factor analysis model takes the form^1^:

1 $$\begin{aligned} {\varvec{x}} = {\varvec{\Lambda }} {\varvec{f}} + {\varvec{\varepsilon }}, \end{aligned}$$

where ${\varvec{x}}$ is the $p \times 1$ vector of observed variables, ${\varvec{\Lambda }}$ is the $p \times r$ factor loading matrix, ${\varvec{f}}$ is the $r \times 1$ vector of common factors, and ${\varvec{\varepsilon }}$ is the $p \times 1$ vector of unique factors. It is assumed that ${\varvec{f}} \sim {\mathcal {N}}({\varvec{0}}, {\varvec{\Phi }})$, ${\varvec{\varepsilon }} \sim {\mathcal {N}}({\varvec{0}}, {\varvec{\Psi }})$, and ${\varvec{f}}$ is independent of ${\varvec{\varepsilon }}$. The observed variables are assumed to be conditionally independent (i.e., ${\varvec{\Psi }}$ is a diagonal matrix), although this assumption can be relaxed if required. It then follows that ${\varvec{x}} \sim {\mathcal {N}}({\varvec{0}}, {\varvec{\Sigma }})$, where the model-implied covariance matrix is ${\varvec{\Sigma }} = {\varvec{\Lambda }} {\varvec{\Phi }} {\varvec{\Lambda }}^T + {\varvec{\Psi }}$.

It is possible to fix certain elements in ${\varvec{\Lambda }}, {\varvec{\Phi }}$ and ${\varvec{\Psi }}$ to zero based on a data generating hypothesis. The remaining $m \le \min \left( N, \frac{p(p+1)}{2}\right)$ elements, with *N* the total sample size, constitute the free parameters in $\text {vec}({\varvec{\Lambda }})$, $\text {diag}({\varvec{\Psi }})$, and $\text {vech}({\varvec{\Phi }})$, and are collected in the vector ${\varvec{\theta }}$, where the vec($\cdot$) operator converts the enclosed matrix into a vector by stacking its columns, $\text {diag}(\cdot )$ extracts the diagonal elements of the enclosed square matrix, and vech($\cdot$) vectorizes the lower-diagonal part of the enclosed symmetric matrix. As it is common practice in these cases, we assume that the observed variables are measured as deviations from their means, so that the parameters only strive to reproduce the covariance matrix. As in Jöreskog (1979), we fix the variances of the common factors to unity for scale setting, and $r-1$ elements of ${\varvec{\Lambda }}$, in each column, to zero for uniqueness under factor rotation.

For a random sample of size *N* the log-likelihood is written as

2 $$\begin{aligned} \ell ({\varvec{\theta }}) = - \frac{N}{2} \left\{ \log |{\varvec{\Sigma }} |+ \text {tr}( {\varvec{S}} {\varvec{\Sigma }}^{-1}) + p \log (2 \pi ) \right\} , \end{aligned}$$

where ${\varvec{S}}$ is the sample covariance matrix. Since we are interested in introducing sparsity in the factor loading matrix, the estimation of the factor model will involve penalized likelihood procedures. The next section illustrates how such sparsity-inducing penalty functions can be specified and suitably approximated.

## Sparsity-Inducing Penalties

Since the primary interest of factor analysis is a sparse loading matrix, penalization is imposed on the factor loading matrix ${\varvec{\Lambda }}$. Let us write the parameter vector as ${\varvec{\theta }} = (\theta _1, \ldots , \theta _{q^\star }, \theta _{q^\star +1}, \ldots , \theta _m)^T$, where the sub-vector $(\theta _1, \ldots , \theta _{q^\star })^T$ collects the penalized parameters (i.e., the factor loadings), whereas $(\theta _{q^\star +1}, \ldots , \theta _m)^T$ the unpenalized parameters (i.e., the free elements in ${\varvec{\Psi }}$ and ${\varvec{\Phi }}$). Because of the presence of fixed elements in ${\varvec{\Lambda }}$ (Sect. 2), the number of penalized factor loadings $q^\star$ is smaller than $p\times r$. Define ${\varvec{R}}_q = \text {diag}(0, 0, \ldots , 0, 1, 0, \ldots , 0)$ a diagonal matrix where the 1 on the $(q, q)^{\text {th}}$ entry of the matrix corresponds to the $q^{\text {th}}$ parameter in ${\varvec{\theta }}$, for $q=1, \ldots , q^\star$, and ${\varvec{R}}_q = {\varvec{O}}_{m \times m}$ for $q = q^\star +1 , \ldots , m$. Let ${\mathcal {P}}_\eta ({\varvec{\theta }})$ be a penalty function on the parameter vector ${\varvec{\theta }}$, where $\eta \in [0, \infty )$ is a positive tuning parameter which determines the amount of shrinkage or penalization. The overall penalty is then given by the sum of the penalty terms for each parameter, that is, ${\mathcal {P}}_\eta ({\varvec{\theta }}) = \sum _{q=1}^{m} {\mathcal {P}}_{\eta ,q} (||{\varvec{R}}_q {\varvec{\theta }}||_1)$, where $||{\varvec{R}}_q {\varvec{\theta }}||_1 = |\theta _q |$ if $q=1, \ldots , q^\star$, and zero otherwise. An example clarifying the formulation of this penalty is provided in Section B.1.1. One of the best-known penalties is the lasso (Tibshirani, 1996), which is defined as

3 $$\begin{aligned} {\mathcal {P}}^L_\eta ({\varvec{\theta }}) = \eta \sum _{q=1}^{q^\star } |\theta _{q} |. \end{aligned}$$

The potential problem with this penalty is that it penalizes all parameters equally, and thus can either select an overly complicated model or over-shrink large parameters. An ideal penalty should induce weak shrinkage on large effects and strong shrinkage on irrelevant effects (Tang, Shen, Zhang & Yi, 2017). To address this issue, alternative penalties have been developed, the most common ones being the adaptive lasso (alasso; Zou, 2006), scad (Fan & Li, 2001) and mcp (Zhang, 2010). These penalties give different amounts of shrinkage to each parameter, so each factor loading is weighted differently. Because of this, they lead to sparser solutions and enjoy the so-called “oracle” property, that is, when the true parameters have some zero loadings, they are estimated as zero with probability tending to one, and the nonzero loadings are estimated as well as when the correct submodel is known (Fan & Li, 2001). The alasso is defined as

4 $$\begin{aligned} {\mathcal {P}}^A_\eta ({\varvec{\theta }}) = \eta \sum _{q=1}^{q^\star } w_q |\theta _q |= \eta \sum _{q=1}^{q^\star } \frac{|\theta _q |}{ |{\hat{\theta }}_q |^{a}} \quad \text { for } a > 0. \end{aligned}$$

It uses an adaptive weighting scheme based on a set of available weights $w_q = \dfrac{1}{|{\hat{\theta }} _q |^{a}} \, (q = 1, \ldots , q^\star )$, which are often taken to be the maximum likelihood estimates, that is, $w_q = \dfrac{1}{|{\hat{\theta }}_q^{\text {MLE}}|^{a}}$. As the exponent *a* gets larger, the relative strength of the penalization increases for smaller maximum likelihood estimates compared to larger maximum likelihood estimates.

Similarly, the scad and mcp use a varying weighting scheme. The scad is defined as5and the mcp as6where *a* is an additional tuning parameter. The superscripts *L*, *A*, *S*, *M* in equations (3)-(6) refer to the lasso, alasso, scad and mcp, respectively. The derivations of expressions (3)-(6) can be found in Section B.1.2. While the lasso and alasso are convex penalties, the scad and mcp are non-convex and can, therefore, make the optimization problem non-convex. In fact, a challenge with non-convex penalties is to find a good balance between sparsity and stability. To this end, both scad and mcp have an extra tuning parameter (*a*) which regulates their concavity so that, when it exceeds a threshold, the optimization problem becomes convex.

The above penalties help to obtain sparse solutions, however, they are non-differentiable, which is problematic for developing a coherent computational and theoretical inferential framework. The next section addresses this issue by replacing the non-differentiable penalties with their differentiable counterparts obtained via local approximations.

### Locally Approximated Penalties

Ulbricht (2010) pointed out that a good penalty function should satisfy the following properties, for $q = 1, \ldots , m$: (P.1) ${\mathcal {P}}_{\eta ,q}: {\mathbb {R}}^+ \rightarrow {\mathbb {R}}^+$ and ${\mathcal {P}}_{\eta ,q}(0) = 0$; (P.2) ${\mathcal {P}}_{\eta ,q}(||{\varvec{R}}_q {\varvec{\theta }}||_1)$ continuous and strictly monotone in $||{\varvec{R}}_q {\varvec{\theta }}||_1$; (P.3) ${\mathcal {P}}_{\eta ,q}(||{\varvec{R}}_q {\varvec{\theta }}||_1)$ continuously differentiable $\forall \, ||{\varvec{R}}_q {\varvec{\theta }}||_1 \ne 0$, such that $\dfrac{\partial {\mathcal {P}}_{\eta ,q} (||{\varvec{R}}_q {\varvec{\theta }}||_1)}{\partial ||{\varvec{R}}_q {\varvec{\theta }}||_1} > 0$. We develop differentiable approximations of the above penalties that satisfy these properties. These approximations make the objective function differentiable, which is an indispensable prerequisite for the theoretical derivation of the degrees of freedom of the model, and a computationally and theoretically founded estimation framework (Sects. 6–7). In the same spirit, as for instance, Filippou, Marra and Radice (2017), we locally approximate the non-differentiable $L_1$-norms in (3)-(6) at $||{\varvec{R}}_q {\varvec{\theta }}||_1 = 0$ and combine this with ideas by Fan and Li (2001) and Ulbricht (2010). Let $||{\varvec{R}}_q {\varvec{\theta }}||_1 = ||{\varvec{\xi }}_q||_1$, where the $q^{\text {th}}$ element in ${\varvec{\xi }}_q = (0, \ldots , 0, \theta _q, 0, \ldots , 0)^T$ corresponds to the $q^{\text {th}}$ parameter in ${\varvec{\theta }}$. Assume that an approximation ${\mathcal {K}}_1({\varvec{\xi }}_q, {\mathcal {A}})$ of the $L_1$-norm $||\cdot ||_1$ exists such that

$$\begin{aligned} ||{\varvec{\xi }}_q||_1 = {\mathcal {K}}_1({\varvec{\xi }}_q, {\mathcal {B}}) = \lim _{{\mathcal {A}} \rightarrow {\mathcal {B}}} {\mathcal {K}}_1({\varvec{\xi }}_q, {\mathcal {A}}), \end{aligned}$$

where ${\mathcal {A}}$ represents a set of possible tuning parameters, ${\mathcal {B}}$ is the set of boundary values for $||{\varvec{\xi }}_q||_1$ and ${\mathcal {K}}_1({\varvec{\xi }}_q, {\mathcal {A}})$ is at least twice differentiable. We use $||{\varvec{\xi }}_q||_1 = {\mathcal {K}}_1({\varvec{\xi }}_q, {\mathcal {A}}) = ({\varvec{\xi }}_q^T {\varvec{\xi }}_q + \bar{c})^{\frac{1}{2}}$ (Koch, 1996), with $\bar{c}$ a small positive real number (e.g., $10^{-8}$) which controls the closeness between the approximation and the exact function. For all ${\varvec{\xi }}_q$ for which the derivative $\dfrac{\partial ||{\varvec{\xi }}_q||_1}{\partial {\varvec{\xi }}_q}$ is defined, we assume that

$$\begin{aligned} \frac{\partial ||{\varvec{\xi }}_q||_1}{\partial {\varvec{\xi }}_q} = \frac{\partial {\mathcal {K}}_1({\varvec{\xi }}_q, {\mathcal {B}})}{\partial {\varvec{\xi }}_q} = \lim _{{\mathcal {A}} \rightarrow {\mathcal {B}}} {\mathcal {D}}_1({\varvec{\xi }}_q, {\mathcal {A}}), \end{aligned}$$

where ${\mathcal {D}}_1({\varvec{\xi }}_q, {\mathcal {A}}) = \dfrac{\partial {\mathcal {K}}_1({\varvec{\xi }}_q, {\mathcal {A}})}{\partial {\varvec{\xi }}_q}$, and that ${\mathcal {D}}_1 ({\varvec{0}}, {\mathcal {A}}) = {\varvec{0}}$. Then, the first derivative ${\mathcal {D}}_1({\varvec{\xi }}_q, {\mathcal {A}}) = ({\varvec{\xi }}_q^T {\varvec{\xi }}_q + \bar{c})^{-\frac{1}{2}} {\varvec{\xi }}_q$ is a continuous approximation of the first-order derivative of the $L_1$-norm. Notice that ${\mathcal {K}}_1({\varvec{\xi }}_q, {\mathcal {A}})$ deviates only slightly from ${\mathcal {K}}_1({\varvec{\xi }}_q, {\mathcal {B}})$: when ${\varvec{\xi }}_q = {\varvec{0}}$ the deviation is $\sqrt{\bar{c}}$, whereas for any other value of ${\varvec{\xi }}_q$ the deviation is less than $\bar{c}$.

Penalty ${\mathcal {P}}^{{\mathcal {T}}}_\eta ({\varvec{\theta }})$ for ${\mathcal {T}} = \{L, A, S, M\}$ can be locally approximated by a quadratic function as follows. Suppose that ${\tilde{\varvec{\theta }}}$ is an initial value close to the true value of ${\varvec{\theta }}$. Then, we approximate ${\mathcal {P}}^{{\mathcal {T}}}_\eta ({\varvec{\theta }})$ by a Taylor expansion of order one at ${\tilde{\varvec{\theta }}}$, that is,

7 $$\begin{aligned} {\mathcal {P}}^{{\mathcal {T}}}_\eta ({\varvec{\theta }}) \approx {\mathcal {P}}^{{\mathcal {T}}}_\eta ({\tilde{\varvec{\theta }}}) + \nabla _{{\tilde{\varvec{\theta }}}} {\mathcal {P}}^{{\mathcal {T}}}_\eta ({\tilde{\varvec{\theta }}})^T ({\varvec{\theta }} - {\tilde{\varvec{\theta }}}), \end{aligned}$$

where $\nabla _{{\tilde{\varvec{\theta }}}} {\mathcal {P}}^{{\mathcal {T}}}_\eta ({\tilde{\varvec{\theta }}}) = \dfrac{\partial {\mathcal {P}}^{{\mathcal {T}}}_\eta ({\tilde{\varvec{\theta }}})}{\partial {\tilde{\varvec{\theta }}}}$. As proved in Section B.1.3, ${\mathcal {P}}^{{\mathcal {T}}}_\eta ({\varvec{\theta }})$ is approximated asThe penalty matrix  is an $m \times m$ block diagonal matrix of the form:8The first block is composed of the $q^\star \times q^\star$ diagonal matrix  and corresponds to the parameters to penalize, whereas the second block is an $(m-q^\star )$-dimensional null matrix relative to the parameters unaffected by the penalization. The matrix  is in turn a diagonal matrix whose entries $m_q^{{\mathcal {T}}} = \dfrac{\partial {\mathcal {P}}^{{\mathcal {T}}}_{\eta ,q} (||{\varvec{R}}_q {\tilde{\varvec{\theta }}}||_1)}{\partial ||{\varvec{R}}_q {\tilde{\varvec{\theta }}}||_1}\dfrac{1}{\sqrt{({\varvec{R}}_q {\tilde{\varvec{\theta }}})^T {\varvec{R}}_q {\tilde{\varvec{\theta }}} + \bar{c}}}$ (for $q = 1, \ldots , q^\star$) determine the amount of shrinkage on ${\tilde{\theta }}_q$ controlled by the tuning $\eta$ and required by penalty ${\mathcal {T}}$. Their expressions for the lasso, alasso, scad and mcp are (see Section B.1.3.1)9101112

## The Multiple-Group Factor Analysis Model

In studies of multiple groups of respondents, such as cross-national surveys and cross-cultural assessments in psychological or educational testing, the interest often lies in the comparisons of the groups with respect to their factor structures. In this case, the model becomes

13 $$\begin{aligned} {\varvec{x}}_g = {\varvec{\tau }}_g + {\varvec{\Lambda }}_g {\varvec{f}}_g + {\varvec{\varepsilon }}_g \qquad \text {for } g = 1, \ldots , G, \end{aligned}$$

where the subscript *g* denotes the group, and ${\varvec{\tau }}_g$ the intercept terms. It is assumed that ${\varvec{f}}_g \sim {\mathcal {N}}({\varvec{\kappa }}_g, {\varvec{\Phi }}_g)$, ${\varvec{\varepsilon }}_g \sim {\mathcal {N}}({\varvec{0}}, {\varvec{\Psi }}_g)$, ${\varvec{f}}_g$ is independent of ${\varvec{\varepsilon }}_g$, and ${\varvec{\Psi }}_g$ is a diagonal matrix. Then, it follows that ${\varvec{x}}_g \sim {\mathcal {N}}({\varvec{\mu }}_g, {\varvec{\Sigma }}_g)$, where the model-implied moments are ${\varvec{\mu }}_g = {\varvec{\tau }}_g + {\varvec{\Lambda }}_g {\varvec{\kappa }}_g$ and ${\varvec{\Sigma }}_g = {\varvec{\Lambda }}_g {\varvec{\Phi }}_g {\varvec{\Lambda }}_g^T + {\varvec{\Psi }}_g$.

We set the metric of the factors and the necessary identification restrictions through the “marker-variable” approach (Little, Slegers & Card, 2006), which relies on the selection of a representative variable (marker) for each factor in each group. Then, we fix the intercepts of the markers to zero, the loadings on the “marked” factors to 1.0, and those on the remaining factors to zero. All of the other parameters are estimated. The choice of the markers is crucial and should be an accurate one (Millsap, 2001). Alternative identification methods are discussed in Millsap (2012).

The free parameters of each group appearing in $\text {vec}({\varvec{\Lambda }}_g)$, ${\varvec{\tau }}_g$, $\text {diag}({\varvec{\Psi }}_g)$, $\text {vech}({\varvec{\Phi }}_g)$, and ${\varvec{\kappa }}_g$ are collected in the $m_g$-dimensional vector ${\varvec{\theta }}_g$, for $g=1, \ldots , G$. Each group parameter vector is collected in the overall *m*-dimensional vector ${\varvec{\theta }} = ({\varvec{\theta }}_1^T, \ldots , {\varvec{\theta }}_g^T, \ldots , {\varvec{\theta }}_G^T)^T$, where $m = \sum _{g=1}^{G} m_g$. Assume for convenience that the same set of parameters is estimated in every group, which implies that the number of observed variables *p* and factors *r* is the same across groups, the fixed elements required for identification are placed in the same positions across groups, and that $m_1 = \ldots = m_G$, so that $m = m_1 G$. Given random samples of sizes $N_1, \ldots , N_G$, with $N = \sum _{g=1}^{G} N_g$ the total sample size across groups, the log-likelihood of the multiple-group factor model is

14 $$\begin{aligned} \ell ({\varvec{\theta }}) = - \sum _{g=1}^{G} \frac{N_g}{2} \{ \log |{\varvec{\Sigma }}_g |+ \text {tr}({\varvec{W}}_g {\varvec{\Sigma }}_g^{-1}) + p \log (2 \pi ) \}, \end{aligned}$$

where $${\varvec{W}}_g = {\varvec{S}}_g + (\bar{{\varvec{x}}}_g - {\varvec{\mu }}_g)(\bar{{\varvec{x}}}_g - {\varvec{\mu }}_g)^T$$.

In multiple-group analyses, an important methodological consideration is the establishment of the comparability or “equivalence” of measurement across the groups (e.g., countries, socio-economical groups). Measurement (or factorial) invariance occurs when the factors have the same meaning in each group, which translates into equal measurement models (i.e., factor loadings, intercepts and unique variances) across groups (Millsap 2012). If non-equivalence of measurement exists, substantively interesting group comparisons may become distorted. Testing for measurement invariance in the parameters is, however, an intensive process. A sequence of nested tests is progressively conducted to establish the equivalence in the factor loadings, the intercepts, and optionally the unique variances (Vandenberg & Lance, 2000). The next section describes the penalty functions that can be incorporated into the multiple-group model to obtain a technique that automatically detects parameter equivalence across groups.

## Sparsity and Invariance-Inducing Penalties

As in the single-group factor model, we can penalize the factor loadings to automatically obtain a sparse loading matrix in each of the groups. Define the diagonal matrix ${\varvec{R}}_q = \text {diag}(0, \ldots , 0, 1, 0,$ $\ldots , 0)$, where the 1 on the $(q,q)^{\text {th}}$ entry of the matrix corresponds to the $q^{\text {th}}$ factor loading in ${\varvec{\theta }}$, for $q = (g-1)m_1 + 1, \ldots , (g-1)m_1 + q^\star$ and $g=1, \ldots , G$, and ${\varvec{R}}_q = {\varvec{O}}_{m \times m}$ for the remaining parameters. The quantity $q^\star$ represents the number of penalized loadings in each group. Then, the sparsity-inducing penalty on the factor loadings is ${\mathcal {P}}^{{\mathcal {T}}}_{\eta _1}({\varvec{\theta }}) = \sum _{q=1}^{m} {\mathcal {P}}^{{\mathcal {T}}}_{\eta _1,q}(||{\varvec{R}}_q {\varvec{\theta }}||_1)$, where $\eta _1 \in [0, \infty )$ controls the overall amount of shrinkage.

In the same spirit as factorial invariance, we can specify a penalty encouraging the equality of the loadings across groups. Conveniently, this can be achieved by shrinking the pairwise absolute differences of every factor loading across groups. Let ${\varvec{D}}_q^{{\varvec{\Lambda }}}$, for $q = 1, \ldots , q^\star$, be the matrix computing the differences of the factor loading pairs $(\theta _{(g-1)m_1 +q}, \theta _{(g'-1)m_1+q})$ for $g < g'$, whereas for the other parameters $${\varvec{D}}_q^{{\varvec{\Lambda }}} = {\varvec{O}}_{m_1 \left( {\begin{array}{c}G\\ 2\end{array}}\right) \times m}$$. Then, the penalty inducing equal loadings across groups can be written as ${\mathcal {P}}^{{\mathcal {T}}}_{\eta _2} ({\varvec{\theta }}) = \sum _{q=1}^{m} {\mathcal {P}}^{{\mathcal {T}}}_{\eta _2,q}(||{\varvec{D}}_q^{{\varvec{\Lambda }}} {\varvec{\theta }}||_1)$, where $||{\varvec{D}}_q^{{\varvec{\Lambda }}} {\varvec{\theta }}||_1 = \sum _{g < g'} |\theta _{(g-1)m_1 + q} - \theta _{(g'-1)m_1 + q} |$ for $q = 1, \ldots , q^\star$, and zero otherwise. If $G = 2$, the absolute difference of the $q^{\text {th}}$ loading across the two groups is expressed as $||{\varvec{D}}_q^{{\varvec{\Lambda }}} {\varvec{\theta }}||_1 = |\theta _q - \theta _{m_1 + q} |$, where ${\varvec{D}}_q^{{\varvec{\Lambda }}} = [{\varvec{R}}_q, \, \, -{\varvec{R}}_q]$. The expression of ${\mathcal {P}}^{{\mathcal {T}}}_{\eta _2} ({\varvec{\theta }})$ for lasso, alasso, scad and mcp is given in Section B.2.1. The tuning parameter $\eta _2 \in [0, \infty )$ controls the amount of loading equality across groups. When the loadings are truly invariant and $\eta _2$ is properly chosen, the penalized group loading matrices “fuse”, and share the same values.

Lastly, we can encourage the equality of the intercepts across groups by specifying a penalty shrinking their pairwise absolute group differences. Let $k^\star$ be the number of estimated intercepts in each group. Due to the presence of fixed elements in ${\varvec{\tau }}_g$ for identification, $k^\star$ is smaller than *p*. Let ${\varvec{D}}_q^{{\varvec{\tau }}}$, for $q=(g-1)m_1 + q^\star + 1, \ldots , (g-1)m_1 + q^\star + k^\star$, be a matrix of known constants computing the differences of the intercepts across groups, whereas for all of the other parameters (i.e., the loadings, the unique variances and the structural parameters) $${\varvec{D}}_q^{{\varvec{\tau }}} = {\varvec{O}}_{m_1 \left( {\begin{array}{c}G\\ 2\end{array}}\right) \times m}$$. The penalty inducing equal intercepts across groups is then written as ${\mathcal {P}}^{{\mathcal {T}}}_{\eta _3}({\varvec{\theta }}) = \sum _{q=1}^{m} {\mathcal {P}}^{{\mathcal {T}}}_{\eta _3,q}(||{\varvec{D}}_q^{{\varvec{\tau }}} {\varvec{\theta }}||_1)$, where $\eta _3 \in [0, \infty )$ governs the amount of intercept invariance.

Optionally, one can encourage the invariance of the unique variances. However, as argued by Little et al. (2012), these quantities contain both random sources of errors, for which there is no theoretical reason to expect equality across groups, and item-specific components, which can vary as a function of various measurement factors. In light of this, we do not introduce a penalty on the unique variances, as their cross-group equivalence would not provide any additional evidence of comparability of the constructs because the important measurement parameters (i.e., the factor loadings and the intercepts) are already encouraged to be invariant by penalties ${\mathcal {P}}^{{\mathcal {T}}}_{\eta _2}$ and ${\mathcal {P}}^{{\mathcal {T}}}_{\eta _3}$.

The three penalties can be easily combined into a single penalty that simultaneously generates sparsity on the factor loading matrices and equivalent loadings and intercepts

15 $$\begin{aligned} {\mathcal {P}}^{{\mathcal {T}}}_{{\varvec{\eta }}}({\varvec{\theta }})= & {} {\mathcal {P}}^{{\mathcal {T}}}_{\eta _1}({\varvec{\theta }}) + {\mathcal {P}}^{{\mathcal {T}}}_{\eta _2} ({\varvec{\theta }}) + {\mathcal {P}}_{\eta _3}^{{\mathcal {T}}} ({\varvec{\theta }}) \nonumber \\= & {} \sum _{q=1}^{m} \left\{ {\mathcal {P}}^{{\mathcal {T}}}_{\eta _1,q}(||{\varvec{R}}_q {\varvec{\theta }}||_1) + {\mathcal {P}}^{{\mathcal {T}}}_{\eta _2,q}(||{\varvec{D}}_q^{{\varvec{\Lambda }}} {\varvec{\theta }}||_1) + {\mathcal {P}}^{{\mathcal {T}}}_{\eta _3,q}(||{\varvec{D}}_q^{{\varvec{\tau }}} {\varvec{\theta }}||_1) \right\} , \end{aligned}$$

where ${\varvec{\eta }} = (\eta _1, \eta _2, \eta _3)^T$ is the vector of the tuning parameters. Each penalty is controlled by its own tuning parameter, as we do not a priori expect these values to be equal. The penalties in (15) can be any of the functions illustrated in Sect. 3, including lasso, alasso, scad and mcp, and different penalty functions can be in principle combined. Suppose that the adaptive weights are available for the intercepts but not for the full loading matrices, possibly due to some inadmissible loading values. In this case, one can combine the alasso penalty for intercept similarity with the lasso (which also supports the automatic procedure, contrarily to the scad and mcp) for sparsity and loading equivalence. By following the rationale described in Sect. 3.1, we replace each non-differentiable penalty in (15) with its differentiable local approximation:which leads to the following differentiable form of the combined penalty:16Additional details on the structure of the matrix  are given in Section B.2.1. For an example clarifying the formulation of these matrices for the multiple-group model, the reader is referred to Section B.2.2.

## Generalized Information Criterion

The previously illustrated penalties can be directly introduced within the estimation process by means of penalized maximum likelihood estimation procedures. The penalized log-likelihood is

17 $$\begin{aligned} \ell _p({\varvec{\theta }}) :=\sum _{\alpha = 1}^{N} \bigg \{ \ell ({\varvec{x}}_\alpha | {\varvec{\theta }}) - {\mathcal {P}}^{{\mathcal {T}}}_{{\varvec{\eta }}} ({\varvec{\theta }}) \bigg \} = \ell ({\varvec{\theta }}) - N \, {\mathcal {P}}^{{\mathcal {T}}}_{{\varvec{\eta }}} ({\varvec{\theta }}). \end{aligned}$$

For the normal linear factor model, $\ell ({\varvec{\theta }})$ is given in equation (2), ${\mathcal {P}}^{{\mathcal {T}}}_{{\varvec{\eta }}} ({\varvec{\theta }})$ is one of the penalties of Sect. 3 generating a sparse factor solution, and the vector ${\varvec{\eta }}$ reduces to the scalar $\eta$; for the multiple-group model, $\ell ({\varvec{\theta }})$ is given in equation (14), ${\mathcal {P}}^{{\mathcal {T}}}_{{\varvec{\eta }}} ({\varvec{\theta }})$ is one of the penalties of Sect. 5 inducing sparsity and invariant loadings and intercepts, and ${\varvec{\eta }}$ is equal to the triplet $(\eta _1, \eta _2, \eta _3)^T$.

Simultaneous estimation of all parameters is achieved by maximizing the penalized log-likelihood in (17) and using a local approximation of ${\mathcal {P}}^{{\mathcal {T}}}_{{\varvec{\eta }}} ({\varvec{\theta }})$, that is,18where the function in brackets is now twice-continuously differentiable. The penalized maximum likelihood estimator (PMLE) is then defined as $\hat{{\varvec{\theta }}} = \arg \max _{{\varvec{\theta }}} \ell _p ({\varvec{\theta }})$. Conveniently, the gradient of the penalized log-likelihood can be written as , where ${\varvec{g}}({\varvec{\theta }}) = \dfrac{\partial \ell ({\varvec{\theta }})}{\partial {\varvec{\theta }}}$, the Hessian matrix of the second-order derivatives , where , and the expected Fisher information , where .

A crucial aspect lies in the selection of ${\varvec{\eta }}$, which controls the amount of penalization introduced in the model. To select ${\varvec{\eta }}$, we elect to use the Generalized Information Criterion (GIC; Konishi & Kitagawa, 1996), which is based on a theoretically founded definition of degrees of freedom. Notice that this choice is possible because the quantities we are dealing with are twice-continuously differentiable. We resort to the general approach of the GIC because the penalized maximum likelihood estimator cannot be ascribed to the ordinary maximum likelihood framework postulated by the AIC, and not for relaxing the assumption ${\mathbb {E}}\left[ -\dfrac{\partial ^2 \ell ({\varvec{\theta }})}{\partial {\varvec{\theta }} \partial {\varvec{\theta }}^T}\right] = {\mathbb {E}}\left[ \dfrac{\partial \ell ({\varvec{\theta }})}{\partial {\varvec{\theta }}} \dfrac{\partial \ell ({\varvec{\theta }})}{\partial {\varvec{\theta }}^T}\right]$, which does hold true for the normal linear factor models considered in this paper. Let *G* be the true distribution function that generated the data $\pmb {{\mathscr {x}}}_N = \{{\varvec{x}}_1, \ldots , {\varvec{x}}_N\}$, which are realizations of the random vector . Let us express the parameter vector as ${\varvec{\theta }} = {\varvec{T}}(G)$, where ${\varvec{T}}(G)$ is the *m*-dimensional functional vector of *G* defined as the solution of the implicit equations $\int {\varvec{\psi }}({\varvec{x}}, {\varvec{T}}(G)) d G({\varvec{x}}) = {\varvec{0}}$, with . The log-likelihood and the penalty matrix take different forms depending on whether we deal with a single- or multiple-group factor model. The GIC evaluating the goodness of fit of the penalized model, when used to predict independent future data ${\varvec{z}}$ generated from the unknown distribution *G*, is (see Online Resource C)whereand ${\varvec{\eta }}$ enters the penalty matrix . By considering the PMLE $\hat{{\varvec{\theta }}}$ for ${\varvec{\theta }}$, and replacing the unknown distribution *G* with its empirical counterpart ${\hat{G}}$ based on the data, we havewhereThe effective number or estimated degrees of freedom (edf) of the model is thus equal to . The formula for the edf is thus readily obtained by adapting the existing results for general likelihoods (of which the differentiable function in (18) is an example) to the penalized framework and assuming the usual regularity conditions. The GIC is an extension of the Akaike Information Criterion (AIC; Akaike, 1974), and as such, it may inherit the tendency of the latter to select overly complex models. To avoid this issue, we can change the constant 2 of the bias term to $\log (N)$ (used in the Bayesian Information Criterion; Schwarz, 1978). Then, given grid(s) of values, the optimal $\hat{{\varvec{\eta }}}$ can be chosen using the following Generalized Bayesian Information Criterion (GBIC)19The optimal penalized factor model is hence chosen to be the one with the lowest BIC, as this is the information criterion routinely employed in sparse settings. However, if researchers are more interested in accuracy and achieving minimum prediction error, then the AIC is to be preferred. In the presence of moderate sample size and many variables, the extended BIC (EBIC; Chen & Chen, 2008) may be more suitable.

The edf of an unpenalized model () coincide with the dimension of the parameter vector $\varvec{\theta }$, since , where ${\varvec{I}}_m$ is the $m \times m$ identity matrix. For a penalized model . This shows that $\hbox {edf} \rightarrow m$ as ${\varvec{\eta }} \rightarrow {\varvec{0}}$, and $\hbox {edf} \rightarrow m - r^\star$ as ${\varvec{\eta }} \rightarrow {\varvec{\infty }}$, where $r^\star$ is the number of penalized elements (equal to $q^\star$ for the factor model, and $G(q^\star + k^\star )$ for the multiple-group extension). When ${\varvec{0}}< {\varvec{\eta }} < {\varvec{\infty }}$, the $\hbox {edf} \in [m - r^\star , m]$. The overall edf of a fitted model is given by the sum of the edf for each parameter; each single edf takes a value in the range [0, 1] and quantifies precisely the extent to which each coefficient is penalized.

The existing penalized factor models (Choi et al., 2010; Hirose & Yamamoto, 2014a; Jacobucci et al., 2016; Huang et al., 2017; Huang, 2018; Jin et al., 2018) compute the degrees of freedom as the number of nonzero parameters (referred in the following as dof), by advocating the fact that the number of nonzero coefficients in a lasso-penalized linear model gives an unbiased estimate of the total degrees of freedom (Zou et al., 2007). This way of estimating the degrees of freedom implies that each dof can be either 0 if its parameter has been shrunken to zero, or 1 otherwise. On the contrary, the edf can take any value in [0, 1]. This suggests that, while the definitions of dof and edf may produce equivalent results (for penalties enjoying the oracle property, as the alasso, scad and mcp), in practical situations using edf is expected to yield better-calibrated degrees of freedom. The proposed method also treats the estimated edf as they are. Importantly, the definition of edf directly stems from the estimated bias term of the GIC, which gives it a theoretically founded basis.

## Penalized Maximum Likelihood Estimation

For any given set of values of ${\varvec{\eta }}$ in the penalty matrix, which is hence denoted in the following as , we minimize $- \ell _p({\varvec{\theta }})$ via a trust-region algorithm (Nocedal & Wright, 2006). At iteration ${\mathfrak {t}}$, a “model function” ${\mathcal {Q}}_p^{[{\mathfrak {t}}]}$ is constructed, whose behavior near the current point ${\varvec{\theta }}^{[{\mathfrak {t}}]}$ is similar to that of the actual objective function. The model function is usually a quadratic approximation of $-\ell _p$ at ${\varvec{\theta }}^{[{\mathfrak {t}}]}$:where ${\varvec{s}}$ is the trial step vector aiming at reducing the model function, ${\varvec{g}}_p({\varvec{\theta }}^{[{\mathfrak {t}}]})$ the penalized score function, and  the penalized Hessian matrix. For the normal linear factor model, the derivation of the second-order derivatives is a tedious and lengthy process; however, the availability of these quantities guarantees a better accuracy of the algorithm since no numerical approximation is employed. Because the Hessian requires computing many elements, we also considered the Fisher information matrix. If the elements of $(\hat{{\varvec{\Sigma }}} - {\varvec{S}})$ are small and the second derivatives not too large, which is often the case, the information matrix is very close to the true Hessian. For the multiple-group model, due to the presence of the parameters for the mean structure besides those for the covariance structure, we only considered the information matrix as it exhibited similar numerical performances to the Hessian at a reduced computational cost. The analytical expressions of these derivatives for the single- and multiple-group model are given in Geminiani (2020, Appendices A, F, respectively).

The search for a minimizer of ${\mathcal {Q}}_p^{[{\mathfrak {t}}]}$ is restricted to some region around ${\varvec{\theta }}^{[{\mathfrak {t}}]}$, which is usually the ball $||{\varvec{s}}||_2 < \Delta ^{[{\mathfrak {t}}]}$, where $||\cdot ||_2$ is the Euclidean norm, and $\Delta ^{[{\mathfrak {t}}]} > 0$ the trust-region radius at iteration ${\mathfrak {t}}$. The size of the trust region is critical to the effectiveness of each step: if it is too small, the algorithm may miss the opportunity to take a step that moves it closer to the minimizer of the objective function; if it is too large, the minimizer of the model may be far from the one of the objective function in the region, so it may be necessary to reduce the region size and repeat the process. Each iteration of the trust-region algorithm solves the subproblem:

20 $$\begin{aligned} {\varvec{s}}^{[{\mathfrak {t}}]}= & {} \arg \min _{ {\varvec{s}} \in {\mathbb {R}}^m} {\mathcal {Q}}_p^{[{\mathfrak {t}}]}({\varvec{s}}) \qquad \text {subject to } ||{\varvec{s}}||_2 \le \Delta ^{[{\mathfrak {t}}]}, \end{aligned}$$

21 $$\begin{aligned} {\varvec{\theta }}^{[{\mathfrak {t}}+1]}= & {} {\varvec{\theta }}^{[{\mathfrak {t}}]} + {\varvec{s}}^{[{\mathfrak {t}}]}, \end{aligned}$$

where the current iteration ${\varvec{\theta }}^{[{\mathfrak {t}}]}$ is updated with ${\varvec{s}}^{[{\mathfrak {t}}]}$ if this step produces an improvement over the objective function. The size of the region is chosen by measuring the agreement between the model function and the objective function at previous iterations through the ratio:

22 $$\begin{aligned} r^{[{\mathfrak {t}}]} = \dfrac{- \left[ \ell _p({\varvec{\theta }}^{[{\mathfrak {t}}]}) - \ell _p({\varvec{\theta }}^{[{\mathfrak {t}}]} + {\varvec{s}}^{[{\mathfrak {t}}]}) \right] }{{\mathcal {Q}}_p^{[{\mathfrak {t}}]}({\varvec{0}}) - {\mathcal {Q}}_p^{[{\mathfrak {t}}]}({\varvec{s}}^{[{\mathfrak {t}}]})}. \end{aligned}$$

The numerator quantifies the actual reduction, and the denominator the predicted reduction. If $r^{[{\mathfrak {t}}]}$ is negative, the model is an inadequate representation of the objective function over the current trust region, so the step ${\varvec{s}}^{[{\mathfrak {t}}]}$ is rejected, and the new problem is solved with a smaller region. If $r^{[{\mathfrak {t}}]}$ is close to 1, there is good agreement between ${\mathcal {Q}}_p^{[{\mathfrak {t}}]}$ and $-\ell _p$ over ${\varvec{s}}^{[{\mathfrak {t}}]}$, so the model can accurately predict the behavior of the objective function along that step, and the trust region is enlarged for the next iteration. If $r^{[{\mathfrak {t}}]}$ is positive, but not close to 1, the trust region is not altered, unless it is close to zero or negative, in which case it is shrunken. Algorithm 1 describes the process. The term $\Delta _{\text {max}}$ represents an overall bound on the step lengths. Trust-region algorithms never run too far from the current iteration as the points outside the trust region are not considered. For this reason, they were shown to be more stable and faster than line search methods, particularly for functions that are non-concave and/or exhibit regions close to flat (Radice, Marra & Wojtyś, 2016).
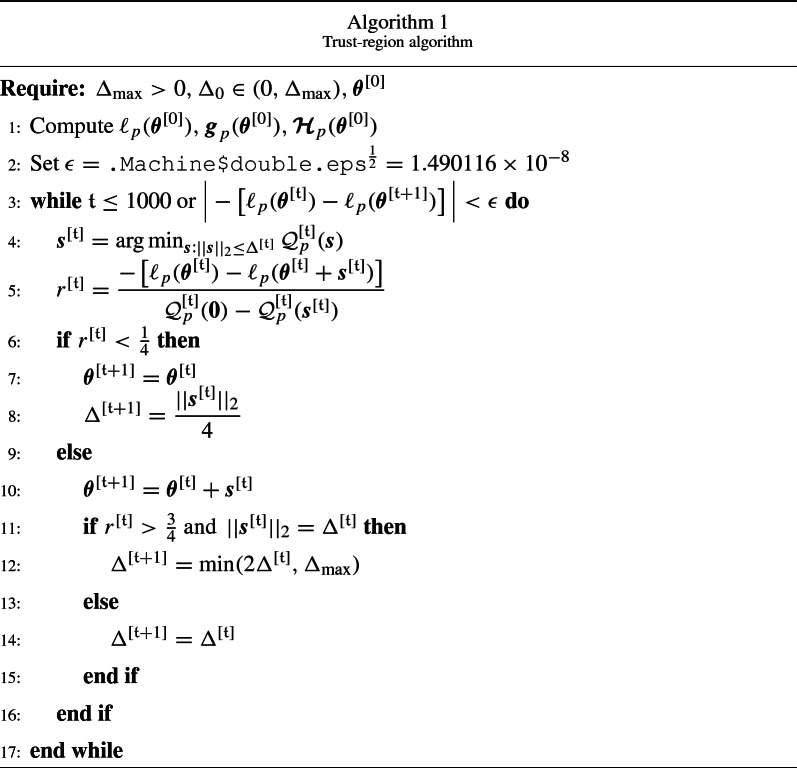


An alternative proposal to using a grid-search combined with GBIC is to estimate ${\varvec{\eta }}$ automatically and in a data-driven fashion, a development that has not been so far considered in penalized factor analysis. To this end, we propose adapting to the current context the automatic multiple tuning (a.k.a smoothing) parameter selection of Marra and Radice (2020, see also references therein), which is based on an approximate AIC.

Assume that, near the solution, the trust-region method behaves like a classic unconstrained Newton-Raphson algorithm (Nocedal & Wright, 2006). Suppose also that ${\varvec{\theta }}^{[{\mathfrak {t}}+1]}$ is the “true” parameter value, and thus ${\varvec{g}}_p({\varvec{\theta }}^{[{\mathfrak {t}}+1]}) = {\varvec{0}}$. By using a first-order Taylor expansion of ${\varvec{g}}_p({\varvec{\theta }}^{[{\mathfrak {t}}+1]})$ at ${\varvec{\theta }}^{[{\mathfrak {t}}]}$ it follows thatSolving for ${\varvec{\theta }}^{[{\mathfrak {t}}]}$ yields, after some manipulation (see Section D.1),23where , ${\varvec{K}}^{[{\mathfrak {t}}]} = {\varvec{\mu }}_{{\varvec{K}}}^{[{\mathfrak {t}}]} + {\varvec{\vartheta }}^{[{\mathfrak {t}}]}$ with  and . The square root of  and its inverse are obtained by eigenvalue decomposition. If they are not positive-definite, they are corrected as described in Section D.2. From standard likelihood theory, we have that ${\varvec{\vartheta }} \sim {\mathcal {N}}\left( {\varvec{0}}, {\varvec{I}}_m \right)$ and ${\varvec{K}} \sim {\mathcal {N}}\left( {\varvec{\mu }}_{{\varvec{K}}}, {\varvec{I}}_m \right)$, where , and ${\varvec{\theta }}_0$ the true parameter vector. Let $\hat{{\varvec{\mu }}}_{{\varvec{K}}}$ be the predicted value vector for ${\varvec{K}}$ defined aswhere  is the influence (or hat) matrix of the fitting problem and depends on the tuning parameters. The quantity  denotes the PMLE. Ideally, the estimation of the tuning parameters should suppress the model complexity unsupported by the data. This can be achieved by minimizing the expected mean squared error of $\hat{{\varvec{\mu }}}_{{\varvec{K}}}$ from its expectation ${\varvec{\mu }}_{{\varvec{K}}}$ (Section D.3):

24 $$\begin{aligned} {\mathbb {E}}\left[ \frac{1}{N} ||{\varvec{\mu }}_{{\varvec{K}}} - \hat{{\varvec{\mu }}}_{{\varvec{K}}}||^2_2 \right] = \frac{1}{N} {\mathbb {E}}\left[ ||{\varvec{K}} - {\varvec{A}}_{{\varvec{\eta }}}^{{\mathcal {T}}} {\varvec{K}}||^2_2 \right] + \frac{2}{N} \text {tr} ({\varvec{A}}_{{\varvec{\eta }}}^{{\mathcal {T}}}) - 1. \end{aligned}$$

The quantity  can be interpreted as the edf of the penalized model, and is equivalent to the expression of the bias term of the GBIC. The right-hand side of (24) depends on the tuning parameters through ${\varvec{A}}_{{\varvec{\eta }}}^{{\mathcal {T}}}$, whereas ${\varvec{K}}$ is linked to the unpenalized part of the model. The tuning parameters are estimated by minimizing an estimate of (24):

25 $$\begin{aligned} {\mathcal {V}}({\varvec{\eta }}) = \frac{1}{N} ||\widehat{{\varvec{\mu }}_{{\varvec{K}}} - \hat{{\varvec{\mu }}}_{{\varvec{K}}}}||^2_2 = \frac{1}{N} ||{\varvec{K}} - {\varvec{A}}_{{\varvec{\eta }}}^{{\mathcal {T}}} {\varvec{K}}||^2_2 + \frac{2}{N} \text {tr} ({\varvec{A}}_{{\varvec{\eta }}}^{{\mathcal {T}}}) - 1. \end{aligned}$$

This is equivalent to the Un-Biased Risk Estimator (UBRE; Wood, 2017, Ch. 6) and an approximate AIC (Section D.4), which means that ${\varvec{\eta }}$ is estimated by minimizing what is effectively the AIC with number of parameters given by $\text {tr}({\varvec{A}}_{{\varvec{\eta }}}^{{\mathcal {T}}})$. In practice, given ${\varvec{\theta }}^{[{\mathfrak {t}}+1]}$, the estimation problem is expressed as

$$\begin{aligned} {\varvec{\eta }}^{[{\mathfrak {t}}+1]} = \arg \min _{{\varvec{\eta }}} {\mathcal {V}}^{[{\mathfrak {t}}+1]}({\varvec{\eta }}) = \arg \min _{{\varvec{\eta }}} \left\{ \frac{1}{N} ||{\varvec{K}}^{[{\mathfrak {t}}+1]} - {\varvec{A}}^{{{\mathcal {T}}}^{[{\mathfrak {t}}+1]}}_{{\varvec{\eta }}} {\varvec{K}}^{[{\mathfrak {t}}+1]}||^2_2 + \frac{2}{N} \text {tr} ({\varvec{A}}^{{{\mathcal {T}}}^{[{\mathfrak {t}}+1]}}_{{\varvec{\eta }}}) - 1 \right\} , \end{aligned}$$

and solved by adapting the approach by Wood (2004) to the current context. This approach is based on Newton’s method and can evaluate in a stable and efficient way the components in ${\mathcal {V}}({\varvec{\eta }})$ and their derivatives with respect to $\log ({\varvec{\eta }})$ (since the tuning parameters can only take positive values). The two steps, one for the estimation of ${\varvec{\theta }}$ and the other for ${\varvec{\eta }}$, are iterated until the algorithm satisfies the stopping criterion $\dfrac{|\ell ({\varvec{\theta }}^{[{\mathfrak {t}}+1]}) - \ell ({\varvec{\theta }}^{[{\mathfrak {t}}]}) |}{0.1 + |\ell ({\varvec{\theta }}^{[{\mathfrak {t}}+1]}) |} < 10^{-7}$.

Sometimes the final model could be overly dense and sparser solutions may be desired. One way to achieve this systematically is to increase the amount that each model edf counts, in the UBRE score, by a factor $\gamma \ge 1$, called “influence factor” (Wood, 2017). The slightly modified tuning criterion then is

26 $$\begin{aligned} {\mathcal {V}}({\varvec{\eta }}) = \frac{1}{N} ||{\varvec{K}} - {\varvec{A}}_{{\varvec{\eta }}}^{{\mathcal {T}}} {\varvec{K}}||^2_2 + \frac{2}{N} \gamma \, \text {tr} ({\varvec{A}}_{{\varvec{\eta }}}^{{\mathcal {T}}}) - 1. \end{aligned}$$

For smoothing spline regression models, Kim and Gu (2004) found that $\gamma = 1.4$ can correct the tendency to over-fitting of prediction error criteria. However, this work deals with different models, and our focus is not only on fit but also on the recovery of sparse structures, thus higher values may be more appropriate.

The automatic procedure described above is general and easy to implement, but it may occasionally suffer at small sample sizes since it finds its justification asymptotically when the dependence of the Hessian on the tuning parameter(s) vanishes. As argued by Wood (2017), at small sample sizes, it would in principle be more reliable to select the tuning parameter(s) based on a non-approximate function, such as the GBIC and grid-searches, although implementing such an approach in the multiple-group context would introduce further complications and possibly new computational problems and instabilities. Notice also that the automatic procedure relies on the separability of the penalty matrix from the tuning parameter(s). This requirement is satisfied by the lasso and alasso (thus, ${\mathcal {T}} = \{L, A\}$), but not by the scad and mcp which are therefore confined to the grid-search approach. However, this is not problematic because in our simulation experiments and empirical application the alasso generally represented the most convenient choice of penalty based on a number of criteria.

At convergence, the covariance matrix of $\hat{{\varvec{\theta }}}$ is . However, instead of ${\varvec{V}}_{\hat{{\varvec{\theta }}}}$, for practical purposes, it is more convenient to employ at convergence the alternative Bayesian result  (Marra & Wood, 2012). The goodness of fit of the penalized model can then be evaluated through confidence intervals, which are available for each model parameter, obtained from the posterior distribution ${\varvec{\theta }} |\{{\varvec{x}}_1, \ldots , {\varvec{x}}_N\}, {\varvec{\eta }} \sim {\mathcal {N}}(\hat{{\varvec{\theta }}}, {\varvec{V}}_{{\varvec{\theta }}})$ (Section D.5). Notice that the proposed approach can be regarded as a Bayesian method with the exponential prior  on the penalty function. The process of determining the optimal loading pattern can indeed be formulated as a Bayesian variable selection problem (Lu, Chow & Loken, 2016). For instance, Bayesian Structural Equation Modeling (BSEM; Muthén & Asparouhov, 2012)—in which the elements that would be fixed to zero in a confirmatory analysis (usually the cross-loadings) are replaced with approximate zeros based on informative, small-variance priors—is a particular case where the shrinkage is achieved through an informative ridge prior. With the proposed method, users can rely on the automatic procedure for recovering optimally sparse factor solutions without manually specifying the variance of the Bayesian prior employed in BSEM.

The presented modeling framework has been implemented in the freely available R package penfa and we refer the reader to Online Resource F for a brief description of the software and practical illustrations.

## Simulation Studies

The performances of the proposed PMLE were evaluated and compared to the penalized methods by Jacobucci et al. (2016, R package regsem) and Huang (2018, R package lslx) in two extensive simulation studies, one for the normal linear factor model and the other for its multiple-group extension. Despite the presence of other penalized factor analysis techniques (Choi et al., 2010; Hirose & Yamamoto 2014b, a; Trendafilov et al., 2017; Jin et al., 2018), our choice fell on regsem and lslx because they allow the specification of fixed, free and penalized parameters, as well the estimation of the structural model.

### Simulation Study I

The first simulation evaluates the performances of the proposed technique in a single-group factor analysis model. We evaluate the impact of several conditions, including the sample size, the penalty function, the type of second-order derivative information used in the trust-region algorithm, the strategy for the choice of the tuning parameter, the magnitude of the influence factor and—for some of the penalties—the value of the additional tuning parameter. The simulation was partly inspired by the empirical application (Sect. 9), therefore the number of variables ($p = 9$) and of factors ($r = 3$) exactly match those of the real data analysis. The conditions that were varied are:

- Sample size: 300, 500, and 1000 observations. These values are in line with those investigated in similar simulation studies (Huang et al., 2017; Jacobucci et al., 2016; Jin et al., 2018; Hirose & Yamamoto, 2014b) and include two moderate sample sizes (which are commonly found in psychometric applications) and a large one (to mimic asymptotic behavior). Note that 300 is close to the number of observations in the empirical example;
- Penalty function: lasso, alasso, scad, and mcp were examined in their ability to shrink to zero small loadings without possibly affecting the remaining ones;
- Information matrix: either the Hessian or the Fisher information matrix was used in the optimization process (see Sect. 7);
- Shrinkage parameter selection: this was achieved either by a grid-search or through the automatic procedure. The grid-search was conducted over 200 distinct values of $\eta$ and for all four penalty types, with the optimal model being the one with the lowest GBIC. The elements of the grid were adapted based on the specific combination of penalty type and sample size. The automatic procedure was used with lasso and alasso;
- Influence factor: informed by the values that performed well in the application, we investigated different values for the influence factor, namely, $\gamma = \{1, 1.4, 2, 2.5, 3, 3.5, 4, 4.5 \}$;
- Additional tuning parameter: we tested different values of the additional tuning parameter of the alasso, scad and mcp. For the alasso $a = \{1, 2\}$, for the scad $a = \{2.5, 3, 3.7, 4.5\}$ (with 3.7 being the conventional level employed in the literature and suggested by Fan & Li, 2001), and for the mcp $a = \{2.5, 3, 3.5\}$.

The population parameters complied to the following structure:

$$\begin{aligned} {\varvec{\Lambda }}^T= & {} \left[ \begin{array}{ccccccccc} 0.85 &{} 0.75 &{} 0.65 &{} {{{\underline{0}}}} &{} 0 &{} 0 &{} {{{\underline{0}}}} &{} 0 &{} 0.30 \\ {{{\underline{0}}}} &{} 0 &{} 0.30 &{} 0.85 &{} 0.75 &{} 0.65 &{} {{{\underline{0}}}} &{} 0 &{} 0 \\ {{{\underline{0}}}} &{} 0 &{} 0 &{} {{{\underline{0}}}} &{} 0 &{} 0.30 &{} 0.85 &{} 0.75 &{} 0.65 \\ \end{array} \right] \\ {\varvec{\Phi }}= & {} \left[ \begin{array}{ccc} {{{\underline{1}}}} &{} 0.3 &{} 0.3 \\ &{} {{{\underline{1}}}} &{} 0.3 \\ &{} &{} {{{\underline{1}}}} \\ \end{array} \right] \end{aligned}$$

with ${\varvec{\Psi }} = {\varvec{I}}_p - {\varvec{\Lambda }} {\varvec{\Phi }} {\varvec{\Lambda }}^T$, where ${\varvec{I}}_p$ is the $p \times p$ identity matrix. Elements in italic and underlined were fixed for scale setting and identification purposes. The specific values of the factor loadings were inspired by the numerical example in Huang et al. (2017). As it is common in many factor analysis applications, a subset of the observed variables does not load only on one factor but also presents a cross-loading.

All of the factor loadings were penalized for assessing the effectiveness of the proposed method in recovering the underlying factor structure and not erroneously shrinking the small cross-loadings to zero. Based on results from previous studies (see for instance Choi et al., 2010 for the alasso, and Hirose & Yamamoto, 2014b and Huang et al., 2017 for the mcp), the alasso and the non-convex penalties are expected to outperform the lasso, which is known to be biased due to its tendency to overly shrink nonzero parameters. Concerning the influence factor, higher values favor sparsity at the expense of an increase in bias, whereas lower values favor goodness of fit.

Data were simulated in R (R Core Team, 2018) according to the population parameters. The resulting data matrix was then analyzed in penfa, regsem (Jacobucci et al., 2019) and lslx (Huang & Hu, 2019) by estimating a factor model with the correct number of factors, the specified fixed elements, and all of the free loadings were penalized. Common factors were estimated to be correlated and with fixed unit variance. Whenever present, sign reversal of the factors was accounted for to ensure that the sign of the primary loadings corresponded to that of the corresponding population parameters. Based on the availability of the respective software implementations,^2^ lasso, alasso, scad and mcp were tried for regsem, and lasso and mcp for lslx. For each scenario, we generated $L =1000$ replications for which the unpenalized factor model produced admissible^3^ solutions.

We evaluated the performance of the methods according to the criteria illustrated in Huang et al. (2017), which are briefly mentioned here. The overall estimation quality was assessed using the estimated mean squared error (MSE):

27 $$\begin{aligned} \widehat{\text {MSE}}(\hat{{\varvec{\theta }}}) = \frac{1}{L} \sum _{l=1}^{L} (\hat{{\varvec{\theta }}}^{(l)} - {\varvec{\theta }}_0 )^T (\hat{{\varvec{\theta }}}^{(l)} - {\varvec{\theta }}_0), \end{aligned}$$

where $\hat{{\varvec{\theta }}}^{(l)} = ({\hat{\theta }}^{(l)}_1, \ldots , {\hat{\theta }}^{(l)}_m)^T$ denotes the vector of estimated parameters in replicate *l*, ${\varvec{\theta }}_0$ the true parameter vector, and *L* the number of replications. The degree of bias of each estimator was evaluated by the estimated squared bias (SB):

28 $$\begin{aligned} \widehat{\text {SB}}(\hat{{\varvec{\theta }}}) = (\bar{\hat{{\varvec{\theta }}}} - {\varvec{\theta }}_0 )^T (\bar{\hat{{\varvec{\theta }}}} - {\varvec{\theta }}_0 ), \end{aligned}$$

where $\bar{\hat{{\varvec{\theta }}}} = \frac{1}{L} \sum _{l=1}^{L} \hat{{\varvec{\theta }}}^{(l)}$ represents the empirical mean of $\hat{{\varvec{\theta }}}$. Let ${\mathcal {F}} = \{q \, |\, \theta _{0 q} \ne 0 \, \& \, {\hat{\theta }}_{q} \text { penalized} \}$ indicate the set of indices associated to the true nonzero parameters that have been penalized (i.e., the penalized nonzero factor loadings) and $|{\mathcal {F}} |$ the cardinality of ${\mathcal {F}}$, which in the simulation is equal to 12. The chance of correctly identifying the true nonzero parameters was evaluated via the estimated true positive rate (TPR):29Denote as ${\mathcal {F}}^c = \{q \, |\, \theta _{0 q} = 0 \, \& \, {\hat{\theta }}_{q} \text { penalized} \}$ the set collecting the indices of the true zero parameters that have been penalized (i.e., the penalized zero factor loadings), with $|{\mathcal {F}}^c |$ equal to 9. The estimated false positive rate (FPR) examined the degree to which the true zero parameters were incorrectly identified as nonzero:30Lastly, selection consistency was assessed via the proportion of times the true model—for which all the true zero and nonzero factor loadings were correctly identified as equal to zero and different from zero, respectively—was chosen over the replicates (proportion choosing the true model; PCTM):31where $|{\mathcal {F}} |+ |{\mathcal {F}}^c |= q^\star$. For the computation of PCTM and FPR,^4^ the parameter estimates were rounded to one decimal digit^5^ for all models. For the sake of clarity, we report a selection of the most relevant results for the configurations of penfa-alasso ($a=2, \gamma = 4.5$), penfa-scad ($a=3$) and penfa-mcp ($a=3$) that produced the best models in terms of the aforementioned performance criteria. Due to its generally higher numerical stability in comparison to the Hessian, only penfa models estimated with the Fisher information matrix are presented. The results for penfa-lasso are described in Online Resource A. In the same spirit, the results of regsem and lslx are presented for their best performing models (i.e., with the mcp for both of them). All other results can be requested from the corresponding author.

Overall, the low values for MSE, the bias, and FPR which are very close to zero, together with high PCTM and excellent TPR show that the examined penalized techniques possess very good empirical performances and outperform the unpenalized (MLE) model (Table 1). The MSE of all penalized methods are very similar to each other and improve as the sample size increased. The results with the lower bias were associated with the use of non-convex penalties, although the bias of penfa-alasso very quickly converged to zero when the sample size increased, and hence the impact of the penalty decreased. The true positive rates were always equal to 1.0, which showed that the inspected methods never suppressed the nonzero penalized parameters (i.e., the primary loadings and the cross-loadings). In terms of both false positive rates and selection consistency, penfa-alasso with automatic tuning parameter selection presented by far the best performances for all the sample sizes. The coverage probabilities for the parameters of all fitted models (Section A.1) were generally close to their true nominal level for all penalty functions.

**Table 1:** Performance measures of the examined models in simulation study I by varying the sample size *N*.

	Unpenalized (MLE)	penfa				lslx	regsem
		ALASSO		SCAD	MCP	MCP	MCP
		Grid	Auto	Grid	Grid	Grid	Grid
MSE							
$N = 300$	0.108	0.073	0.075	0.074	0.074	0.075	0.071
	(0.04–0.41)	(0.02–0.19)	(0.02–0.08)	(0.02–0.17)	(0.02–0.17)	(0.02–0.20)	(0.02–0.33)
$N = 500$	0.064	0.041	0.041	0.042	0.042	0.042	0.041
	(0.02–0.23)	(0.01–0.12)	(0.01–0.12)	(0.01–0.12)	(0.01–0.12)	(0.01–0.13)	(0.01–0.12)
$N = 1000$	0.031	0.020	0.020	0.020	0.020	0.020	0.020
	(0.01–0.08)	(0.01–0.06)	(0.01–0.05)	(0.01–0.06)	(0.01–0.06)	(0.01–0.06)	(0.01–0.06)
SB							
$N = 300$	0.001	0.003	0.004	0.002	0.002	0.003	0.000
$N = 500$	0.000	0.001	0.001	0.001	0.001	0.001	0.000
$N = 1000$	0.000	0.000	0.000	0.000	0.000	0.000	0.000
TPR							
$N = 300$	1	1	1	1	1	1	1
						(0.92–1)	(0.92–1)
$N = 500$	1	1	1	1	1	1	1
$N = 1000$	1	1	1	1	1	1	1
FPR							
$N = 300$	0.390	0.022	0.008	0.016	0.019	0.036	0.018
	(0.00–0.89)	(0.00–0.33)	(0.00–0.22)	(0.00–0.22)	(0.00–0.33)	(0.00–0.44)	(0.00–0.22)
$N = 500$	0.264	0.012	0.004	0.007	0.008	0.016	0.012
	(0.00–0.89)	(0.00–0.22)	(0.000–0.11)	(0.00–0.22)	(0.00–0.22)	(0.00–0.33)	(0.00–0.22)
$N = 1000$	0.113	0.003	0.001	0.002	0.002	0.004	0.009
	(0.00–0.56)	(0.00–0.11)	(0.00–0.11)	(0.00–0.11)	(0.00–0.11)	(0.00–0.22)	(0.00–0.22)
PCTM							
$N = 300$	0.009	0.820	0.932	0.871	0.843	0.743	0.848
$N = 500$	0.073	0.898	0.962	0.936	0.925	0.877	0.897
$N = 1000$	0.356	0.974	0.991	0.982	0.979	0.966	0.923

*MSE* mean-squared error, *SB* squared bias, *TPR* true positive rate, *FPR* false positive rate, *PCTM* proportion choosing the true model. In brackets, the ranges of MSE, TPR, and FPR across replicates.

The mean squared error and bias of penfa-alasso with automatic tuning parameter selection were similar to those obtained with the same penalty and grid-search, but the false positives and PCTM were markedly lower and higher, respectively. This is due to the way the optimal penalized model is picked. With the automatic procedure, the optimal model is the one whose tuning parameter minimizes the criterion in (26); with the grid-search, the optimal model minimizes the GBIC in (19). However refined, a grid-search cannot compete with an approach that looks for the optimal tuning parameter on the positive real line. In addition, the presence of a sparsity-inducing quantity (the influence factor) in the optimization criterion helped the model obtain a nicer tradeoff between goodness of fit and model complexity. With reference to the exponent *a* in the expression of the alasso, as this quantity increased the weights became more influential, and we observed a general improvement in all the performance measures. The best results were obtained for $a = 2$.

By comparing the quality measures of the three methods for the same penalty function (i.e., the mcp), we notice that penfa outperformed lslx and was generally close to regsem for MSE and SB and superior for FPR and PCTM. This might be due to several aspects, e.g., the optimization algorithm, the internal software package implementations, the formulation of the degrees of freedom, and possibly the approximation of the penalty.

The examined performance criteria explored different conflicting objectives. Ideally, one desires a model with low bias and little complexity (i.e., a sparse solution), but the two measures cannot be minimized simultaneously. This can be seen by looking at the performances of the penfa-alasso model for extreme values of the influence factor (i.e., $\gamma = 4.5$ in Table 1 and $\gamma = 1$ in Table A.2 in Section A.2). The higher value of $\gamma$ produced sparser solutions (i.e., smaller FPR and larger PCTM), at the cost of a larger bias. As the sample size increased, the discrepancies in the performances of the models with different values of $\gamma$ diminished though.

The models fitted through the automatic tuning parameter procedure exhibited markedly shorter computational times^6^ than grid-search methods. Specifically, the average median elapsed times were 17 sec for penfa-alasso with grid (1-dim. grid for $\eta$; $a = 2$) and 0.3 sec with automatic procedure ($a = 2; \gamma = 4.5$), 21.1 sec for penfa-scad (1-dim. grid for $\eta$; $a = 3$), 20.7 sec for penfa-mcp (1-dim. grid for $\eta$; $a=3$), 6.6 sec for lslx-mcp (2-dim. grid for $\eta$ and *a*) and 42.2 sec for regsem-mcp (1-dim grid for $\eta$, $a = 3.7$ as per default software implementations). The penfa models with the automatic procedure exhibited the lowest computational times, which is also merit of the stability of the trust-region optimizer, whose parameter updates only involve the points within a proper trust-region. The computational times of lslx are lower than those of the other grid-search techniques because the underlying optimizer is implemented in C++, which significantly boosts the computations with respect to base R routines.

### Simulation Study II

The second simulation evaluates the ability of the proposed technique in identifying the pattern of partial invariance in a multiple-group factor model as a function of the sample size, the size of the generated difference in the group-specific loadings and intercepts, the magnitude of the influence factor and the value of the additional tuning parameter. Since the current implementation of regsem does not allow for multiple-group analyses, our method is only compared with lslx.

**Table 2:** The factor loading matrices and intercepts of the two groups under each difference scenario of simulation study II.

	Group 1			Group 2								
	All conditions			Small			Medium			Large		
	${\varvec{\Lambda }}_1$		${\varvec{\tau }}_1$	${\varvec{\Lambda }}_2$		${\varvec{\tau }}_2$	${\varvec{\Lambda }}_2$		${\varvec{\tau }}_2$	${\varvec{\Lambda }}_2$		${\varvec{\tau }}_2$
$x_1$	*0.85*	*0*	*0*	*0.85*	*0*	*0*	*0.85*	*0*	*0*	*0.85*	*0*	*0*
$x_2$	0.85	0	0	0.85	0	0	0.85	0	0	0.85	0	0
$x_3$	0.85	0	0	0.85	0	0	0.85	0	0	0.85	0	0
$x_4$	0.75	0	0	0.75	0	0	0.75	0	0	0.75	0	0
$x_5$	0.75	0	0	0.75	0	0	0.75	0	0	0.75	0	0
$x_6$	0.75	0	0	0.65	0	− 0.1	0.55	0	− 0.2	0.45	0	− 0.3
$x_7$	*0*	*0.85*	*0*	*0*	*0.85*	*0*	*0*	*0.85*	*0*	*0*	*0.85*	*0*
$x_8$	0	0.85	0	0	0.85	0	0	0.85	0	0	0.85	0
$x_9$	0	0.85	0	0	0.85	0	0	0.85	0	0	0.85	0
$x_{10}$	0	0.75	0	0	0.75	0	0	0.75	0	0	0.75	0
$x_{11}$	0	0.75	0	0	0.75	0	0	0.75	0	0	0.75	0
$x_{12}$	0	0.75	0	0	0.65	− 0.1	0	0.55	− 0.2	0	0.45	− 0.3

Elements fixed for origin and scale setting and identification purposes are italic and underlined. Under the null condition, the parameters of Group 2 coincide with those of Group 1.

We consider a population multiple-group factor model with $p = 12$ variables, $r=3$ factors and $G=2$ groups. We explore a range of conditions, under which the factor loading matrices and intercepts are either invariant or non-invariant, with the level of non-invariance becoming progressively larger. Based on the findings from Simulation study I, we employ the alasso penalty for inducing sparsity and invariant loadings and intercepts, that is, . The three tuning parameters $(\eta _1, \eta _2, \eta _3)^T$ in ${\varvec{\eta }}$ are estimated alongside the model parameters through the automatic multiple tuning parameter procedure. For lslx we used the mcp penalty, which had better performances than the lasso. The optimization technique currently employed in lslx makes use of a single penalty for both shrinking the parameters and their differences across groups. Therefore, there is only one shrinkage parameter $\eta$, whose optimal value is determined through a grid-search. For lslx-mcp, we carried out a grid-search over 200 values of the shrinkage parameter $\eta$ and 4 of the shape parameter *a*. The conditions that were varied are:

- Sample size: 300, 500, and 1000 observations evenly split between the two groups, with 300 being close to the number of observations in the empirical example;
- Difference size: either null, small, medium or large group differences in the primary loadings and the intercepts of two variables were created (details are given below). This condition was partly inspired by the simulation conducted by Huang (2018);
- Influence factor: informed by the values that performed well in Simulation study I, we investigated three values of the influence factor, namely, $\gamma = \{3.5, 4, 4.5\}$;
- Additional tuning parameter: two values were tested for the exponent in the expression of the alasso, namely $a = \{1,2\}$.

The factor loading matrix and the vector of intercepts of Group 1 are reported on the left-hand side of Table 2, and are the same under every difference scenario. Elements in italic and underlined are fixed for metric setting and identification purposes. The factor loadings and intercepts of Group 2 are presented by difference scenario on the right-hand side of Table 2. In case of a null difference, the two groups share the same parameter matrices. Under the small, medium and large scenarios, the primary loadings and the intercepts of two variables (i.e., $x_6$ and $x_{12}$) in Group 1 differ from the corresponding parameters in Group 2 by a size of 0.1, 0.2, and 0.3, respectively. Under all conditions, the structural parameters are assumed to be invariant across groups, that is, $\text {vech}({\varvec{\Phi }}_1) = \text {vech}({\varvec{\Phi }}_2) = \text {vech}({\varvec{\Phi }}) = (1, 0.3, 1)^T$ and ${\varvec{\kappa }}_1 = {\varvec{\kappa }}_2 = (0, 0)^T$, whereas ${\varvec{\Psi }}_g = {\varvec{I}}_p - {\varvec{\Lambda }}_g {\varvec{\Phi }} {\varvec{\Lambda }}_g^T$, for $g = 1, 2$. The factor loadings and the intercepts are penalized in the way described in Sect. 5 (i.e., shrinkage of the loadings and of the pairwise group differences of loadings and intercepts), whereas the remaining model parameters are estimated without penalization. For each scenario, we generated $L =1000$ replications for which the unpenalized multiple-group model produced admissible solutions, and analyzed them as in simulation study I.

The performances of the penalized models are evaluated through the criteria (27)-(31) used in simulation study I. For the sake of conciseness, we report the results for the penfa-alasso model ($a = 2, \gamma = 4.5$) that produced the best solution in terms of these performance criteria. All other results can be requested from the corresponding author. Overall, the low values of MSE, SB, FPR, high PCTM and excellent TPR show that the penalized techniques possess very good empirical performances, with all measures improving as the sample size increased (Table 3). Higher difference sizes were associated with higher MSE and squared bias, with the lower values generally occurring for penfa-alasso. We separately computed these measures for each parameter matrix (that is, ${\varvec{\Lambda }}_g$, ${\varvec{\tau }}_g$, ${\varvec{\Psi }}_g$, ${\varvec{\Phi }}_g$, ${\varvec{\kappa }}_g$, for $g = 1, 2$) produced by penfa-alasso. The largest MSE were observed for the factor variances and covariances, followed by the factor loadings. The bias tended to increase for the penalized parameters (factor loadings and intercepts) across the difference conditions, while remaining almost unaltered for the unique variances and the structural parameters. The squared bias quickly converged towards zero in all difference scenarios as the sample size increased. The TPR were always equal to 1.0, which showed that the examined methods never suppressed the nonzero penalized parameters.

**Table 3:** Performance measures of penfa-alasso ($a = 2, \gamma = 4.5$) and lslx-mcp models by sample size and difference scenario.

Difference scenario	Null		Small		Medium		Large	
	penfa	lslx	penfa	lslx	penfa	lslx	penfa	lslx
MSE								
$N = 300$	0.275	0.279	0.303	0.307	0.356	0.372	0.385	0.416
	(0.11–0.65)	(0.10–0.75)	(0.12–0.76)	(0.11–0.79)	(0.15–0.83)	(0.19–0.86)	(0.15–0.90)	(0.14–1.04)
$N = 500$	0.165	0.164	0.189	0.189	0.220	0.239	0.221	0.235
	(0.07–0.50)	(0.06–0.47)	(0.08–0.54)	(0.08–0.51)	(0.11–0.60)	(0.12–0.58)	(0.09–0.60)	(0.08–0.52)
$N = 1000$	0.083	0.082	0.102	0.104	0.105	0.115	0.103	0.101
	(0.04–0.20)	(0.04–0.20)	(0.05–0.22)	(0.05–0.22)	(0.04–0.25)	(0.04–0.26)	(0.04–0.26)	(0.04–0.23)
SB								
$N = 300$	0.003	0.002	0.020	0.021	0.046	0.062	0.043	0.050
$N = 500$	0.001	0.001	0.017	0.020	0.026	0.042	0.018	0.012
$N = 1000$	0.000	0.000	0.012	0.018	0.007	0.007	0.005	0.001
TPR								
$N = 300$	1	1	1	1	1	1	1	1
$N = 500$	1	1	1	1	1	1	1	1
$N = 1000$	1	1	1	1	1	1	1	1
FPR								
$N = 300$	0.006	0.010	0.005	0.012	0.005	0.019	0.004	0.035
	(0.00–0.40)	(0.00–0.30)	(0.00–0.20)	(0.00–0.30)	(0.00–0.20)	(0.000–0.30)	(0.00–0.20)	(0.00–0.40)
$N = 500$	0.004	0.004	0.005	0.005	0.004	0.014	0.003	0.020
	(0.00–0.20)	(0.00–0.20)	(0.00–0.30)	(0.00–0.20)	(0.00–0.20)	(0.000–0.25)	(0.00–0.20)	(0.00–0.25)
$N = 1000$	0.002	0.001	0.002	0.002	0.002	0.005	0.001	0.003
	(0.00–0.15)	(0.00–0.10)	(0.00–0.20)	(0.00–0.15)	(0.00–0.20)	(0.000–0.15)	(0.00–0.20)	(0.00–0.15)
PCTM								
$N = 300$	0.935	0.890	0.945	0.880	0.933	0.820	0.948	0.677
$N = 500$	0.951	0.956	0.948	0.949	0.947	0.854	0.967	0.781
$N = 1000$	0.980	0.991	0.969	0.977	0.976	0.930	0.984	0.958

*MSE* mean-squared error, *SB* squared bias, *TPR* true positive rate, *FPR* false positive rate, *PCTM* proportion choosing the true model. In brackets, the ranges of MSE and FPR across replicates; TPR were always equal to one.

Whereas under the null and small scenarios the two methods produced similar measures, penfa-alasso markedly outperformed lslx-mcp under the medium and large conditions, especially in terms of selection consistency at the smallest sample size. On top of that, whereas these performance measures for lslx noticeably degraded as the difference size increased, they remained fairly stable for penfa-alasso; even with the smallest sample size, penfa-alasso identified the true heterogeneity pattern more than 90% of the times. Thanks to the use of the automatic multiple tuning parameter procedure, the average median computational time to fit a penfa-alasso model with 3 tuning parameters (3.2 seconds) was much lower than the one necessary to fit an lslx-mcp model with a single shrinkage parameter $\eta$ and the associated shape parameter *a* selected through a grid-search (45 seconds).

## Empirical Application

The Holzinger & Swineford data set (Holzinger & Swineford, 1939; Kelley, 2019) is a classical psychometric application containing the responses of $N = 301$ students on some psychological tests. This data set (or subsets of it) has been often used to demonstrate CFA (Jöreskog, 1979), EFA (Browne, 2001; Jöreskog & Sörbom, 1993) and various penalized factor analysis techniques (Trendafilov et al., 2017; Jacobucci et al., 2016; Huang et al., 2017; Jin et al., 2018). For space constraints, the description of the data set is reported in Online Resource E.

### Normal Linear Factor Model

Following Jacobucci et al. (2016) and Huang et al. (2017), to illustrate the proposed method in the normal linear factor model, we use a subset of nine mental tests (VISUAL, CUBES, FLAGS, PARAGRAP, SENTENCE, WORDM, ADDITION, COUNTING, and STRAIGHT) underlying three latent factors. The data set was column-wise centered since the model in equation (1) assumes that the observed variables have zero means and scaled as described in Yuan and Bentler (2006). The inspection at the covariance matrix of the observed variables revealed the presence of relationships between tests designed to measure distinct mental abilities. The CFA model assuming a simple structure showed a poor fit to the data (p-value of the chi-square goodness of fit test < 0.001), which suggested the multi-dimensionality of some of the tests. In these circumstances where it may be difficult to specify the correct sparsity pattern of the loading matrix in advance, it is beneficial to resort to penalized techniques to explore and unveil the underlying loading pattern. We hence penalize all of the factor loadings and freely estimate the remaining model parameters. Factor variances are fixed to one for scale setting and some elements of the loading matrix to zero for identification purposes. Even if the proposed method does allow us to obtain sparsity, we should acknowledge that its achievement also depends on the features of the statistical model under investigation and the amount of information carried by the data. Concerning the former, as pointed out by Trendafilov et al. (2017), inducing sparsity in a factor model, and even more so one with correlated factors, is more complicated than for other types of models (e.g. principal component analysis) due to the presence of other parameters (unique variances and factor variances and covariances) affecting the overall model fit. As a result, if too large a value for the tuning parameter is chosen, an excessive number of loadings is shrunken, and the remaining parameters are forced to explode to compensate for this lack of fit. This issue can be avoided if the appropriate amount of sparsity is introduced into the model, which in turn is only possible if the tuning parameter governing the amount of sparsity is selected according to a valid procedure, such as the one introduced in the paper.

We fitted a large number of models involving all four penalties. For grid-search, 200 models corresponding to varying levels of the tuning parameter were fitted. We also tried a sequence of values for the additional tuning parameter of the alasso ($a =\{ 1, 1.5, 2\}$), scad ($a =\{ 2.5, 3.7, 4.5\}$), mcp ($a = \{1.5, 2, 2.5, 3, 3.5\}$), and for the influence factor ($\gamma = \{1, 1.4, 2, 2.5, 3, 3.5, 4, 4.5\}$). The GBIC^7^ values were calculated for each of the fitted penfa models and are ranked in Table 4 for the best model configurations. In particular, the alasso (automatic procedure, $a = 1, \gamma = 4.5$) presented the lowest BIC, closely followed by the mcp ($a = 1.5$) and scad ($a = 4.5$). Interestingly, the BIC of penfa-lasso with grid-search (7567.62) markedly decreased when the model was fitted through the automatic procedure with an influence factor of 4.5 (7562.94). Notice that both the CFA and the unpenalized solution (corresponding to the factor analysis model in equation (1) with the minimum identification restrictions) resulted in worse fits than the ones of the penalized models, probably because of the strict assumption of no cross-loadings of the former, and the unnecessary complexity of the latter. This indicates that the analysis benefited from the introduction of sparsity.

**Table 4:** BIC of the best configurations of the fitted models.

Method	Penalty	BIC
penfa	ALASSO	7558.03
penfa	MCP	7561.57
penfa	SCAD	7561.68
penfa	LASSO	7562.94
CFA		7595.34
Unpenalized		7601.42

For penfa-alasso (automatic procedure) $a = 1$ and $\gamma = 4.5$, for penfa-scad $a = 4.5$, for penfa-mcp $a = 1.5$, and for penfa-lasso (automatic procedure) $\gamma = 4.5$. For all models the Fisher information was used.

Table 5 (left-hand side) reports the parameter estimates of the unpenalized model and the best penfa-alasso model. A blank cell in the factor loading matrix indicates that the corresponding estimate was zero after one decimal digit rounding.^8^ The unpenalized model presented various cross-loadings, which resulted in a complex model. For penfa, only four secondary loadings (${\hat{\lambda }}_{51}$, ${\hat{\lambda }}_{81}$, ${\hat{\lambda }}_{91}$, ${\hat{\lambda }}_{32}$) were identified as nonzero. If a sparser loading matrix is desired, users can increase the value of the exponent *a* of the alasso and/or the influence factor $\gamma$ in the automatic procedure. For instance, a penfa-alasso model (BIC = 7565.39) with $a = 2$ and $\gamma = 5.5$ (Table 5, right-hand side) produced a sparser factor solution with a single cross-loading (${\hat{\lambda }}_{91}$). The data analysis was also conducted for regsem and lslx using the available penalties (i.e., lasso, alasso, scad, and mcp for the former, and lasso and mcp for the latter) and is presented in Online Resource E. The factor structures of the penalized models looked similar, but the proposed method reported the lowest BIC values, showing the potential of the presented procedure. As argued by Huang et al. (2017), this example shows that complex models do not necessarily outperform simpler ones when model complexity is also taken into account in the model selection criterion.

**Table 5:** Parameter estimates of the nine mental tests from the Holzinger & Swineford data set for the unpenalized model, and penfa-alasso with automatic procedure (on the left-hand side, ${\hat{\eta }} = 0.017, a = 1$ and $\gamma = 4.5$; on the right-hand side, ${\hat{\eta }} = 0.011, a = 2$ and $\gamma = 5.5$).

Measurement model	Unpenalized model				penfa-alasso ($a = 1, \gamma = 4.5$)				penfa-alasso ($a = 2, \gamma = 5.5$)			
	Spatial	Verbal	Speed	${\varvec{\Psi }}$	Spatial	Verbal	Speed	${\varvec{\Psi }}$	Spatial	Verbal	Speed	${\varvec{\Psi }}$
VISUAL	0.81	*0*	*0*	0.70	0.83	*0*	*0*	0.63	0.85	*0*	*0*	0.59
CUBES	0.65	− 0.12	− 0.16	1.03	0.49			1.11	0.46			1.13
FLAGS	0.91	− 0.33		0.69	0.76	− 0.16		0.75	0.66			0.82
PARAGRAP	*0*	0.99	*0*	0.38	*0*	0.96	*0*	0.38	*0*	0.96	*0*	0.37
SENTENCE	− 0.13	1.19		0.40	− 0.06	1.11		0.42		1.08		0.44
WORDM	0.07	0.87		0.37		0.89		0.36		0.89		0.36
ADDITION	*0*	*0*	0.77	0.59	*0*	*0*	0.70	0.67	*0*	*0*	0.62	0.76
COUNTING	0.30	− 0.16	0.68	0.48	0.12		0.70	0.44			0.79	0.36
STRAIGHT	0.54	− 0.14	0.43	0.55	0.41		0.42	0.56	0.36		0.39	0.58

Structural model	Spatial	Verbal	Speed		Spatial	Verbal	Speed		Spatial	Verbal	Speed	
Spatial	*1*	0.59	0.17		*1*	0.48	0.20		*1*	0.45	0.31	
Verbal	–	*1*	0.22		–	*1*	0.16		–	*1*	0.19	
Speed	–	–	*1*		–	–	*1*		–	–	*1*	

Fixed parameters are italic and underlined. A blank cell indicates that the corresponding estimate is zero.

### Multiple-Group Factor Model

Besides considering the sample of the students as a whole, we divided it into two groups ($N_1 = 156, N_2 = 145$) based on the attended school, and then conducted a multiple-group analysis. One school (Pasteur) included students with parents who immigrated from Europe, whereas the other (Grant-White) was composed of students coming from middle-income American white families. Following Huang (2018), we considered the 19 mental tests and standardized the data to handle the scaling effect.

The traditional approach consists of the estimation of an unpenalized multiple-group CFA in which the tests are assumed to be pure measures, followed by factorial invariance testing procedures. The model assuming equal loadings across groups shows an adequate fit to the data (p-value of the chi-square goodness of fit test = 0.266), which, however, significantly worsens when the intercepts are also equated across groups (p-value of the likelihood ratio test comparing the model with invariant loadings and intercepts versus the one with only invariant loadings < 0.001). Model modifications are typically conducted to determine and freely estimate the non-invariant elements.

Alternatively, the invariance pattern can be explored via penalized techniques employing penalties that combine sparsity and cross-group equivalence of loadings and intercepts. In light of its superior performance in the single-group analysis and simulation, we employed the alasso with the automatic multiple tuning parameter procedure, and tested various values of the influence factor $(\gamma = \{1, 2, 3, 3.5, 4, 4.5\})$ and the exponent $(a =\{ 1, 2\})$. The tests VISUAL, WORDM, COUNTING and NUMBERR are assumed to be the markers, and thus have fixed loadings and intercepts. The data analysis was also conducted in lslx with the mcp (see Table E.4), but not in regsem as its current implementation does not allow for multiple-group analyses. Note that lslx uses only one penalty for shrinking both the parameters and their differences, hence it has a single tuning parameter $\eta$.

The parameter estimates of penfa-alasso are reported in Table 6. The better fit of penfa-alasso ($\text {BIC} = 14658$) as compared to lslx-mcp ($\text {BIC} = 14697.75$) is also merit of the greater flexibility of the former, which employs three distinct penalties having their own tuning parameters, with respect to the latter, where a single tuning has to take care of the shrinkage of the parameters as well as their cross-group differences. penfa-alasso produces sparse loading matrices with many zero-entries, but the presence of a couple of nonzero cross-loadings demonstrates that the structure hypothesized by a multiple-group CFA is too restrictive. The factor loading matrices of penfa-alasso are also fully equivalent, in agreement to the results of invariance testing. Conversely, the intercepts are not fully invariant, which is again in line with the findings from factorial invariance testing. This example clearly shows the benefits of using properly designed penalized techniques to explore the non-equivalence pattern of the parameter matrices in a multiple-group factor model.

**Table 6:** Parameter estimates of the 19 mental tests from the Holzinger & Swineford data set for penfa-alasso (automatic procedure, $\hat{{\varvec{\eta }}} = (0.006, 16221.852, 0.013)^T, a = 1, \gamma = 4$)

Measurement model	penfa - alasso											
	Pasteur School						Grant-White School					
	${\varvec{\tau }}_1$	Spatial	Verbal	Speed	Memory	${\varvec{\Psi }}_1$	${\varvec{\tau }}_2$	Spatial	Verbal	Speed	Memory	${\varvec{\Psi }}_2$
VISUAL	*0*	*1*	*0*	*0*	*0*	0.44	*0*	*1*	*0*	*0*	*0*	0.43
CUBES	0.01	0.58				0.89	0.01	0.58				0.68
PAPER	0	0.62				0.81	0	0.62				0.71
FLAGS	0.14$^\star$	0.86	− 0.09			0.61	− 0.16$^\star$	0.86	− 0.09			0.47
GENERAL	− 0.01		1.02		− 0.11	0.26	− 0.01		1.02		− 0.11	0.31
PARAGRAP	− 0.01		0.96			0.35	−0.01		0.96			0.31
SENTENCE	− 0.01	− 0.12	1.08			0.25	− 0.01	− 0.12	1.08			0.22
WORDC	− 0.08$^\star$		0.84			0.41	0.07$^\star$		0.84			0.45
WORDM	*0*	*0*	*1*	*0*	*0*	0.23	*0*	*0*	*1*	*0*	*0*	0.35
ADDITION	0.14$^\star$	− 0.40	0.14	0.99	0.15	0.52	− 0.18$^\star$	− 0.40	0.14	0.99	0.15	0.34
CODE	0		0.17	0.74	0.27	0.44	0		0.17	0.74	0.27	0.61
COUNTING	*0*	*0*	*0*	*1*	*0*	0.54	*0*	*0*	*0*	*1*	*0*	0.44
STRAIGHT	0	0.40		0.68		0.62	0	0.40		0.68		0.44
WORDR	*0*	*0*	*0*	*0*	*1*	0.58	*0*	*0*	*0*	*0*	*1*	0.56
NUMBERR	0		− 0.14		0.84	0.68	0		− 0.14		0.84	0.67
FIGURER	0.02	0.37			0.63	0.73	0.02	0.37			0.63	0.47
OBJECT	0.16$^\star$	− 0.23		0.32	0.87	0.63	− 0.19$^\star$	− 0.23		0.32	0.87	0.46
NUMBERF	0			0.25	0.65	0.78	0			0.25	0.65	0.65
FIGUREW	− 0.20$^\star$	0.06		0.09	0.53	0.85	0.24$^\star$	0.06		0.09	0.53	0.60
Spatial	− 0.02	0.59	0.28	0.16	0.17		0.02	0.60	0.36	0.29	0.24	
Verbal	− 0.26	−	0.66	0.19	0.10		0.29	−	0.62	0.23	0.26	
Speed	0.09	−	−	0.44	0.07		− 0.09	−	−	0.63	0.16	
Memory	− 0.05	−	−	−	0.52		0.05	−	−	−	0.42	

Fixed parameters are italic and underlined. A blank cell in the factor loading matrix indicates that the corresponding estimate is zero. Non-invariant parameters across groups are starred ($^\star$).

## Discussion

Penalized factor analysis is an efficient estimation technique that produces a factor loading matrix with many zero elements thanks to the introduction of sparsity-inducing penalty functions within the estimation process. In order to achieve sparse solutions and stable model selection procedures, the penalty functions must be non-differentiable. In this work, we adopted suitable local approximations of them. In this way, it was possible to employ in the optimization process a trust-region algorithm, which required analytical information on the score vector and the Hessian matrix (or a good approximation thereof). The use of differentiable penalties allowed us to recast the problem in a theoretically founded framework, where a precise definition of effective degrees of freedom was obtained, based on the bias term of the Generalized Information Criterion, or equivalently, the influence matrix of the model. This represents a novelty, as the existing proposals compute the degrees of freedom of a penalized factor model as the number of nonzero parameters. As an alternative to the usually time-consuming grid-searches, we also illustrated an efficient automatic technique for the estimation of the tuning parameter alongside the parameters of the factor model. The asymptotic properties of the penalized estimator can be established along the lines of Filippou et al. (2017) and Fan and Li (2001).

The simulations showed that the proposed approach produced trustworthy models with high accuracy, selection consistency, low bias and false positives. This indicates that the method is a valuable alternative to the existing techniques. Furthermore, it often generated the best tradeoff between goodness of fit and model complexity when compared to such models, as in the empirical application. As a result of this delicate balance, the proposed method may not necessarily provide the sparsest factor solution. Numerical experiments, however, confirm that the proposed method can produce very good results even if the penalized parameters are estimated just close enough to zero. This is because the edf are also being estimated close to zero, and we would actually need a considerable number of coefficients to see a substantive impact on the total edf and the GBIC. Still, if researchers desire more sparsity, they can manually and subjectively increase the value of the tuning parameter or the influence factor for the automatic procedure.

Notably, we extended the illustrated framework to multiple-group factor models by employing a penalty that simultaneously induced sparsity and cross-group equality of loadings and intercepts. As such, it revealed as a worthy alternative to invariance testing procedures. In this context, the automatic procedure proved particularly useful as it allowed for the estimation of the multiple tuning parameters composing the penalty term in a fast, stable and efficient way.

The presented framework allows one to easily and efficiently combine multiple penalty terms (like in the multiple-group model), as the automatic procedure scales well with the number of tuning parameters. In the empirical application, the alasso penalty was considered for all three penalty terms, but different penalty functions can also be combined if desired.

Another interesting modification pertains to the type of parameters that are penalized. Given the general estimation framework proposed in this work, also residual covariances (i.e., the off-diagonal elements of the covariance matrix of the unique factors) can be penalized to examine the assumption of conditional independence (that is, detect which pairs of variables are conditionally dependent). This model is known in the econometric literature as “sparse approximate factor model” (Bai & Liao, 2016).

We envisage several interesting lines of future research. Firstly, the proposed approach can be applied to structural equation models in which, in addition to the measurement model, a structural model (usually a mediation model for the factors) is tested. Secondly, the results described in this work were derived under the $N > p$ scenario, as it is the case for many applications from the social and behavioral sciences. However, penalized techniques can also be extremely useful in the high-dimensional case, where maximum likelihood estimation is not feasible. It would hence be interesting to review the presented methodology in this demanding set-up. Future research may also evaluate the impact of messy data and larger model sizes on the penalized estimation framework. Finally, the observed variables were assumed to follow a multivariate normal distribution. When this is not reasonable, one can resort to pseudo maximum likelihood (Arminger & Schoenberg, 1989) or, for categorical data, pairwise maximum likelihood (Katsikatsou et al., 2012). Further studies are needed to extend this work to the non-normal case, as this setting poses additional challenges since the asymptotic covariance matrix of the PMLE is no longer consistently estimated by the inverse Fisher information but by a “sandwich-type” covariance matrix (Yuan & Bentler 1997).

## Supplementary Information

Below is the link to the electronic supplementary material.

Supplementary material 1 (pdf 984 KB)

## Footnotes

1 An alternative factor model formulation would include the intercepts of the observed variables and the factor means. See the multiple-group extension in Sect. 4 for an example of a mean and covariance structure model.

2 For regsem, we used the default optimizer Rsolnp for lasso and alasso, and coordinate descent for scad and mcp. The additional tuning parameters of the penalties were kept to the values specified in the package, that is, $a = 1$ for alasso, and $a = 3.7$ for scad and mcp. For lslx, as per software implementations, the shape parameter of the mcp was internally selected over a varying three-dimensional grid of values adapted for each replicate. Because of the specificities of each package implementation, a customized and sensible grid of $\eta$ values was considered for each technique.

3 A solution is considered admissible if it does not present Heywood cases (negative unique variances), the covariance matrices of the unique factors and common factors are positive-definite, the factor loading matrix is of full column rank and does not contain any null rows (Jöreskog & Sörbom, 1996).

4 No rounding was necessary for TPR because, based on the simulation design, the estimates were never mistakenly estimated close to zero.

5 This choice was made on practical grounds, deeming estimates in absolute value below 0.05 to be “practically” and “substantively” equal to zero. The use of tighter rounding thresholds may worsen FPR and PCTM. For penfa, numerical experiments showed that this was the case when using the grid-search, whereas the models with the automatic procedure exhibited fairly comparable FPR and PCTM even after two or three decimal digits roundings.

6 All computations were carried out on a machine with Intel(R) Core(TM) i7-5600U 2.60GHz (quad-core) processor and 16GB of RAM.

7 We used the BIC as a criterion for model comparisons due to its widespread use in sparse settings, but different evaluation measures can be employed depending on the research question.

8 This was done for a neater visual illustration; in the penfa package, no internal rounding is implemented, and the estimates are shown as returned by the trust-region optimizer.
