# Supplementary material for: Single- and Multiple-Group Penalized Factor Analysis: A Trust-Region Algorithm Approach with Integrated Automatic Multiple Tuning Parameter Selection
Source: Psychometrika. 2021 Mar 26;86(1):65–95. doi: 10.1007/s11336-021-09751-8 (PMC8035122; doi:10.1007/s11336-021-09751-8)
Supplement: Supplementary file 1 — Supplementary material 1 (pdf 984 KB) [file 11336_2021_9751_MOESM1_ESM.pdf]

Online Resources for
"Single and multiple-group penalized factor analysis: a trust-region algorithm approach with integrated automatic multiple tuning parameter selection"

Online Resource A Simulations

We report some further results on simulation study I. We first discuss the coverage probabilities (Section A.1) of the models illustrated in Section 8.1, then we present the performance measures of some additional models (Section A.2).

A.1 Coverage probabilities

We computed 95% coverage probabilities for the parameters of all penalized models using point-wise confidence intervals (Table A.1). For clarity of presentation, we only report the inferential results of the models considered in Table 1. The omitted tables can be requested from the corresponding author. The standard errors for `penfa` are based on the Bayesian result illustrated in Section D.5. On the contrary, for `lslx`, they are computed using the frequentist expression of the covariance matrix based on the Fisher information. No coverage probabilities could be computed for `regsem` as the package does not currently provide any measure of uncertainty. Because of the rationale discussed in Section 7, `penfa` provides a standard error for every single model parameter, contrarily to `lslx` which does not provide this information for the parameters shrunken to zero. However, since the main intent of penalization is to get rid of the uninfluential elements, the inferential results are presented for the parameters remaining in the model, which are the effective quantities of interest. The coverage probabilities were furtherly split and averaged between those corresponding to the penalized parameters (i.e., the non-zero factor loadings) and the freely estimated ones (i.e., the factor covariances and unique variances). Overall, the values of both `penfa` and `lslx` are close to their true nominal level, the more so as the sample size increases, for all penalty functions, which proves that the selected models are also valid from an inferential point of view.

Sample size	penfa								lslx	
	**ALASSO**				**SCAD**		**MCP**		**MCP**	
	grid		auto		grid		grid		grid	
	Pen.	Free	Pen.	Free	Pen.	Free	Pen.	Free	Pen.	Free
$N = 300$	0.922	0.942	0.900	0.942	0.916	0.942	0.918	0.942	0.924	0.942
$N = 500$	0.934	0.946	0.931	0.946	0.929	0.945	0.928	0.945	0.938	0.945
$N = 1000$	0.940	0.946	0.940	0.946	0.941	0.945	0.940	0.945	0.945	0.946

Note: Pen. indicates the penalized non-zero parameters and *free* the freely estimated parameters.

Table A.1: Average coverage probabilities of the examined penalized models in simulation study I by sample size and parameter type. For `penfa-alasso` with grid $a = 2$, with the automatic procedure $a = 2$ and $\gamma = 4.5$, for `penfa-scad` $a = 3$ and for `penfa-mcp` $a = 3$.

A.2 Additional models

The influence factor γ plays a decisive role in the model fitting results. Table A.2 reports the performance measures of the `penfa-alasso` model ($a = 2$) for $\gamma = 1$. The `penfa-alasso` model with the larger γ (Table 1) resulted in visibly higher PCTM and lower FPR, at the expense of a slight increase in bias. This loss in bias, however, became negligible or nonexistent as the sample size grew. In this respect, it is interesting to look at the MSE, which encloses both the variance and the squared bias of an estimator. Despite the model with $\gamma = 1$ always had a smaller bias, the one with $\gamma = 4.5$ produced such a decrease in the variability of the estimates that its MSE ended up being always smaller than the one obtained with the inferior value of the influence factor. The TPR were equal to 1.0 for every sample size.

We conclude the simulation results with the performances of the `penfa-lasso` models for which the tuning parameter was either selected by grid-search or estimated through the automatic procedure (Table A.2). The two models gave overall similar results, with the former having better FPR and PCTM and the latter lower MSE and bias. The TPR were equal to 1.0 in both cases and for every sample size. These results, however, are visibly less performing than the models where the alasso, scad and mcp were used. As a matter of fact, it is well known that the lasso tends to select an overfitted model, because it equally penalizes all model parameters. Therefore, we suggest opting for the other penalties, which have been specifically designed to improve the lasso.

	ALASSO	LASSO	
	auto $\gamma = 1$	grid	auto $\gamma = 4.5$
**MSE**			
$N = 300$	0.083	0.109	0.102
$N = 500$	0.049	0.066	0.061
$N = 1000$	0.024	0.034	0.031
**SB**			
$N = 300$	0.001	0.039	0.030
$N = 500$	0.000	0.024	0.017
$N = 1000$	0.000	0.013	0.008
**FPR**			
$N = 300$	0.154	0.094	0.113
$N = 500$	0.114	0.060	0.074
$N = 1000$	0.049	0.017	0.026
**PCTM**			
$N = 300$	0.256	0.409	0.321
$N = 500$	0.374	0.583	0.493
$N = 1000$	0.634	0.860	0.795

Table A.2: Performance measures of `penfa-alasso` and `penfa-lasso` in simulation study I by the sample size N. The quantity γ denotes the influence factor. MSE stands for mean-squared error, SB for squared bias, FPR for false positive rate and PCTM for proportion choosing the true model.

Online Resource B Locally approximated penalties

We describe the process for formulating and locally approximating the employed penalty functions for single and multiple-group factor analysis models (Sections B.1 and B.2, respectively).

B.1 Normal linear factor model

We first provide an example clarifying the formulation of the sparsity-inducing penalties for the normal linear factor model (Section B.1.1), we then formulate their expressions for the lasso, alasso, scad and mcp functions (Section B.1.2), and lastly we derive their local approximations (Section B.1.3).

B.1.1 Example

For notational clarity, we illustrate the general structure of the penalty described in Section 3 in a simple example for the normal linear factor model. Consider the factor analysis model $\boldsymbol{x} = \boldsymbol{\Lambda f} + \boldsymbol{\varepsilon}$, with $p = 6$ observed variables and $r = 2$ common factors, where it is assumed that $\boldsymbol{f} \sim \mathcal{N}(\mathbf{0}, \boldsymbol{\Phi})$, $\boldsymbol{\varepsilon} \sim \mathcal{N}(\mathbf{0}, \boldsymbol{\Psi})$ with $\boldsymbol{\Psi}$ a diagonal matrix, and $\boldsymbol{f}$ is independent of $\boldsymbol{\varepsilon}$. The population parameters are as follows:

$$\boldsymbol{\Lambda} = \begin{bmatrix} \lambda_{11} & \underline{\mathit{0}} \\ \lambda_{21} & \lambda_{22} \\ \lambda_{31} & \lambda_{32} \\ \underline{\mathit{0}} & \lambda_{42} \\ \lambda_{51} & \lambda_{52} \\ \lambda_{61} & \lambda_{62} \end{bmatrix} \qquad \boldsymbol{\Psi} = \begin{bmatrix} \psi_{11} & 0 & 0 & 0 & 0 & 0 \\ & \psi_{22} & 0 & 0 & 0 & 0 \\ & & \psi_{33} & 0 & 0 & 0 \\ & & & \psi_{44} & 0 & 0 \\ & & & & \psi_{55} & 0 \\ & & & & & \psi_{66} \end{bmatrix} \qquad \boldsymbol{\Phi} = \begin{bmatrix} \underline{\mathit{1}} & \phi_{12} \\ & \underline{\mathit{1}} \end{bmatrix},$$

where the elements in italic and underlined were fixed for scale setting and identification purposes. The vector $\boldsymbol{\theta}$ collects the free parameters in $\mathrm{vec}(\boldsymbol{\Lambda})$, $\mathrm{diag}(\boldsymbol{\Psi})$, and $\mathrm{vech}(\boldsymbol{\Phi})$, that is:

$$\boldsymbol{\theta} = (\lambda_{11}, \lambda_{21}, \lambda_{31}, \lambda_{51}, \lambda_{61}, \lambda_{22}, \lambda_{32}, \lambda_{42}, \lambda_{52}, \lambda_{62}, \psi_{11}, \psi_{22}, \psi_{33}, \psi_{44}, \psi_{55}, \psi_{66}, \phi_{12})^T.$$

Conveniently, the parameter vector can be rewritten as

$$\boldsymbol{\theta} = (\underbrace{\theta_1, \theta_2, \theta_3, \theta_4, \theta_5, \theta_6, \theta_7, \theta_8, \theta_9, \theta_{10}}_{\textit{Factor loadings}}, \theta_{11}, \theta_{12}, \theta_{13}, \theta_{14}, \theta_{15}, \theta_{16}, \theta_{17})^T,$$

where the sub-vector $(\theta_1, \dots, \theta_{10})^T$ collects the parameters that are being penalized (i.e., the factor loadings), whereas $(\theta_{11}, \dots, \theta_{17})^T$ the unpenalized parameters (i.e., the free elements in $\boldsymbol{\Psi}$ and $\boldsymbol{\Phi}$). We consider the case where the interest lies in the shrinkage of the factor loadings, although other model parameters could be in principle penalized. Let $q^\star = 10$ be the number of penalized parameters, and $m = 17$ the total number of parameters. Define

$$\boldsymbol{R}_q = \begin{array}{c} \\ 1 \\ \\ q \\ \\ \\ 17 \end{array} \begin{array}{c} \begin{array}{ccccc} 1 & & q & & 17 \end{array} \\ \begin{bmatrix} 0 & \dots & 0 & \dots & \dots & 0 \\ \vdots & \ddots & \vdots & & & \vdots \\ 0 & \dots & 1 & \dots & \dots & 0 \\ \vdots & & \vdots & \ddots & & \vdots \\ \vdots & & \vdots & & \ddots & \vdots \\ 0 & \dots & 0 & \dots & \dots & 0 \end{bmatrix} \end{array} \qquad \text{for } q = 1, \dots, 10,$$

and $\boldsymbol{R}_q = \boldsymbol{O}_{17 \times 17}$ for $q = 11, \dots, 17$. Then, the sparsity-inducing penalty is expressed as $\mathcal{P}_\eta(\boldsymbol{\theta}) = \sum_{q=1}^{17} \mathcal{P}_{\eta,q}(||\boldsymbol{R}_q \boldsymbol{\theta}||_1)$, where $||\boldsymbol{R}_q \boldsymbol{\theta}||_1 = |\theta_q|$ for $q = 1, \dots, 10$, and 0 for $q = 11, \dots, 17$.

B.1.2 The penalty functions

Let us write the parameter vector as $\boldsymbol{\theta} = (\theta_1, \dots, \theta_{q^\star}, \theta_{q^\star+1}, \dots, \theta_m)^T$, where the sub-vector $(\theta_1, \dots, \theta_{q^\star})^T$ collects the penalized parameters (i.e., the factor loadings), whereas $(\theta_{q^\star+1}, \dots, \theta_m)^T$ the unpenalized parameters (i.e., the free elements in $\boldsymbol{\Phi}$ and $\boldsymbol{\Psi}$). Define the diagonal matrix $\boldsymbol{R}_q = \text{diag}(0, 0, \dots, 0,\ 1, 0, \dots, 0)$ for $q = 1, \dots, q^\star$ where the 1 on the $(q,q)^{\text{th}}$ entry of the matrix corresponds to the qth parameter in $\boldsymbol{\theta}$, and $\boldsymbol{R}_q = \boldsymbol{O}_{m \times m}$ for $q = q^\star + 1, \dots, m$. Let $\boldsymbol{e}_q = (0, \dots, 0, 1, 0, \dots, 0)^T$ be the canonical vector with a 1 in the qth position for $q = 1, \dots, q^\star$, and the null vector otherwise. In this work, we employ the lasso, alasso, scad and mcp penalties

on the factor loadings, whose expressions are derived below. The overall penalty $\mathcal{T}$ is given by the sum of the penalty terms for each parameter, that is,

$$\mathcal{P}_{\eta}^{\mathcal{T}}(\boldsymbol{\theta}) = \sum_{q=1}^{m} \mathcal{P}_{\eta,q}^{\mathcal{T}}(||\boldsymbol{R}_q\boldsymbol{\theta}||_1),$$

where $\mathcal{T} = \{L, A, S, M\}$ stands for lasso, alasso, scad, and mcp, respectively. The term $||\boldsymbol{R}_q\boldsymbol{\theta}||_1 = |\boldsymbol{e}_q^T\boldsymbol{\theta}| = |\theta_q|$ for $q = 1, \ldots, q^\star$, and is equal to zero otherwise. Let us detail the expression of the penalty term for each of these penalties.

Lasso

$$\begin{aligned}
\mathcal{P}_{\eta}^{L}(\boldsymbol{\theta}) &= \sum_{q=1}^{m} \mathcal{P}_{\eta,q}^{L}(||\boldsymbol{R}_q\boldsymbol{\theta}||_1) = \sum_{q=1}^{m} \eta||\boldsymbol{R}_q\boldsymbol{\theta}||_1 = \eta\sum_{q=1}^{m} \left\{(\boldsymbol{R}_q\boldsymbol{\theta})^T(\boldsymbol{R}_q\boldsymbol{\theta})\right\}^{\frac{1}{2}} \\
&= \eta\sum_{q=1}^{m}\left\{(\boldsymbol{e}_q^T\boldsymbol{\theta})^2\right\}^{\frac{1}{2}} = \eta\sum_{q=1}^{m}|\boldsymbol{e}_q^T\boldsymbol{\theta}| = \eta\sum_{q=1}^{q^\star}|\theta_q|.
\end{aligned}$$

Alasso

$$\begin{aligned}
\mathcal{P}_{\eta}^{A}(\boldsymbol{\theta}) &= \sum_{q=1}^{m} \mathcal{P}_{\eta,q}^{A}(||\boldsymbol{R}_q\boldsymbol{\theta}||_1) = \eta\sum_{q=1}^{m}\frac{||\boldsymbol{R}_q\boldsymbol{\theta}||_1}{||\boldsymbol{R}_q\hat{\boldsymbol{\theta}}||_1^a} = \eta\sum_{q=1}^{m}\frac{\left\{(\boldsymbol{R}_q\boldsymbol{\theta})^T(\boldsymbol{R}_q\boldsymbol{\theta})\right\}^{\frac{1}{2}}}{\left\{\left(\boldsymbol{R}_q\hat{\boldsymbol{\theta}}\right)^T\left(\boldsymbol{R}_q\hat{\boldsymbol{\theta}}\right)\right\}^{\frac{a}{2}}} \\
&= \eta\sum_{q=1}^{m}\frac{\left\{(\boldsymbol{e}_q^T\boldsymbol{\theta})^2\right\}^{\frac{1}{2}}}{\left\{\left(\boldsymbol{e}_q^T\hat{\boldsymbol{\theta}}\right)^2\right\}^{\frac{a}{2}}} = \eta\sum_{q=1}^{m}\frac{|\boldsymbol{e}_q^T\boldsymbol{\theta}|}{\left|\boldsymbol{e}_q^T\hat{\boldsymbol{\theta}}\right|^a} = \eta\sum_{q=1}^{q^\star}\frac{|\theta_q|}{|\hat{\theta}_q|^a},
\end{aligned}$$

where $\hat{\boldsymbol{\theta}}$ is generally the maximum likelihood estimator $\hat{\boldsymbol{\theta}}^{\text{MLE}}$ and $a > 0$ an additional tuning parameter.

Scad

$$\begin{aligned}
\mathcal{P}_{\eta}^{S}(\boldsymbol{\theta}) = \sum_{q=1}^{m} \mathcal{P}_{\eta,q}^{S}(||\boldsymbol{R}_q\boldsymbol{\theta}||_1) = \sum_{q=1}^{m}\Bigg\{&\eta||\boldsymbol{R}_q\boldsymbol{\theta}||_1\mathbb{1}\left(0 \le ||\boldsymbol{R}_q\boldsymbol{\theta}||_1 \le \eta\right) \\
&- \left[\frac{(\boldsymbol{R}_q\boldsymbol{\theta})^T(\boldsymbol{R}_q\boldsymbol{\theta}) + \eta^2 - 2\eta a||\boldsymbol{R}_q\boldsymbol{\theta}||_1}{2(a-1)}\right] \\
&\times \mathbb{1}\left(\eta < ||\boldsymbol{R}_q\boldsymbol{\theta}||_1 \le a\eta\right) \\
&+ \frac{\eta^2(a+1)}{2}\mathbb{1}\left(||\boldsymbol{R}_q\boldsymbol{\theta}||_1 > a\eta\right)\Bigg\}
\end{aligned}$$

$$
\begin{aligned}
&= \sum_{q=1}^{m} \Bigg\{ \eta \left[(\boldsymbol{R}_q \boldsymbol{\theta})^T (\boldsymbol{R}_q \boldsymbol{\theta}) \right]^{\frac{1}{2}} \mathbb{1} \left(0 \leq \left[(\boldsymbol{R}_q \boldsymbol{\theta})^T (\boldsymbol{R}_q \boldsymbol{\theta}) \right]^{\frac{1}{2}} \leq \eta \right) \\
&\qquad - \left[\frac{(\boldsymbol{R}_q \boldsymbol{\theta})^T (\boldsymbol{R}_q \boldsymbol{\theta}) + \eta^2 - 2\eta a \left[(\boldsymbol{R}_q \boldsymbol{\theta})^T (\boldsymbol{R}_q \boldsymbol{\theta}) \right]^{\frac{1}{2}}}{2(a-1)} \right] \\
&\qquad + \frac{\eta^2 (a+1)}{2} \mathbb{1} \left(\left[(\boldsymbol{R}_q \boldsymbol{\theta})^T (\boldsymbol{R}_q \boldsymbol{\theta}) \right]^{\frac{1}{2}} > a\eta \right) \Bigg\} \\
&= \sum_{q=1}^{m} \Bigg\{ \eta \left[(\boldsymbol{e}_q^T \boldsymbol{\theta})^2 \right]^{\frac{1}{2}} \mathbb{1} \left(0 \leq \left[(\boldsymbol{e}_q^T \boldsymbol{\theta})^2 \right]^{\frac{1}{2}} \leq \eta \right) \\
&\qquad - \left[\frac{(\boldsymbol{e}_q^T \boldsymbol{\theta})^2 + \eta^2 - 2\eta a \left[(\boldsymbol{e}_q^T \boldsymbol{\theta})^2 \right]^{\frac{1}{2}}}{2(a-1)} \right] \mathbb{1} \left(\eta < \left[(\boldsymbol{e}_q^T \boldsymbol{\theta})^2 \right]^{\frac{1}{2}} \leq a\eta \right) \\
&\qquad + \frac{\eta^2 (a+1)}{2} \mathbb{1} \left(\left[(\boldsymbol{e}_q^T \boldsymbol{\theta})^2 \right]^{\frac{1}{2}} > a\eta \right) \Bigg\} \\
&= \sum_{q=1}^{m} \Bigg\{ \eta |\boldsymbol{e}_q^T \boldsymbol{\theta}| \mathbb{1} \left(0 \leq |\boldsymbol{e}_q^T \boldsymbol{\theta}| \leq \eta \right) - \left[\frac{(\boldsymbol{e}_q^T \boldsymbol{\theta})^2 + \eta^2 - 2\eta a |\boldsymbol{e}_q^T \boldsymbol{\theta}|}{2(a-1)} \right] \mathbb{1} \left(\eta < |\boldsymbol{e}_q^T \boldsymbol{\theta}| \leq a\eta \right) \\
&\qquad + \frac{\eta^2 (a+1)}{2} \mathbb{1} \left(|\boldsymbol{e}_q^T \boldsymbol{\theta}| > a\eta \right) \Bigg\} \\
&= \sum_{q=1}^{q^\star} \Bigg\{ \eta |\theta_q| \mathbb{1} (0 \leq |\theta_q| \leq \eta) - \left[\frac{\theta_q^2 + \eta^2 - 2\eta a |\theta_q|}{2(a-1)} \right] \mathbb{1} (\eta < |\theta_q| \leq a\eta) + \frac{\eta^2 (a+1)}{2} \big(|\theta_q| > a\eta \big) \Bigg\},
\end{aligned}
$$

where $a > 2$ is an additional tuning parameter.

Mcp

$$
\begin{aligned}
\mathcal{P}_\eta^M(\boldsymbol{\theta}) &= \sum_{q=1}^{m} \mathcal{P}_{\eta,q}^M (||\boldsymbol{R}_q \boldsymbol{\theta}||_1) \\
&= \sum_{q=1}^{q^\star} \Bigg\{ \left(\eta ||\boldsymbol{R}_q \boldsymbol{\theta}||_1 - \frac{(\boldsymbol{R}_q \boldsymbol{\theta})^T (\boldsymbol{R}_q \boldsymbol{\theta})}{2a} \right) \mathbb{1} \left(0 \leq ||\boldsymbol{R}_q \boldsymbol{\theta}||_1 \leq a\eta \right) + \frac{\eta^2 a}{2} \mathbb{1} \left(||\boldsymbol{R}_q \boldsymbol{\theta}||_1 > a\eta \right) \Bigg\} \\
&= \sum_{q=1}^{m} \Bigg\{ \left(\eta \left[(\boldsymbol{R}_q \boldsymbol{\theta})^T (\boldsymbol{R}_q \boldsymbol{\theta}) \right]^{\frac{1}{2}} - \frac{(\boldsymbol{R}_q \boldsymbol{\theta})^T (\boldsymbol{R}_q \boldsymbol{\theta})}{2a} \right) \mathbb{1} \left(0 \leq \left[(\boldsymbol{R}_q \boldsymbol{\theta})^T (\boldsymbol{R}_q \boldsymbol{\theta}) \right]^{\frac{1}{2}} \leq a\eta \right) \\
&\qquad + \frac{\eta^2 a}{2} \mathbb{1} \left(\left[(\boldsymbol{R}_q \boldsymbol{\theta})^T (\boldsymbol{R}_q \boldsymbol{\theta}) \right]^{\frac{1}{2}} > a\eta \right) \Bigg\} \\
&= \sum_{q=1}^{m} \Bigg\{ \left(\eta \left[(\boldsymbol{e}_q^T \boldsymbol{\theta})^2 \right]^{\frac{1}{2}} - \frac{(\boldsymbol{e}_q^T \boldsymbol{\theta})^2}{2a} \right) \mathbb{1} \left(0 \leq \left[(\boldsymbol{e}_q^T \boldsymbol{\theta})^2 \right]^{\frac{1}{2}} \leq a\eta \right) + \frac{\eta^2 a}{2} \mathbb{1} \left(\left[(\boldsymbol{e}_q^T \boldsymbol{\theta})^2 \right]^{\frac{1}{2}} > a\eta \right) \Bigg\} \\
&= \sum_{q=1}^{m} \Bigg\{ \left(\eta |\boldsymbol{e}_q^T \boldsymbol{\theta}| - \frac{(\boldsymbol{e}_q^T \boldsymbol{\theta})^2}{2a} \right) \mathbb{1} \left(0 \leq |\boldsymbol{e}_q^T \boldsymbol{\theta}| \leq a\eta \right) + \frac{\eta^2 a}{2} \mathbb{1} \left(|\boldsymbol{e}_q^T \boldsymbol{\theta}| > a\eta \right) \Bigg\}
\end{aligned}
$$

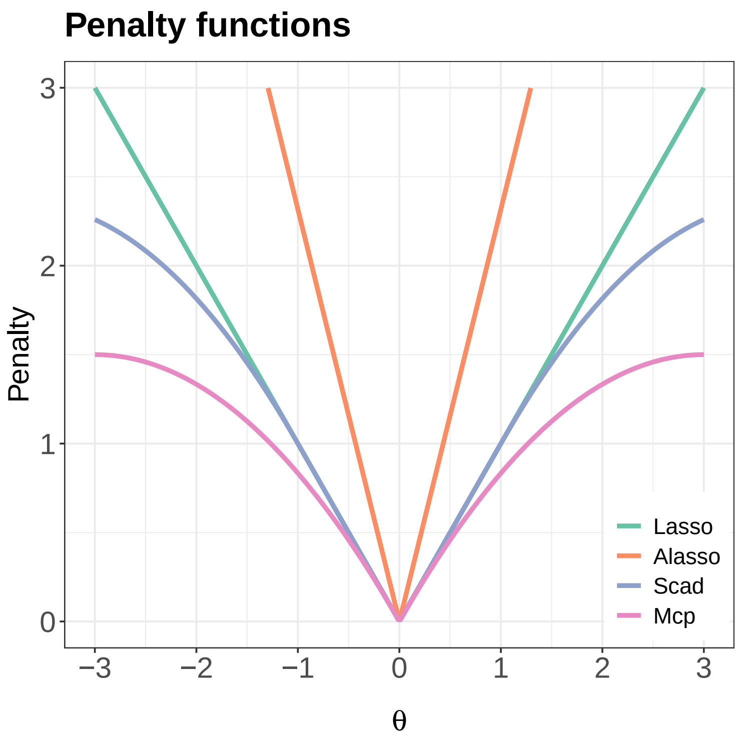

Figure B.1: Shapes of the lasso, alasso ($a = 1$), scad ($a = 3.7$) and mcp ($a = 3$) penalty functions for $\eta = 1$.

$$= \sum_{q=1}^{q^\star} \left\{ \left(\eta|\theta_q| - \frac{\theta_q^2}{2a} \right) \mathbb{1}(0 \le |\theta_q| \le a\eta) + \frac{\eta^2 a}{2} \mathbb{1}(|\theta_q| > a\eta) \right\},$$

where $a > 1$ is an additional tuning parameter.

In the expressions of the penalties $\mathcal{P}_\eta^A(\boldsymbol{\theta}), \mathcal{P}_\eta^S(\boldsymbol{\theta}), \mathcal{P}_\eta^M(\boldsymbol{\theta})$, we did not stress their dependence on the additional tuning parameter a because this quantity is implicitly assumed to be fixed, for instance, it has been determined from prior trials. Common values of the shape parameter of the scad range between 2.5 and 4.5 (Huang, Chen & Weng, 2017), with 3.7 being the conventional level employed in the literature and suggested by Fan and Li (2001). For the mcp, values of a between 1.5 and 3.5 are often considered (Huang, 2018), whereas the exponent of the alasso does not typically exceed 2 (Zou, 2006).

Simplified examples of the shapes of the illustrated penalties are shown in Figure B.1. For all penalties $\eta = 1$, whereas the shape parameter for the scad is $a = 3.7$, for the mcp is $a = 3$, and the exponent of the alasso is $a = 1$. All of the four penalties belong to the L_1-type family and are non-differentiable. Contrarily to the lasso and alasso, the depicted scad and mcp penalties are concave functions.

Figure B.2 illustrates the shapes of the alasso, scad and mcp by varying the value of their additional tuning parameter a. The exponent in the expression of the alasso controls the importance given to the adaptive weights. As the exponent a gets larger, the relative strength of the penalization increases for smaller maximum likelihood estimates compared to larger maximum likelihood estimates. The shapes of the scad and mcp are similar, with their degree of concavity decreasing as the shape parameter a increases. When $a \to \infty$ (see for instance, $a = 50$), the two penalties converge to the lasso.

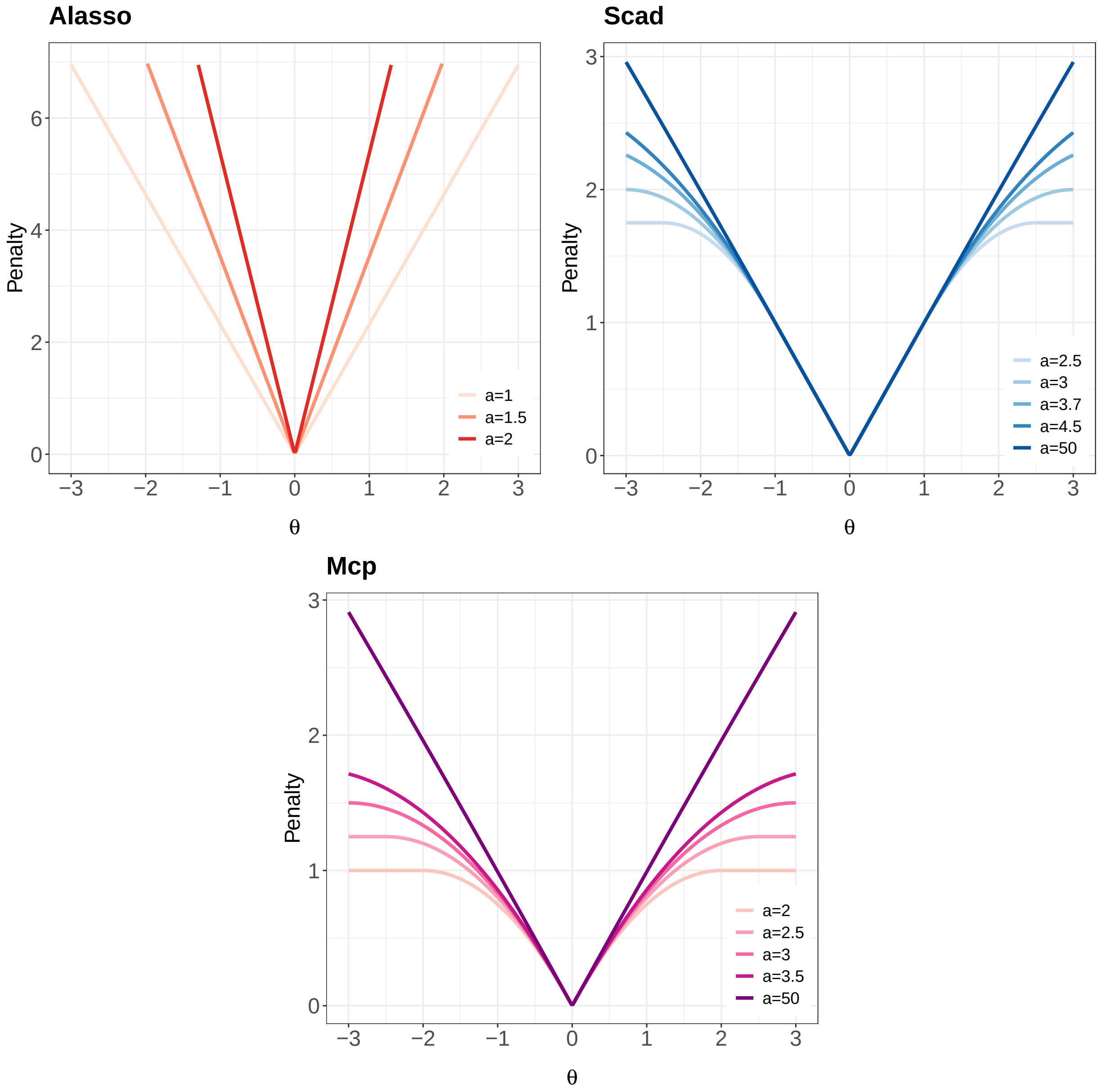

Figure B.2: The alasso, scad and mcp penalties by varying the value of their additional tuning parameter a.

B.1.3 Differentiable approximations of non-differentiable penalties

The penalties examined in this work, i.e., lasso, alasso, scad and mcp, belong to the L_1-type family and are non-differentiable. Given that their non-differentiability poses theoretically and computational challenges, we propose to replace them with their differentiable local approximations.

Based on the first-order Taylor expansion presented in equation (7) and by applying the chain rule, the penalty $\mathcal{P}_\eta^{\mathcal{T}}(\boldsymbol{\theta})$ can be written as

$$\begin{aligned}
\mathcal{P}_\eta^{\mathcal{T}}(\boldsymbol{\theta}) &\approx \mathcal{P}_\eta^{\mathcal{T}}(\tilde{\boldsymbol{\theta}}) + \nabla_{\tilde{\boldsymbol{\theta}}}\mathcal{P}_\eta^{\mathcal{T}}(\tilde{\boldsymbol{\theta}})^T(\boldsymbol{\theta}-\tilde{\boldsymbol{\theta}}) \\
&\approx \mathcal{P}_\eta^{\mathcal{T}}(\tilde{\boldsymbol{\theta}}) + \frac{\partial \mathcal{P}_\eta^{\mathcal{T}}(\tilde{\boldsymbol{\theta}})^T}{\partial \tilde{\boldsymbol{\theta}}}(\boldsymbol{\theta}-\tilde{\boldsymbol{\theta}}) \\
&\approx \mathcal{P}_\eta^{\mathcal{T}}(\tilde{\boldsymbol{\theta}}) + \sum_{q=1}^{m}\left[\frac{\partial \mathcal{P}_{\eta,q}^{\mathcal{T}}(||\boldsymbol{R}_q\tilde{\boldsymbol{\theta}}||_1)}{\partial \tilde{\boldsymbol{\theta}}}\right]^T(\boldsymbol{\theta}-\tilde{\boldsymbol{\theta}}) \\
&\approx \mathcal{P}_\eta^{\mathcal{T}}(\tilde{\boldsymbol{\theta}}) + \sum_{q=1}^{m}\left[\frac{\partial \mathcal{P}_{\eta,q}^{\mathcal{T}}(||\boldsymbol{R}_q\tilde{\boldsymbol{\theta}}||_1)}{\partial ||\boldsymbol{R}_q\tilde{\boldsymbol{\theta}}||_1}\right]^T \cdot \left[\frac{\partial ||\boldsymbol{R}_q\tilde{\boldsymbol{\theta}}||_1}{\partial \boldsymbol{R}_q\tilde{\boldsymbol{\theta}}}\right]^T \cdot \left[\frac{\partial \boldsymbol{R}_q\tilde{\boldsymbol{\theta}}}{\partial \tilde{\boldsymbol{\theta}}}\right]^T(\boldsymbol{\theta}-\tilde{\boldsymbol{\theta}}),
\end{aligned} \tag{B.1}$$

where $\tilde{\boldsymbol{\theta}}$ is an initial value close to the true value of $\boldsymbol{\theta}$.

Let us examine the quantities that make up each addend of expression (B.1). The first factor represents the derivative of $\mathcal{P}_{\eta,q}^{\mathcal{T}}(\tilde{\boldsymbol{\theta}})$ with respect to the L_1-norm of its argument $\boldsymbol{R}_q\tilde{\boldsymbol{\theta}}$. Because the expression depends on the specific form of the penalty $\mathcal{T}$, it is separately computed for each of the examined penalties in Section B.1.3.1. The second factor denotes the derivative of the L_1-norm with respect to its argument, and is equal for all penalties to

$$\begin{aligned}
\frac{\partial ||\boldsymbol{R}_q\tilde{\boldsymbol{\theta}}||_1}{\partial \boldsymbol{R}_q\tilde{\boldsymbol{\theta}}} &= \frac{\partial}{\partial \boldsymbol{R}_q\tilde{\boldsymbol{\theta}}}\left\{\sum_{s=1}^{m}\left[(\boldsymbol{R}_s\tilde{\boldsymbol{\theta}})^T\boldsymbol{R}_s\tilde{\boldsymbol{\theta}}\right]^{\frac{1}{2}}\right\} = \frac{\partial}{\partial \boldsymbol{R}_q\tilde{\boldsymbol{\theta}}}\left[(\boldsymbol{R}_q\tilde{\boldsymbol{\theta}})^T\boldsymbol{R}_q\tilde{\boldsymbol{\theta}}\right]^{\frac{1}{2}} \\
&= \frac{1}{2}\left[(\boldsymbol{R}_q\tilde{\boldsymbol{\theta}})^T\boldsymbol{R}_q\tilde{\boldsymbol{\theta}}\right]^{-\frac{1}{2}} \cdot 2\boldsymbol{R}_q\tilde{\boldsymbol{\theta}} = [(\boldsymbol{R}_q\tilde{\boldsymbol{\theta}})^T\boldsymbol{R}_q\tilde{\boldsymbol{\theta}}]^{-\frac{1}{2}}\boldsymbol{R}_q\tilde{\boldsymbol{\theta}} \\
&\approx \frac{1}{\sqrt{(\boldsymbol{R}_q\tilde{\boldsymbol{\theta}})^T\boldsymbol{R}_q\tilde{\boldsymbol{\theta}}+\bar{c}}}\boldsymbol{R}_q\tilde{\boldsymbol{\theta}},
\end{aligned}$$

where the denominator is approximated by $\sqrt{(\boldsymbol{R}_q\tilde{\boldsymbol{\theta}})^T\boldsymbol{R}_q\tilde{\boldsymbol{\theta}}+\bar{c}}$ to allow for the case of $\tilde{\boldsymbol{\theta}} = \boldsymbol{0}$. Finally, the third factor is simply $\dfrac{\partial \boldsymbol{R}_q\tilde{\boldsymbol{\theta}}}{\partial \tilde{\boldsymbol{\theta}}} = \boldsymbol{R}_q$.

By combining the local approximation $(\boldsymbol{R}_q\boldsymbol{\theta}) \approx (\boldsymbol{R}_q\tilde{\boldsymbol{\theta}})$ (Fan & Li, 2001) with the following

approximation introduced in Ulbricht (2010):

$$
\begin{aligned}
(\boldsymbol{R}_q\boldsymbol{\theta})^T\boldsymbol{R}_q(\boldsymbol{\theta}-\tilde{\boldsymbol{\theta}}) &= (\boldsymbol{R}_q\boldsymbol{\theta})^T\boldsymbol{R}_q\boldsymbol{\theta} - (\boldsymbol{R}_q\boldsymbol{\theta})^T\boldsymbol{R}_q\tilde{\boldsymbol{\theta}} \\
&= \frac{1}{2}\left\{(\boldsymbol{R}_q\boldsymbol{\theta})^T\boldsymbol{R}_q\boldsymbol{\theta} - 2(\boldsymbol{R}_q\boldsymbol{\theta})^T\boldsymbol{R}_q\tilde{\boldsymbol{\theta}} + (\boldsymbol{R}_q\tilde{\boldsymbol{\theta}})^T\boldsymbol{R}_q\tilde{\boldsymbol{\theta}}\right\} \\
&\quad + \frac{1}{2}\left\{(\boldsymbol{R}_q\boldsymbol{\theta})^T\boldsymbol{R}_q\boldsymbol{\theta} - (\boldsymbol{R}_q\tilde{\boldsymbol{\theta}})^T\boldsymbol{R}_q\tilde{\boldsymbol{\theta}}\right\} \\
&= \frac{1}{2}\left\{(\boldsymbol{\theta}-\tilde{\boldsymbol{\theta}})^T\boldsymbol{R}_q^T\boldsymbol{R}_q(\boldsymbol{\theta}-\tilde{\boldsymbol{\theta}})\right\} + \frac{1}{2}\left\{(\boldsymbol{R}_q\boldsymbol{\theta})^T\boldsymbol{R}_q\boldsymbol{\theta} - (\boldsymbol{R}_q\tilde{\boldsymbol{\theta}})^T\boldsymbol{R}_q\tilde{\boldsymbol{\theta}}\right\} \\
&\approx \frac{1}{2}\left(\boldsymbol{\theta}^T\boldsymbol{R}_q^T\boldsymbol{R}_q\boldsymbol{\theta} - \tilde{\boldsymbol{\theta}}^T\boldsymbol{R}_q^T\boldsymbol{R}_q\tilde{\boldsymbol{\theta}}\right),
\end{aligned}
$$

we have that

$$
\begin{aligned}
&\left[\frac{\partial\mathcal{P}_{\eta,q}^{\mathcal{T}}(||\boldsymbol{R}_q\tilde{\boldsymbol{\theta}}||_1)}{\partial||\boldsymbol{R}_q\tilde{\boldsymbol{\theta}}||_1}\right]^T \cdot \left[\frac{\partial||\boldsymbol{R}_q\tilde{\boldsymbol{\theta}}||_1}{\partial\boldsymbol{R}_q\tilde{\boldsymbol{\theta}}}\right]^T \cdot \left[\frac{\partial\boldsymbol{R}_q\tilde{\boldsymbol{\theta}}}{\partial\tilde{\boldsymbol{\theta}}}\right]^T (\boldsymbol{\theta}-\tilde{\boldsymbol{\theta}}) \\
= \; & \frac{\partial\mathcal{P}_{\eta,q}^{\mathcal{T}}(||\boldsymbol{R}_q\tilde{\boldsymbol{\theta}}||_1)}{\partial||\boldsymbol{R}_q\tilde{\boldsymbol{\theta}}||_1} \cdot \left[\frac{\partial||\boldsymbol{R}_q\tilde{\boldsymbol{\theta}}||_1}{\partial\boldsymbol{R}_q\tilde{\boldsymbol{\theta}}}\right]^T \cdot \frac{\partial\boldsymbol{R}_q\tilde{\boldsymbol{\theta}}}{\partial\tilde{\boldsymbol{\theta}}}(\boldsymbol{\theta}-\tilde{\boldsymbol{\theta}}) \\
= \; & \frac{\partial\mathcal{P}_{\eta,q}^{\mathcal{T}}(||\boldsymbol{R}_q\tilde{\boldsymbol{\theta}}||_1)}{\partial||\boldsymbol{R}_q\tilde{\boldsymbol{\theta}}||_1} \cdot \frac{1}{\sqrt{(\boldsymbol{R}_q\tilde{\boldsymbol{\theta}})^T\boldsymbol{R}_q\tilde{\boldsymbol{\theta}}+\bar{c}}}(\boldsymbol{R}_q\tilde{\boldsymbol{\theta}})^T \cdot \boldsymbol{R}_q(\boldsymbol{\theta}-\tilde{\boldsymbol{\theta}}) \\
\approx \; & \frac{\partial\mathcal{P}_{\eta,q}^{\mathcal{T}}(||\boldsymbol{R}_q\tilde{\boldsymbol{\theta}}||_1)}{\partial||\boldsymbol{R}_q\tilde{\boldsymbol{\theta}}||_1} \cdot \frac{1}{\sqrt{(\boldsymbol{R}_q\tilde{\boldsymbol{\theta}})^T\boldsymbol{R}_q\tilde{\boldsymbol{\theta}}+\bar{c}}}\frac{1}{2}\left(\boldsymbol{\theta}^T\boldsymbol{R}_q^T\boldsymbol{R}_q\boldsymbol{\theta} - \tilde{\boldsymbol{\theta}}^T\boldsymbol{R}_q^T\boldsymbol{R}_q\tilde{\boldsymbol{\theta}}\right) \\
= \; & \frac{1}{2}\boldsymbol{\theta}^T\left\{\frac{\partial\mathcal{P}_{\eta,q}^{\mathcal{T}}(||\boldsymbol{R}_q\tilde{\boldsymbol{\theta}}||_1)}{\partial||\boldsymbol{R}_q\tilde{\boldsymbol{\theta}}||_1}\frac{1}{\sqrt{(\boldsymbol{R}_q\tilde{\boldsymbol{\theta}})^T\boldsymbol{R}_q\tilde{\boldsymbol{\theta}}+\bar{c}}}\boldsymbol{R}_q^T\boldsymbol{R}_q\right\}\boldsymbol{\theta} \\
& -\frac{1}{2}\tilde{\boldsymbol{\theta}}^T\left\{\frac{\partial\mathcal{P}_{\eta,q}^{\mathcal{T}}(||\boldsymbol{R}_q\tilde{\boldsymbol{\theta}}||_1)}{\partial||\boldsymbol{R}_q\tilde{\boldsymbol{\theta}}||_1}\frac{1}{\sqrt{(\boldsymbol{R}_q\tilde{\boldsymbol{\theta}})^T\boldsymbol{R}_q\tilde{\boldsymbol{\theta}}+\bar{c}}}\boldsymbol{R}_q^T\boldsymbol{R}_q\right\}\tilde{\boldsymbol{\theta}} \\
= \; & \frac{1}{2}\left[\boldsymbol{\theta}^T\boldsymbol{\mathcal{S}}_{\eta,q}^{\mathcal{T}}(\tilde{\boldsymbol{\theta}})\boldsymbol{\theta} - \tilde{\boldsymbol{\theta}}^T\boldsymbol{\mathcal{S}}_{\eta,q}^{\mathcal{T}}(\tilde{\boldsymbol{\theta}})\tilde{\boldsymbol{\theta}}\right],
\end{aligned}
$$

where $\boldsymbol{\mathcal{S}}_{\eta,q}^{\mathcal{T}}(\tilde{\boldsymbol{\theta}}) = \dfrac{\partial\mathcal{P}_{\eta,q}^{\mathcal{T}}(||\boldsymbol{R}_q\tilde{\boldsymbol{\theta}}||_1)}{\partial||\boldsymbol{R}_q\tilde{\boldsymbol{\theta}}||_1}\dfrac{1}{\sqrt{(\boldsymbol{R}_q\tilde{\boldsymbol{\theta}})^T\boldsymbol{R}_q\tilde{\boldsymbol{\theta}}+\bar{c}}}\boldsymbol{R}_q^T\boldsymbol{R}_q$. Let us denote $\boldsymbol{\mathcal{S}}_{\eta}^{\mathcal{T}}(\tilde{\boldsymbol{\theta}}) = \sum_{q=1}^m \boldsymbol{\mathcal{S}}_{\eta,q}^{\mathcal{T}}(\tilde{\boldsymbol{\theta}})$. Then, equation (B.1) can be rewritten as

$$
\mathcal{P}_{\eta}^{\mathcal{T}}(\boldsymbol{\theta}) \approx \mathcal{P}_{\eta}^{\mathcal{T}}(\tilde{\boldsymbol{\theta}}) + \sum_{q=1}^m \left[\frac{\partial\mathcal{P}_{\eta,q}^{\mathcal{T}}(||\boldsymbol{R}_q\tilde{\boldsymbol{\theta}}||_1)}{\partial||\boldsymbol{R}_q\tilde{\boldsymbol{\theta}}||_1}\right]^T \left[\frac{\partial||\boldsymbol{R}_q\tilde{\boldsymbol{\theta}}||_1}{\partial\boldsymbol{R}_q\tilde{\boldsymbol{\theta}}}\right]^T \left[\frac{\partial\boldsymbol{R}_q\tilde{\boldsymbol{\theta}}}{\partial\tilde{\boldsymbol{\theta}}}\right]^T (\boldsymbol{\theta}-\tilde{\boldsymbol{\theta}})
$$

$$= \mathcal{P}_\eta^{\mathcal{T}}(\tilde{\boldsymbol{\theta}}) + \sum_{q=1}^{m} \frac{1}{2}\Big[\boldsymbol{\theta}^T \boldsymbol{\mathcal{S}}_{\eta,q}^{\mathcal{T}}(\tilde{\boldsymbol{\theta}})\boldsymbol{\theta} - \tilde{\boldsymbol{\theta}}^T \boldsymbol{\mathcal{S}}_{\eta,q}^{\mathcal{T}}(\tilde{\boldsymbol{\theta}})\tilde{\boldsymbol{\theta}}\Big]$$

$$= \mathcal{P}_\eta^{\mathcal{T}}(\tilde{\boldsymbol{\theta}}) + \frac{1}{2}\boldsymbol{\theta}^T \boldsymbol{\mathcal{S}}_{\eta}^{\mathcal{T}}(\tilde{\boldsymbol{\theta}})\boldsymbol{\theta} - \frac{1}{2}\tilde{\boldsymbol{\theta}}^T \boldsymbol{\mathcal{S}}_{\eta}^{\mathcal{T}}(\tilde{\boldsymbol{\theta}})\tilde{\boldsymbol{\theta}}.$$

We can ignore the constant terms that do not depend on $\boldsymbol{\theta}$, namely, $\mathcal{P}_\eta^{\mathcal{T}}(\tilde{\boldsymbol{\theta}})$ and $\frac{1}{2}\tilde{\boldsymbol{\theta}}^T \boldsymbol{\mathcal{S}}_{\eta}^{\mathcal{T}}(\tilde{\boldsymbol{\theta}})\tilde{\boldsymbol{\theta}}$. Then, the differentiable local approximation of the penalty $\mathcal{P}_\eta^{\mathcal{T}}(\boldsymbol{\theta})$ is

$$\mathcal{P}_\eta^{\mathcal{T}}(\boldsymbol{\theta}) \approx \frac{1}{2}\boldsymbol{\theta}^T \left\{ \sum_{q=1}^{m} \frac{\partial \mathcal{P}_{\eta,q}^{\mathcal{T}}(||\boldsymbol{R}_q\tilde{\boldsymbol{\theta}}||_1)}{\partial ||\boldsymbol{R}_q\tilde{\boldsymbol{\theta}}||_1} \frac{1}{\sqrt{(\boldsymbol{R}_q\tilde{\boldsymbol{\theta}})^T \boldsymbol{R}_q\tilde{\boldsymbol{\theta}} + \bar{c}}} \boldsymbol{R}_q^T \boldsymbol{R}_q \right\} \boldsymbol{\theta} = \frac{1}{2}\boldsymbol{\theta}^T \boldsymbol{\mathcal{S}}_{\eta}^{\mathcal{T}}(\tilde{\boldsymbol{\theta}})\boldsymbol{\theta}.$$

The specific forms of $\boldsymbol{\mathcal{S}}_{\eta}^{\mathcal{T}}(\tilde{\boldsymbol{\theta}})$ for lasso, alasso, scad and mcp are derived in Section B.1.3.1.

B.1.3.1 The penalty matrices

Based on the approximation derived in Section B.1.3, the penalty matrix $\boldsymbol{\mathcal{S}}_{\eta}^{\mathcal{T}}(\tilde{\boldsymbol{\theta}})$ is defined as

$$\boldsymbol{\mathcal{S}}_{\eta}^{\mathcal{T}}(\tilde{\boldsymbol{\theta}}) = \sum_{q=1}^{m} \frac{\partial \mathcal{P}_{\eta,q}^{\mathcal{T}}(||\boldsymbol{R}_q\tilde{\boldsymbol{\theta}}||_1)}{\partial ||\boldsymbol{R}_q\tilde{\boldsymbol{\theta}}||_1} \frac{1}{\sqrt{(\boldsymbol{R}_q\tilde{\boldsymbol{\theta}})^T \boldsymbol{R}_q\tilde{\boldsymbol{\theta}} + \bar{c}}} \boldsymbol{R}_q^T \boldsymbol{R}_q,$$

for $\mathcal{T} = \{L, A, S, M\}$. Recall that $\boldsymbol{R}_q = \text{diag}(0, 0, \ldots, 0, 1, 0, \ldots, 0)$ for $q = 1, \ldots, q^\star$ where the 1 on the $(q, q)^{\text{th}}$ entry of the matrix corresponds to the qth parameter in $\boldsymbol{\theta}$, and $\boldsymbol{R}_q = \boldsymbol{O}_{m\times m}$ for $q = q^\star + 1, \ldots, m$. Therefore, the penalty matrix $\boldsymbol{\mathcal{S}}_{\eta}^{\mathcal{T}}(\tilde{\boldsymbol{\theta}})$ is an $m \times m$ block diagonal matrix of the form:

$$\boldsymbol{\mathcal{S}}_{\eta}^{\mathcal{T}}(\tilde{\boldsymbol{\theta}}) = \begin{bmatrix} \boldsymbol{\mathcal{M}}_{\eta}^{\mathcal{T}}(\tilde{\boldsymbol{\theta}}) & \boldsymbol{O} \\ \boldsymbol{O} & \boldsymbol{O} \end{bmatrix}.$$

The first block is composed of the $q^\star \times q^\star$ diagonal matrix $\boldsymbol{\mathcal{M}}_{\eta}^{\mathcal{T}}(\tilde{\boldsymbol{\theta}})$ and corresponds to the penalized parameters (i.e., the $q^\star$ factor loadings), whereas the second block is an $(m - q^\star)$-dimensional null matrix relative to the unpenalized parameters (i.e., the factor variances and covariances and the unique variances). The matrix $\boldsymbol{\mathcal{M}}_{\eta}^{\mathcal{T}}(\tilde{\boldsymbol{\theta}})$ has the following structure

$$\boldsymbol{\mathcal{M}}_{\eta}^{\mathcal{T}}(\tilde{\boldsymbol{\theta}}) = \begin{bmatrix} m_1^{\mathcal{T}} & \dots & 0 & \dots & 0 \\ \vdots & \ddots & & & \vdots \\ 0 & \dots & m_q^{\mathcal{T}} & \dots & 0 \\ \vdots & & & \ddots & \vdots \\ 0 & \dots & 0 & \dots & m_{q^\star}^{\mathcal{T}} \end{bmatrix},$$

where the diagonal entries $m_q^{\mathcal{T}} = \dfrac{\partial \mathcal{P}_{\eta,q}^{\mathcal{T}}(||\boldsymbol{R}_q\tilde{\boldsymbol{\theta}}||_1)}{\partial ||\boldsymbol{R}_q\tilde{\boldsymbol{\theta}}||_1} \dfrac{1}{\sqrt{(\boldsymbol{R}_q\tilde{\boldsymbol{\theta}})^T \boldsymbol{R}_q\tilde{\boldsymbol{\theta}} + \bar{c}}}$ (for $q = 1, \dots, q^\star$) determine the amount of shrinkage on $\tilde{\theta}_q$ controlled by the tuning η and required by penalty $\mathcal{T}$. We now derive their expressions for the lasso, alasso, scad and mcp.

Lasso

The derivative of the lasso penalty with respect to the L_1-norm of its argument is simply the tuning parameter, that is,

$$\frac{\partial \mathcal{P}_{\eta,q}^{L}(||\boldsymbol{R}_q\tilde{\boldsymbol{\theta}}||_1)}{\partial ||\boldsymbol{R}_q\tilde{\boldsymbol{\theta}}||_1} = \frac{\partial \left(\eta ||\boldsymbol{R}_q\tilde{\boldsymbol{\theta}}||_1\right)}{\partial ||\boldsymbol{R}_q\tilde{\boldsymbol{\theta}}||_1} = \eta.$$

Therefore,

$$\left[\boldsymbol{\mathcal{M}}_{\eta}^{L}(\tilde{\boldsymbol{\theta}})\right]_{qq} = m_q^L \quad = \quad \frac{\partial \mathcal{P}_{\eta,q}^{L}(||\boldsymbol{R}_q\tilde{\boldsymbol{\theta}}||_1)}{\partial ||\boldsymbol{R}_q\tilde{\boldsymbol{\theta}}||_1} \frac{1}{\sqrt{(\boldsymbol{R}_q\tilde{\boldsymbol{\theta}})^T \boldsymbol{R}_q\tilde{\boldsymbol{\theta}} + \bar{c}}} = \frac{\eta}{\sqrt{(\boldsymbol{R}_q\tilde{\boldsymbol{\theta}})^T \boldsymbol{R}_q\tilde{\boldsymbol{\theta}} + \bar{c}}} = \frac{\eta}{\sqrt{\tilde{\theta}_q^2 + \bar{c}}}.$$

Alasso

Similarly, the derivative of the alasso penalty with respect to the L_1-norm of its argument is the tuning parameter multiplied by the adaptive weight, that is,

$$\frac{\partial \mathcal{P}_{\eta,q}^{A}(||\boldsymbol{R}_q\tilde{\boldsymbol{\theta}}||_1)}{\partial ||\boldsymbol{R}_q\tilde{\boldsymbol{\theta}}||_1} \quad = \quad \frac{\partial}{\partial ||\boldsymbol{R}_q\tilde{\boldsymbol{\theta}}||_1} \left(\eta \frac{||\boldsymbol{R}_q\tilde{\boldsymbol{\theta}}||_1}{\left|\left|\boldsymbol{R}_q\hat{\boldsymbol{\theta}}\right|\right|_1^a} \right) = \eta \frac{1}{\left|\left|\boldsymbol{R}_q\hat{\boldsymbol{\theta}}\right|\right|_1^a} = \eta \frac{1}{|\hat{\theta}_q|^a} = \eta\, w_q.$$

Therefore,

$$\left[\boldsymbol{\mathcal{M}}_{\eta}^{A}(\tilde{\boldsymbol{\theta}})\right]_{qq} = m_q^A \quad = \quad \frac{\partial \mathcal{P}_{\eta,q}^{A}(||\boldsymbol{R}_q\tilde{\boldsymbol{\theta}}||_1)}{\partial ||\boldsymbol{R}_q\tilde{\boldsymbol{\theta}}||_1} \frac{1}{\sqrt{(\boldsymbol{R}_q\tilde{\boldsymbol{\theta}})^T \boldsymbol{R}_q\tilde{\boldsymbol{\theta}} + \bar{c}}} = \eta\, w_q \frac{1}{\sqrt{(\boldsymbol{R}_q\tilde{\boldsymbol{\theta}})^T \boldsymbol{R}_q\tilde{\boldsymbol{\theta}} + \bar{c}}} = \frac{\eta}{|\hat{\theta}_q|^a \sqrt{\tilde{\theta}_q^2 + \bar{c}}},$$

where $\hat{\boldsymbol{\theta}}$ is generally the maximum likelihood estimator $\hat{\boldsymbol{\theta}}^{\text{MLE}}$.

Scad

The derivative of the scad penalty with respect to the L_1-norm of its argument has the form:

$$\begin{aligned}\frac{\partial \mathcal{P}^S_{\eta,q}(||\boldsymbol{R}_q\tilde{\boldsymbol{\theta}}||_1)}{\partial ||\boldsymbol{R}_q\tilde{\boldsymbol{\theta}}||_1} &= \eta\left\{\mathbb{1}(||\boldsymbol{R}_q\tilde{\boldsymbol{\theta}}||_1 \leq \eta) + \frac{\max(a\eta - ||\boldsymbol{R}_q\tilde{\boldsymbol{\theta}}||_1, 0)}{(a-1)\eta}\mathbb{1}(||\boldsymbol{R}_q\tilde{\boldsymbol{\theta}}||_1 > \eta)\right\} \\ &= \begin{cases}\eta & \text{if } |\tilde{\theta}_q| \leq \eta, \\ \dfrac{\max(a\eta - |\tilde{\theta}_q|, 0)}{a-1} & \text{if } |\tilde{\theta}_q| > \eta,\end{cases}\end{aligned}$$

which leads to the following expression

$$\begin{aligned}\left[\boldsymbol{\mathcal{M}}^S_\eta(\tilde{\boldsymbol{\theta}})\right]_{qq} = m^S_q &= \frac{\partial \mathcal{P}^S_{\eta,q}(||\boldsymbol{R}_q\tilde{\boldsymbol{\theta}}||_1)}{\partial ||\boldsymbol{R}_q\tilde{\boldsymbol{\theta}}||_1}\frac{1}{\sqrt{(\boldsymbol{R}_q\tilde{\boldsymbol{\theta}})^T\boldsymbol{R}_q\tilde{\boldsymbol{\theta}} + \bar{c}}} \\ &= \eta\left\{\mathbb{1}(||\boldsymbol{R}_q\tilde{\boldsymbol{\theta}}||_1 \leq \eta) + \frac{\max(a\eta - ||\boldsymbol{R}_q\tilde{\boldsymbol{\theta}}||_1, 0)}{(a-1)\eta}\mathbb{1}(||\boldsymbol{R}_q\tilde{\boldsymbol{\theta}}||_1 > \eta)\right\}\frac{1}{\sqrt{(\boldsymbol{R}_q\tilde{\boldsymbol{\theta}})^T\boldsymbol{R}_q\tilde{\boldsymbol{\theta}} + \bar{c}}} \\ &= \frac{\eta\left[\mathbb{1}(|\tilde{\theta}_q| \leq \eta) + \dfrac{\max(a\eta - |\tilde{\theta}_q|, 0)}{(a-1)\eta}\mathbb{1}(|\tilde{\theta}_q| > \eta)\right]}{\sqrt{\tilde{\theta}_q^2 + \bar{c}}}.\end{aligned}$$

Mcp

The derivative of the mcp penalty with respect to the L_1-norm of its argument is

$$\frac{\partial \mathcal{P}^M_{\eta,q}(||\boldsymbol{R}_q\tilde{\boldsymbol{\theta}}||_1)}{\partial ||\boldsymbol{R}_q\tilde{\boldsymbol{\theta}}||_1} = \left(\eta - \frac{||\boldsymbol{R}_q\tilde{\boldsymbol{\theta}}||_1}{a}\right)\mathbb{1}(||\boldsymbol{R}_q\tilde{\boldsymbol{\theta}}||_1 < \eta a) = \begin{cases}\eta - \dfrac{|\tilde{\theta}_q|}{a} & \text{if } |\tilde{\theta}_q| \leq \eta a, \\ 0 & \text{if } |\tilde{\theta}_q| > \eta a,\end{cases}$$

which implies that

$$\begin{aligned}\left[\boldsymbol{\mathcal{M}}^M_\eta(\tilde{\boldsymbol{\theta}})\right]_{qq} = m^M_q &= \frac{\partial \mathcal{P}^M_{\eta,q}(||\boldsymbol{R}_q\tilde{\boldsymbol{\theta}}||_1)}{\partial ||\boldsymbol{R}_q\tilde{\boldsymbol{\theta}}||_1}\frac{1}{\sqrt{(\boldsymbol{R}_q\tilde{\boldsymbol{\theta}})^T\boldsymbol{R}_q\tilde{\boldsymbol{\theta}} + \bar{c}}} \\ &= \left(\eta - \frac{||\boldsymbol{R}_q\tilde{\boldsymbol{\theta}}||_1}{a}\right)\mathbb{1}(||\boldsymbol{R}_q\tilde{\boldsymbol{\theta}}||_1 < \eta a)\frac{1}{\sqrt{(\boldsymbol{R}_q\tilde{\boldsymbol{\theta}})^T\boldsymbol{R}_q\tilde{\boldsymbol{\theta}} + \bar{c}}} = \frac{\left(\eta - \dfrac{|\tilde{\theta}_q|}{a}\right)\mathbb{1}(|\tilde{\theta}_q| < \eta a)}{\sqrt{\tilde{\theta}_q^2 + \bar{c}}}.\end{aligned}$$

Notice that the penalty $\mathcal{P}^{\mathcal{T}}_\eta(\boldsymbol{\theta})$ for $\mathcal{T} = L, A, S, M$, is approximated as $\frac{1}{2}\boldsymbol{\theta}^T\boldsymbol{\mathcal{S}}^{\mathcal{T}}_\eta(\tilde{\boldsymbol{\theta}})\boldsymbol{\theta}$, where $\boldsymbol{\mathcal{S}}^{\mathcal{T}}_\eta(\tilde{\boldsymbol{\theta}})$ is the matrix in (8) and the form of $\boldsymbol{\mathcal{M}}^{\mathcal{T}}_\eta(\tilde{\boldsymbol{\theta}})$ changes according to the penalty function (see

equations (9)-(12)). The penalty $\mathcal{P}_\eta^{\mathcal{T}}(\boldsymbol{\theta})$ is not approximated by replacing the absolute value $|\theta_q|$ appearing in equations (3)-(6) with the approximation $(\theta_q^2 + \bar{c})^{\frac{1}{2}}$ proposed by Koch (1996).

B.2 Multiple-group factor model

The rationale used to approximate the sparsity-inducing penalties in the normal linear factor model is extended to the case of multiple groups to find differentiable local approximations of the invariance-inducing penalties described in Section 5. An example clarifying the formulation of the combined penalty is provided in Section B.2.2.

B.2.1 Differentiable approximations

The free parameters of the factor model of each group appearing in vec$(\boldsymbol{\Lambda}_g)$, $\boldsymbol{\tau}_g$, diag$(\boldsymbol{\Psi}_g)$, vech$(\boldsymbol{\Phi}_g)$, and $\boldsymbol{\kappa}_g$ are collected in the m_g-dimensional vector $\boldsymbol{\theta}_g$, for $g = 1, \ldots, G$, where the vec$(\cdot)$ operator converts the enclosed matrix into a vector by stacking its columns, diag$(\cdot)$ extracts the diagonal elements of the enclosed symmetric matrix, and vech$(\cdot)$ vectorizes the lower-diagonal part of the enclosed symmetric matrix. Without loss of generality, assume that the number of observed variables p and common factors r is the same across groups, and that the fixed elements required for model identification are placed in the same positions across groups. Denote the number of factor loadings in each group (i.e., the free elements in vec$(\boldsymbol{\Lambda}_g)$) as $q^\star$, and the number of intercepts in each group (i.e., the free elements in $\boldsymbol{\tau}_g$) as $k^\star$. Because of the presence of fixed elements in $\boldsymbol{\Lambda}_g$ and $\boldsymbol{\tau}_g$ for model identification, $q^\star$ is smaller than $p \times r$, and $k^\star$ is smaller than p. Each group parameter vector is collected in the overall m-dimensional vector $\boldsymbol{\theta} = (\boldsymbol{\theta}_1^T, \ldots, \boldsymbol{\theta}_g^T, \ldots, \boldsymbol{\theta}_G^T)^T$, where $m = \sum_{g=1}^G m_g$. Assume for convenience that each parameter sub-vector has the same dimension, i.e., $m_1 = \ldots = m_G$, so that $m = m_1 G$.

We now describe the structure of the penalty inducing equal factor loadings across groups. The penalty inducing equal intercepts across groups has precisely the same structure, the only difference being in the type of parameters among which the differences are computed. A penalty function that encourages invariant factor loadings can be conveniently specified in terms of a penalty function shrinking the pairwise absolute differences of every factor loading across groups.

Let $\boldsymbol{D}_q^{\boldsymbol{\Lambda}}$, for $q = 1, \ldots, q^\star$, be the matrix computing the differences of the factor loading pairs

$(\theta_{(g-1)m_1+q}, \theta_{(g'-1)m_1+q})$ for $g < g'$. It has dimension equal to $m_1\binom{G}{2} \times m$, where the binomial coefficient $\binom{G}{2}$ denotes the total number of pairwise group differences for a given factor loading. In its general form, $\boldsymbol{D}_q^{\boldsymbol{\Lambda}}$ is a matrix with zeros in every position, except the $((s-1)m_1+q, (g-1)m_1+q)$ entries, which contain a 1.0, and the entries $((s-1)m_1+q, (g'-1)m_1+q)$, which contain a -1.0, for $s = 1, \dots, G$ and $g < g'$ (see Matrix $\boldsymbol{D}_q^{\boldsymbol{\Lambda}}$). For the other parameters (i.e., the intercepts, the unique variances and the structural parameters), $\boldsymbol{D}_q^{\boldsymbol{\Lambda}} = \boldsymbol{O}_{m_1\binom{G}{2}\times m}$. Notice that $||\boldsymbol{D}_q^{\boldsymbol{\Lambda}}\boldsymbol{\theta}||_1 = \sum_{g<g'}|\theta_{(g-1)m_1+q} - \theta_{(g'-1)m_1+q}|$ for $q = 1, \dots, q^\star$, and is equal to zero otherwise. Then, the penalty inducing equal loadings across groups can be written as $\mathcal{P}_{\eta_2}^{\mathcal{T}}(\boldsymbol{\theta}) = \sum_{q=1}^{m} \mathcal{P}_{\eta_2,q}^{\mathcal{T}}(||\boldsymbol{D}_q^{\boldsymbol{\Lambda}}\boldsymbol{\theta}||_1)$.

The derivation of the expression of the penalty $\mathcal{P}_{\eta_2}^{\mathcal{T}}(\boldsymbol{\theta})$ shrinking the pairwise group differences of the factor loadings follows the same rationale described in Section B.1.2, with the only difference being that $\boldsymbol{R}_q\boldsymbol{\theta}$ is now replaced by $\boldsymbol{D}_q^{\boldsymbol{\Lambda}}\boldsymbol{\theta}$. The forms of the lasso, alasso, scad, and mcp penalties for the differences are:

$$
\begin{aligned}
\mathcal{P}_{\eta_2}^{L}(\boldsymbol{\theta}) &= \eta_2 \sum_{g<g'} \sum_{q=1}^{q^\star} |\theta_{(g-1)m_1+q} - \theta_{(g'-1)m_1+q}|, \\
\mathcal{P}_{\eta_2}^{A}(\boldsymbol{\theta}) &= \eta_2 \sum_{g<g'} \sum_{q=1}^{q^\star} \frac{|\theta_{(g-1)m_1+q} - \theta_{(g'-1)m_1+q}|}{\left|\hat{\theta}_{(g-1)m_1+q} - \hat{\theta}_{(g'-1)m_1+q}\right|^{a}}, \\
\mathcal{P}_{\eta_2}^{S}(\boldsymbol{\theta}) &= \sum_{g<g'} \sum_{q=1}^{q^\star} \Bigg\{ \eta_2|\theta_{(g-1)m_1+q} - \theta_{(g'-1)m_1+q}| \mathbb{1}(0 \leq |\theta_{(g-1)m_1+q} - \theta_{(g'-1)m_1+q}| \leq \eta_2) \\
&\qquad - \left[\frac{(\theta_{(g-1)m_1+q} - \theta_{(g'-1)m_1+q})^2 + \eta_2^2 - 2\eta_2 a|\theta_{(g-1)m_1+q} - \theta_{(g'-1)m_1+q}|}{2(a-1)} \right] \\
&\qquad \times \mathbb{1}(\eta_2 < |\theta_{(g-1)m_1+q} - \theta_{(g'-1)m_1+q}| \leq a\eta_2) \\
&\qquad + \frac{\eta_2^2(a+1)}{2} \left(|\theta_{(g-1)m_1+q} - \theta_{(g'-1)m_1+q}| > a\eta_2 \right) \Bigg\}, \\
\mathcal{P}_{\eta_2}^{M}(\boldsymbol{\theta}) &= \sum_{g<g'} \sum_{q=1}^{q^\star} \Bigg\{ \left(\eta_2|\theta_{(g-1)m_1+q} - \theta_{(g'-1)m_1+q}| - \frac{(\theta_{(g-1)m_1+q} - \theta_{(g'-1)m_1+q})^2}{2a} \right) \\
&\qquad \times \mathbb{1}(0 \leq |\theta_{(g-1)m_1+q} - \theta_{(g'-1)m_1+q}| \leq a\eta_2) \\
&\qquad + \frac{\eta_2^2 a}{2} \mathbb{1}(|\theta_{(g-1)m_1+q} - \theta_{(g'-1)m_1+q}| > a\eta_2) \Bigg\},
\end{aligned}
$$

where for the alasso $a > 0$, for the scad $a > 2$ and for the mcp $a > 1$.

$$
\begin{array}{ccccc}
m_1 & 2m_1 & (G-2)m_1 & (G-1)m_1 & Gm_1
\end{array}
$$

$$
\boldsymbol{D}_q^{\boldsymbol{\Lambda}} = \left[
\begin{array}{ccccc:ccccc:cc:ccccc:ccccc}
0 & \cdots & 0 & \cdots & 0 & 0 & \cdots & 0 & \cdots & 0 & \cdots & \cdots & 0 & \cdots & 0 & \cdots & 0 & 0 & \cdots & 0 & \cdots & 0 \\
\vdots & \ddots & & & \vdots & \vdots & \ddots & & & \vdots & \cdots & \cdots & \vdots & \ddots & & & \vdots & \vdots & \ddots & & & \vdots \\
0 & \cdots & 1 & \cdots & 0 & 0 & \cdots & -1 & \cdots & 0 & \cdots & \cdots & 0 & \cdots & 0 & \cdots & 0 & 0 & \cdots & 0 & \cdots & 0 \\
\vdots & & & \ddots & \vdots & \vdots & & & \ddots & \vdots & \cdots & \cdots & \vdots & & & \ddots & \vdots & \vdots & & & \ddots & \vdots \\
0 & \cdots & 0 & \cdots & 0 & 0 & \cdots & 0 & \cdots & 0 & \cdots & \cdots & 0 & \cdots & 0 & \cdots & 0 & 0 & \cdots & 0 & \cdots & 0 \\
\hdashline
\vdots & & \vdots & & \vdots & \vdots & & \vdots & & \vdots & \vdots & \vdots & \vdots & & \vdots & & \vdots & \vdots & & \vdots & & \vdots \\
\vdots & & \vdots & & \vdots & \vdots & & \vdots & & \vdots & \vdots & \vdots & \vdots & & \vdots & & \vdots & \vdots & & \vdots & & \vdots \\
\hdashline
0 & \cdots & 0 & \cdots & 0 & 0 & \cdots & 0 & \cdots & 0 & \cdots & \cdots & 0 & \cdots & 0 & \cdots & 0 & 0 & \cdots & 0 & \cdots & 0 \\
\vdots & \ddots & & & \vdots & \vdots & \ddots & & & \vdots & \cdots & \cdots & \vdots & \ddots & & & \vdots & \vdots & \ddots & & & \vdots \\
0 & \cdots & 1 & \cdots & 0 & 0 & \cdots & 0 & \cdots & 0 & \cdots & \cdots & 0 & \cdots & 0 & \cdots & 0 & 0 & \cdots & -1 & \cdots & 0 \\
\vdots & & & \ddots & \vdots & \vdots & & & \ddots & \vdots & \cdots & \cdots & \vdots & & & \ddots & \vdots & \vdots & & & \ddots & \vdots \\
0 & \cdots & 0 & \cdots & 0 & 0 & \cdots & 0 & \cdots & 0 & \cdots & \cdots & 0 & \cdots & 0 & \cdots & 0 & 0 & \cdots & 0 & \cdots & 0 \\
\hdashline
\vdots & & \vdots & & \vdots & \vdots & & \vdots & & \vdots & \vdots & \vdots & \vdots & & \vdots & & \vdots & \vdots & & \vdots & & \vdots \\
\vdots & & \vdots & & \vdots & \vdots & & \vdots & & \vdots & \vdots & \vdots & \vdots & & \vdots & & \vdots & \vdots & & \vdots & & \vdots \\
\hdashline
0 & \cdots & 0 & \cdots & 0 & 0 & \cdots & 0 & \cdots & 0 & \cdots & \cdots & 0 & \cdots & 0 & \cdots & 0 & 0 & \cdots & 0 & \cdots & 0 \\
\vdots & \ddots & & & \vdots & \vdots & \ddots & & & \vdots & \cdots & \cdots & \vdots & \ddots & & & \vdots & \vdots & \ddots & & & \vdots \\
0 & \cdots & 0 & \cdots & 0 & 0 & \cdots & 1 & \cdots & 0 & \cdots & \cdots & 0 & \cdots & 0 & \cdots & 0 & 0 & \cdots & -1 & \cdots & 0 \\
\vdots & & & \ddots & \vdots & \vdots & & & \ddots & \vdots & \cdots & \cdots & \vdots & & & \ddots & \vdots & \vdots & & & \ddots & \vdots \\
0 & \cdots & 0 & \cdots & 0 & 0 & \cdots & 0 & \cdots & 0 & \cdots & \cdots & 0 & \cdots & 0 & \cdots & 0 & 0 & \cdots & 0 & \cdots & 0 \\
\hdashline
\vdots & & \vdots & & \vdots & \vdots & & \vdots & & \vdots & \vdots & \vdots & \vdots & & \vdots & & \vdots & \vdots & & \vdots & & \vdots \\
\vdots & & \vdots & & \vdots & \vdots & & \vdots & & \vdots & \vdots & \vdots & \vdots & & \vdots & & \vdots & \vdots & & \vdots & & \vdots \\
\hdashline
0 & \cdots & 0 & \cdots & 0 & 0 & \cdots & 0 & \cdots & 0 & \cdots & \cdots & 0 & \cdots & 0 & \cdots & 0 & 0 & \cdots & 0 & \cdots & 0 \\
\vdots & \ddots & & & \vdots & \vdots & \ddots & & & \vdots & \cdots & \cdots & \vdots & \ddots & & & \vdots & \vdots & \ddots & & & \vdots \\
0 & \cdots & 0 & \cdots & 0 & 0 & \cdots & 0 & \cdots & 0 & \cdots & \cdots & 0 & \cdots & 1 & \cdots & 0 & 0 & \cdots & -1 & \cdots & 0 \\
\vdots & & & \ddots & \vdots & \vdots & & & \ddots & \vdots & \cdots & \cdots & \vdots & & & \ddots & \vdots & \vdots & & & \ddots & \vdots \\
0 & \cdots & 0 & \cdots & 0 & 0 & \cdots & 0 & \cdots & 0 & \cdots & \cdots & 0 & \cdots & 0 & \cdots & 0 & 0 & \cdots & 0 & \cdots & 0
\end{array}
\right]
\begin{array}{l}
\left.\vphantom{\begin{array}{c}0\\0\\0\\0\\0\end{array}}\right\} m_1 \\
\left.\vphantom{\begin{array}{c}0\\0\end{array}}\right\} m_1(G-3) \\
\left.\vphantom{\begin{array}{c}0\\0\\0\\0\\0\end{array}}\right\} m_1 \\
\left.\vphantom{\begin{array}{c}0\\0\end{array}}\right\} m_1(G-3) \\
\left.\vphantom{\begin{array}{c}0\\0\\0\\0\\0\end{array}}\right\} m_1 \\
\left.\vphantom{\begin{array}{c}0\\0\end{array}}\right\} \frac{(G-3)(G-2)}{2} - 1 \\
\left.\vphantom{\begin{array}{c}0\\0\\0\\0\\0\end{array}}\right\} m_1
\end{array}
$$

Matrix $\boldsymbol{D}_q^{\boldsymbol{\Lambda}}$: The general structure of the matrix $\boldsymbol{D}_q^{\boldsymbol{\Lambda}}$ computing the pairwise differences of the qth factor loading across G groups.

Given that the penalty term $\mathcal{P}^{\mathcal{T}}_{\eta_2}(\boldsymbol{\theta})$ is non-differentiable at $\boldsymbol{\theta} = \mathbf{0}$, we replace it with its differentiable local approximation. Following the same reasoning described in Section B.1.3, the locally approximated penalty takes the form

$$\mathcal{P}^{\mathcal{T}}_{\eta_2}(\boldsymbol{\theta}) \approx \frac{1}{2}\boldsymbol{\theta}^T \left\{ \sum_{q=1}^{m} \frac{\partial \mathcal{P}^{\mathcal{T}}_{\eta_2,q}(||\boldsymbol{D}^{\boldsymbol{\Lambda}}_q \tilde{\boldsymbol{\theta}}||_1)}{\partial ||\boldsymbol{D}^{\boldsymbol{\Lambda}}_q \tilde{\boldsymbol{\theta}}||_1} \frac{1}{\sqrt{(\boldsymbol{D}^{\boldsymbol{\Lambda}}_q \tilde{\boldsymbol{\theta}})^T \boldsymbol{D}^{\boldsymbol{\Lambda}}_q \tilde{\boldsymbol{\theta}} + \bar{c}}} \boldsymbol{D}^{\boldsymbol{\Lambda}^T}_q \boldsymbol{D}^{\boldsymbol{\Lambda}}_q \right\} \boldsymbol{\theta} = \frac{1}{2}\boldsymbol{\theta}^T \boldsymbol{\mathcal{D}}^{\mathcal{T}}_{\eta_2}(\tilde{\boldsymbol{\theta}})\boldsymbol{\theta}.$$

Let $d^{\mathcal{T}}_q = \dfrac{\partial \mathcal{P}^{\mathcal{T}}_{\eta_2,q}(||\boldsymbol{D}^{\boldsymbol{\Lambda}}_q \tilde{\boldsymbol{\theta}}||_1)}{\partial ||\boldsymbol{D}^{\boldsymbol{\Lambda}}_q \tilde{\boldsymbol{\theta}}||_1} \dfrac{1}{\sqrt{(\boldsymbol{D}^{\boldsymbol{\Lambda}}_q \tilde{\boldsymbol{\theta}})^T \boldsymbol{D}^{\boldsymbol{\Lambda}}_q \tilde{\boldsymbol{\theta}} + \bar{c}}}$. If $\boldsymbol{D}^{\boldsymbol{\Lambda}}_q$ for the parameter θ_q is non-null, the expressions of $d^{\mathcal{T}}_q$ for the lasso, alasso, scad and mcp penalties are:

$$d^L_q = \frac{\eta_2}{\sqrt{\sum_{g<g'}(\tilde{\theta}_{(g-1)m_1+q} - \tilde{\theta}_{(g'-1)m_1+q})^2 + \bar{c}}},$$

$$d^A_q = \frac{\eta_2}{\left\{\sum_{g<g'}|\hat{\theta}_{(g-1)m_1+q} - \hat{\theta}_{(g'-1)m_1+q}|\right\}^a \sqrt{\sum_{g<g'}(\tilde{\theta}_{(g-1)m_1+q} - \tilde{\theta}_{(g'-1)m_1+q})^2 + \bar{c}}},$$

$$d^S_q = \begin{cases} \dfrac{\eta_2}{\sqrt{\sum_{g<g'}(\tilde{\theta}_{(g-1)m_1+q} - \tilde{\theta}_{(g'-1)m_1+q})^2 + \bar{c}}} & \text{if } \sum_{g<g'}|\tilde{\theta}_{(g-1)m_1+q} - \tilde{\theta}_{(g'-1)m_1+q}| \leq \eta_2, \\[2ex] \dfrac{\dfrac{\max(a\eta_2 - \sum_{g<g'}|\tilde{\theta}_{(g-1)m_1+q} - \tilde{\theta}_{(g'-1)m_1+q}|, 0)}{a-1}}{\sqrt{\sum_{g<g'}(\tilde{\theta}_{(g-1)m_1+q} - \tilde{\theta}_{(g'-1)m_1+q})^2 + \bar{c}}} & \text{if } \sum_{g<g'}|\tilde{\theta}_{(g-1)m_1+q} - \tilde{\theta}_{(g'-1)m_1+q}| > \eta_2, \end{cases}$$

$$d^M_q = \begin{cases} \dfrac{\eta_2 - \dfrac{\sum_{g<g'}|\tilde{\theta}_{(g-1)m_1+q} - \tilde{\theta}_{(g'-1)m_1+q}|}{a}}{\sqrt{\sum_{g<g'}(\tilde{\theta}_{(g-1)m_1+q} - \tilde{\theta}_{(g'-1)m_1+q})^2 + \bar{c}}} & \text{if } \sum_{g<g'}|\tilde{\theta}_{(g-1)m_1+q} - \tilde{\theta}_{(g'-1)m_1+q}| \leq \eta_2 a, \\[2ex] 0 & \text{if } \sum_{g<g'}|\tilde{\theta}_{(g-1)m_1+q} - \tilde{\theta}_{(g'-1)m_1+q}| > \eta_2 a, \end{cases}$$

where for the alasso $a > 0$, for the scad $a > 2$ and for the mcp $a > 1$. The specification of the matrix $\boldsymbol{D}^{\boldsymbol{\tau}}_q$ computing the pairwise differences of the intercepts across groups and the corresponding expression of the approximated penalty matrix $\boldsymbol{\mathcal{D}}^{\mathcal{T}}_{\eta_3}(\tilde{\boldsymbol{\theta}})$ follows the same rationale described for $\boldsymbol{D}^{\boldsymbol{\Lambda}}_q$ and $\boldsymbol{\mathcal{D}}^{\mathcal{T}}_{\eta_2}(\tilde{\boldsymbol{\theta}})$.

B.2.2 Example

Consider the following two-group factor model with $p = 6$ observed variables and $r = 2$ factors:

$$\boldsymbol{x}_g = \boldsymbol{\tau}_g + \boldsymbol{\Lambda}_g \boldsymbol{f}_g + \boldsymbol{\varepsilon}_g \qquad \text{for } g = 1, 2,$$

where $\boldsymbol{f}_g \sim \mathcal{N}(\boldsymbol{\kappa}_g, \boldsymbol{\Phi}_g)$, $\boldsymbol{\varepsilon}_g \sim \mathcal{N}(\mathbf{0}, \boldsymbol{\Psi}_g)$, with $\boldsymbol{\Psi}_g$ a diagonal matrix, and $\boldsymbol{f}_g$ is independent of $\boldsymbol{\varepsilon}_g$. The parameter matrices are as follows, for $g = 1, 2$:

$$\boldsymbol{\Lambda}_g = \begin{bmatrix} \underline{\mathit{1}} & \underline{\mathit{0}} \\ \lambda_{21g} & \lambda_{22g} \\ \lambda_{31g} & \lambda_{32g} \\ \underline{\mathit{0}} & \underline{\mathit{1}} \\ \lambda_{51g} & \lambda_{52g} \\ \lambda_{61g} & \lambda_{62g} \end{bmatrix} \qquad \boldsymbol{\tau}_g = \begin{bmatrix} \underline{\mathit{0}} \\ \tau_{2g} \\ \tau_{3g} \\ \underline{\mathit{0}} \\ \tau_{5g} \\ \tau_{6g} \end{bmatrix} \qquad \boldsymbol{\Psi}_g = \begin{bmatrix} \psi_{11g} & 0 & 0 & 0 & 0 & 0 \\ & \psi_{22g} & 0 & 0 & 0 & 0 \\ & & \psi_{33g} & 0 & 0 & 0 \\ & & & \psi_{44g} & 0 & 0 \\ & & & & \psi_{55g} & 0 \\ & & & & & \psi_{66g} \end{bmatrix},$$

$$\boldsymbol{\Phi}_g = \begin{bmatrix} \phi_{11g} & \phi_{12g} \\ & \phi_{22g} \end{bmatrix} \qquad \boldsymbol{\kappa}_g = \begin{bmatrix} \kappa_{1g} \\ \kappa_{2g} \end{bmatrix}.$$

The factor loadings and intercepts of variables x_1 and x_4 have been fixed for metric setting and identification purposes. The parameters of each group appearing in vec($\boldsymbol{\Lambda}_g$), $\boldsymbol{\tau}_g$, diag($\boldsymbol{\Psi}_g$), vech($\boldsymbol{\Phi}_g$), and $\boldsymbol{\kappa}_g$ are collected in the m_g-dimensional vectors:

$$\begin{aligned} \boldsymbol{\theta}_1 &= (\lambda_{211}, \lambda_{311}, \lambda_{511}, \lambda_{611}, \lambda_{221}, \lambda_{321}, \lambda_{521}, \lambda_{621}, \tau_{21}, \tau_{31}, \tau_{51}, \tau_{61}, \psi_{111}, \psi_{221}, \psi_{331}, \\ &\quad \psi_{441}, \psi_{551}, \psi_{661}, \phi_{111}, \phi_{121}, \phi_{221}, \kappa_{11}, \kappa_{21})^T, \\ \boldsymbol{\theta}_2 &= (\lambda_{212}, \lambda_{312}, \lambda_{512}, \lambda_{612}, \lambda_{222}, \lambda_{322}, \lambda_{522}, \lambda_{622}, \tau_{22}, \tau_{32}, \tau_{52}, \tau_{62}, \psi_{112}, \psi_{222}, \psi_{332}, \\ &\quad \psi_{442}, \psi_{552}, \psi_{662}, \phi_{112}, \phi_{122}, \phi_{222}, \kappa_{12}, \kappa_{22})^T, \end{aligned}$$

where $m_1 = m_2 = 23$. The two group parameter vectors are combined into the m-dimensional vector $\boldsymbol{\theta} = (\boldsymbol{\theta}_1^T, \boldsymbol{\theta}_2^T)^T$, which can be conveniently expressed as

$$\boldsymbol{\theta} = (\underbrace{\theta_1, \theta_2, \theta_3, \theta_4, \theta_5, \theta_6, \theta_7, \theta_8}_{\substack{\textit{Factor loadings}\\ \textit{of Group 1}}}, \underbrace{\theta_9, \theta_{10}, \theta_{11}, \theta_{12}}_{\substack{\textit{Intercepts}\\ \textit{of Group 1}}}, \theta_{13}, \theta_{14}, \theta_{15}, \theta_{16}, \theta_{17}, \theta_{18}, \theta_{19}, \theta_{20},$$
$$\theta_{21}, \theta_{22}, \theta_{23}, \underbrace{\theta_{24}, \theta_{25}, \theta_{26}, \theta_{27}, \theta_{28}, \theta_{29}, \theta_{30}, \theta_{31}}_{\substack{\textit{Factor loadings}\\ \textit{of Group 2}}}, \underbrace{\theta_{32}, \theta_{33}, \theta_{34}, \theta_{35}}_{\substack{\textit{Intercepts}\\ \textit{of Group 2}}}, \theta_{36}, \theta_{37}, \theta_{38},$$
$$\theta_{39}, \theta_{40}, \theta_{41}, \theta_{42}, \theta_{43}, \theta_{44}, \theta_{45}, \theta_{46})^T,$$

with $m = m_1 + m_2 = 2m_1 = 46$. Let $q^\star = 8$ be the number of factor loadings in each group, and $k^\star = 4$ the number of intercepts in each group. Notice that the factor loadings in $\boldsymbol{\theta}$ are located in the positions determined by $q = (g-1)m_1 + 1, \ldots, (g-1)m_1 + q^\star$, for $g = 1, 2$, that is, $q = 1, \ldots, 8, 24, \ldots, 31$. Define the matrix $\boldsymbol{R}_q$:

$$\boldsymbol{R}_q = \begin{array}{c} \\ 1 \\ \\ q \\ \\ \\ 46 \end{array} \begin{array}{c} \begin{array}{ccccc} 1 & & q & & 46 \end{array} \\ \begin{bmatrix} 0 & \ldots & 0 & \ldots & \ldots & 0 \\ \vdots & \ddots & \vdots & & & \vdots \\ 0 & \ldots & 1 & \ldots & \ldots & 0 \\ \vdots & & \vdots & \ddots & & \vdots \\ \vdots & & \vdots & & \ddots & \vdots \\ 0 & \ldots & 0 & \ldots & \ldots & 0 \end{bmatrix} \end{array} \qquad \text{for } q = 1, \ldots, 8, 24, \ldots, 31,$$

and $\boldsymbol{R}_q = \boldsymbol{O}_{46 \times 46}$ otherwise. Then, the penalty inducing sparsity on the factor loadings of each group is expressed as

$$\mathcal{P}_{\eta_1}(\boldsymbol{\theta}) = \sum_{q=1}^{46} \mathcal{P}_{\eta_1, q}(||\boldsymbol{R}_q \boldsymbol{\theta}||_1),$$

where $||\boldsymbol{R}_q \boldsymbol{\theta}||_1 = |\theta_q|$ for $q = 1, \ldots, 8, 24, \ldots, 31$, and 0 otherwise.

The pairwise differences of every loading across the two groups are $(\theta_q - \theta_{m_1+q})$, for $q = 1, \ldots, 8$, which consist of the set $\{(\theta_1 - \theta_{24}), (\theta_2 - \theta_{25}), (\theta_3 - \theta_{26}), (\theta_4 - \theta_{27}), (\theta_5 - \theta_{28}), (\theta_6 - \theta_{29}), (\theta_7 - \theta_{30}), (\theta_8 - \theta_{31})\}$. These differences can be specified through the matrix $\boldsymbol{D}_q^{\boldsymbol{\Lambda}}$, which, in case of two groups, for $q = 1, \ldots, 8$, is equal to:

$$
\boldsymbol{D}_q = [\boldsymbol{R}_q,\ -\boldsymbol{R}_q] =
\begin{array}{c}
 \\ 1 \\ \vdots \\ q \\ \vdots \\ \vdots \\ 23
\end{array}
\begin{array}{c}
\begin{array}{cccccc|ccccc}
1 & & q & & & 23 & & 23+q & & & 46
\end{array}
\\
\left[\begin{array}{cccccc|ccccc}
0 & \dots & 0 & \dots & \dots & 0 & \dots & 0 & \dots & \dots & 0 \\
\vdots & \ddots & \vdots & & & \vdots & & \vdots & & & \vdots \\
0 & \dots & 1 & \dots & \dots & 0 & \dots & -1 & \dots & \dots & 0 \\
\vdots & & \vdots & \ddots & & \vdots & & \vdots & \ddots & & \vdots \\
\vdots & & \vdots & & \ddots & \vdots & & \vdots & & \ddots & \vdots \\
0 & \dots & 0 & \dots & \dots & 0 & \dots & 0 & \dots & \dots & 0
\end{array}\right]
\end{array}, \qquad (B.2)
$$

and $\boldsymbol{D}_q^{\boldsymbol{\Lambda}} = \boldsymbol{O}_{23\times 46}$ otherwise. Then, the penalty inducing equal loadings across groups can be written as

$$\mathcal{P}_{\eta_2}(\boldsymbol{\theta}) = \sum_{q=1}^{46} \mathcal{P}_{\eta_2,q}(||\boldsymbol{D}_q^{\boldsymbol{\Lambda}}\boldsymbol{\theta}||_1),$$

where $||\boldsymbol{D}_q^{\boldsymbol{\Lambda}}\boldsymbol{\theta}||_1 = |\theta_q - \theta_{m_1+q}|$ for $q = 1, \dots, 8$, and 0 otherwise.

The pairwise differences of the intercepts across groups are computed similarly, the only difference being that the index q is now shifted by $q^\star$ units, that is, $q = (g-1)m_1 + q^\star + 1, \dots, (g-1)m_1 + q^\star + k^\star = 9, \dots, 12, 32, \dots, 35$. Then, the penalty introducing equal intercepts across groups is written as

$$\mathcal{P}_{\eta_3}(\boldsymbol{\theta}) = \sum_{q=1}^{46} \mathcal{P}_{\eta_3,q}(||\boldsymbol{D}_q^{\boldsymbol{\tau}}\boldsymbol{\theta}||_1),$$

where $\boldsymbol{D}_q^{\boldsymbol{\tau}}$ is equal to the matrix in (B.2) for $q = 9, \dots, 12$, and $\boldsymbol{D}_q^{\boldsymbol{\tau}} = \boldsymbol{O}_{23\times 46}$ otherwise, and $||\boldsymbol{D}_q^{\boldsymbol{\tau}}\boldsymbol{\theta}||_1 = |\theta_{q^\star+q} - \theta_{m_1+q^\star+q}|$ for $q = 9, \dots, 12$, and 0 otherwise.

Remark 1 (Fused penalty)**.** The first two penalties in (15) shrink the factor loadings within each group as well as their differences across groups. If $\mathcal{T} = L$, such penalty can be related to the generalized fused lasso proposed by Danaher, Wang and Witten (2014) in the context of multiple graphical models to penalize the off-diagonal elements of the precision matrices of different classes, as well as their differences across classes. On a different note, that penalty can be viewed as an extension of the pairwise fused lasso illustrated by Petry (2011) to penalize the coefficients of a general linear model as well as their differences among any pair of regressors.

Online Resource C Generalized Information Criterion

This section illustrates how the degrees of freedom of the penalized model can be found by deriving the bias term of the Generalized Information Criterion (GIC; Konishi & Kitagawa, 1996), an extension of the Akaike Information Criterion (AIC; Akaike, 1974) to the case where the estimation is not conducted through ordinary maximum likelihood. We follow the exposition in Konishi and Kitagawa (2008) and adapt it to the current context.

Suppose that N observations $\boldsymbol{x}_N = \{\boldsymbol{x}_1, \ldots, \boldsymbol{x}_\alpha, \ldots, \boldsymbol{x}_N\}$ generated from the unknown true distribution function $G(\boldsymbol{x})$ having density function $g(\boldsymbol{x})$ are realizations of the random vector $\boldsymbol{\mathcal{X}}_N = (\boldsymbol{X}_1, \ldots, \boldsymbol{X}_\alpha, \ldots, \boldsymbol{X}_N)^T$. In order to capture the structure of the given phenomena, we assume a parametric model that consists of a family of parametric distributions $\{f(\boldsymbol{x}|\boldsymbol{\theta}); \boldsymbol{\theta} \in \boldsymbol{\Theta} \subset \mathbb{R}^m\}$, where $\boldsymbol{\theta} = (\theta_1, \ldots, \theta_m)^T$ is the m-dimensional vector of unknown parameters and $\boldsymbol{\Theta}$ is an open subset of $\mathbb{R}^m$. We assume that the distribution $g(\boldsymbol{x})$ that generated the data is included in the class of parametric models, that is, there exists a parameter vector $\boldsymbol{\theta}_0 \in \boldsymbol{\Theta}$ such that $g(\boldsymbol{x}) = f(\boldsymbol{x}|\boldsymbol{\theta}_0)$. A statistical model $f(\boldsymbol{x}|\hat{\boldsymbol{\theta}})$ is then obtained by replacing the parameter vector $\boldsymbol{\theta}$ with the penalized maximum likelihood estimator (PMLE) $\hat{\boldsymbol{\theta}}$.

For convenience, we assume that each parameter θ_q in $\boldsymbol{\theta}$ can be expressed in the form of a real-valued function of the distribution of G, that is, the functional $T_q(G)$, where $T_q(G)$ is a function defined on the set of all distributions on the sample space and does not depend on the sample size N. Then, given data $\boldsymbol{x}_N = \{\boldsymbol{x}_1, \ldots, \boldsymbol{x}_\alpha, \ldots, \boldsymbol{x}_N\}$, the estimator $\hat{\theta}_q$ for the qth parameter θ_q is

$$\hat{\theta}_q = \hat{\theta}_q(\boldsymbol{x}_1, \ldots, \boldsymbol{x}_\alpha, \ldots, \boldsymbol{x}_N) = T_q(\hat{G}) \qquad \text{for} \;\; q = 1, \ldots, m,$$

in which the unknown probability distribution G has been replaced with the empirical distribution function $\hat{G}$ based on the data. The empirical distribution function is the distribution function for the probability function $\hat{g}(\boldsymbol{x}_\alpha) = \dfrac{1}{N}$ $(\alpha = 1, \ldots, N)$ that gives the equal probability $\dfrac{1}{N}$ for each of the N observations $\{\boldsymbol{x}_1, \ldots, \boldsymbol{x}_\alpha, \ldots, \boldsymbol{x}_N\}$. Because the estimator $\hat{\theta}_q = T_q(\hat{G})$ depends on the data only through the empirical distribution function $\hat{G}$, the functional is referred to as *statistical functional*.

Let us write the m-dimensional functional vector with $T_q(G)$ as the qth element as

$$\boldsymbol{T}(G) = (T_1(G), \ldots, T_q(G), \ldots, T_m(G))^T,$$

where $\boldsymbol{T}(G)$ is defined as the solution of the implicit equations

$$\int \boldsymbol{\psi}(\boldsymbol{x}, \boldsymbol{T}(G))dG(\boldsymbol{x}) = \mathbf{0}. \tag{C.1}$$

The function $\boldsymbol{\psi} = (\psi_1, \ldots, \psi_m)^T$ collects the real-valued functions $\psi_q(\boldsymbol{x}, \boldsymbol{T}(G))$ defined on the product space of the sample space and the parameter space $\boldsymbol{\Theta}$. The $\boldsymbol{\psi}$-function $\boldsymbol{\psi}(\boldsymbol{x}, \boldsymbol{T}(G))$ of the PMLE defined in Section 7 is

$$\begin{aligned}\boldsymbol{\psi}(\boldsymbol{x}, \boldsymbol{T}(G)) &= \frac{\partial}{\partial \boldsymbol{\theta}} \left\{\log f(\boldsymbol{x}|\boldsymbol{\theta}) - \mathcal{P}^{\mathcal{T}}_{\boldsymbol{\eta}}(\boldsymbol{\theta})\right\} \Big|_{\boldsymbol{\theta}=\boldsymbol{T}(G)} \\ &= \frac{\partial}{\partial \boldsymbol{\theta}} \left\{\log f(\boldsymbol{x}|\boldsymbol{\theta}) - \frac{1}{2}\boldsymbol{\theta}^T \boldsymbol{\mathcal{S}}^{\mathcal{T}}_{\boldsymbol{\eta}}(\tilde{\boldsymbol{\theta}})\boldsymbol{\theta}\right\} \Big|_{\boldsymbol{\theta}=\boldsymbol{T}(G)},\end{aligned}$$

where the penalty term $\mathcal{P}^{\mathcal{T}}_{\boldsymbol{\eta}}(\boldsymbol{\theta}) = \dfrac{1}{2}\boldsymbol{\theta}^T \boldsymbol{\mathcal{S}}^{\mathcal{T}}_{\boldsymbol{\eta}}(\tilde{\boldsymbol{\theta}})\boldsymbol{\theta}$ is a twice-continuously differentiable function, $\mathcal{T} = \{L, A, S, M\}$ and $\tilde{\boldsymbol{\theta}}$ is an initial value close to the true value of $\boldsymbol{\theta}$. In case of the normal linear factor model (Section 2), the log-likelihood of the sample is as in equation (2), the vector of the tuning parameters $\boldsymbol{\eta}$ reduces to the scalar η, and $\boldsymbol{\mathcal{S}}^{\mathcal{T}}_{\eta}(\tilde{\boldsymbol{\theta}})$ is as in equation (8). In case of the multiple-group factor model (Section 4), the log-likelihood of the sample is as in (14), the vector of tuning parameters $\boldsymbol{\eta}$ is equal to the triplet $(\eta_1, \eta_2, \eta_3)^T$, and $\boldsymbol{\mathcal{S}}^{\mathcal{T}}_{\boldsymbol{\eta}}(\tilde{\boldsymbol{\theta}}) = \boldsymbol{\mathcal{D}}^{\mathcal{T}}_{\eta_1}(\tilde{\boldsymbol{\theta}}) + \boldsymbol{\mathcal{D}}^{\mathcal{T}}_{\eta_2}(\tilde{\boldsymbol{\theta}}) + \boldsymbol{\mathcal{D}}^{\mathcal{T}}_{\eta_3}(\tilde{\boldsymbol{\theta}})$. Then, the m-dimensional PMLE $\hat{\boldsymbol{\theta}}$ can be expressed as

$$\hat{\boldsymbol{\theta}} = \boldsymbol{T}(\hat{G}) = (T_1(\hat{G}), \ldots, T_q(\hat{G}), \ldots, T_m(\hat{G}))^T,$$

where $\boldsymbol{T}(\hat{G})$ is defined as the solution of the system of penalized likelihood equations

$$\sum_{\alpha=1}^{N} \boldsymbol{\psi}(\boldsymbol{x}_\alpha, \boldsymbol{T}(\hat{G})) = \sum_{\alpha=1}^{N} \boldsymbol{\psi}(\boldsymbol{x}_\alpha, \hat{\boldsymbol{\theta}}) = \mathbf{0},$$

with

$$\boldsymbol{\psi}(\boldsymbol{x}_\alpha, \hat{\boldsymbol{\theta}}) = \frac{\partial}{\partial \boldsymbol{\theta}} \left\{ \log f(\boldsymbol{x}_\alpha|\boldsymbol{\theta}) - \frac{1}{2}\boldsymbol{\theta}^T \boldsymbol{\mathcal{S}}_{\boldsymbol{\eta}}^{\mathcal{T}}(\boldsymbol{\theta})\boldsymbol{\theta} \right\} \Bigg|_{\boldsymbol{\theta}=\hat{\boldsymbol{\theta}}}.$$

Once the model has been constructed, the interest usually lies in its evaluation from the standpoint of making a prediction. The idea is thus to evaluate the expected goodness of the estimated model $f(\boldsymbol{z}|\hat{\boldsymbol{\theta}})$ when it is used to predict the independent future data $\boldsymbol{Z} = \boldsymbol{z}$ generated from the unknown true distribution $g(\boldsymbol{z})$. Specifically, the goodness of the statistical model $f(\boldsymbol{z}|\hat{\boldsymbol{\theta}})$ can be assessed by evaluating its closeness to the true distribution $g(\boldsymbol{z})$ in terms of the Kullback-Leibler (K-L) information

$$\begin{aligned} I(g(\boldsymbol{z}); f(\boldsymbol{z}|\hat{\boldsymbol{\theta}})) &:= \mathbb{E}_{G(\boldsymbol{z})}\left[\log\left\{\frac{g(\boldsymbol{Z})}{f(\boldsymbol{Z}|\hat{\boldsymbol{\theta}})}\right\}\right] = \int \log\left\{\frac{g(\boldsymbol{z})}{f(\boldsymbol{z}|\hat{\boldsymbol{\theta}})}\right\} g(\boldsymbol{z})d\boldsymbol{z} \\ &= \int g(\boldsymbol{z}) \log g(\boldsymbol{z}) d\boldsymbol{z} - \int g(\boldsymbol{z}) \log f\left(\boldsymbol{z}|\hat{\boldsymbol{\theta}}\right) d\boldsymbol{z}, \end{aligned} \tag{C.2}$$

where the expectation is taken with respect to the unknown true probability distribution function $G(\boldsymbol{z})$. Because the first term on the right-hand side of equation (C.2) is a constant that depends solely on the true model g, in order to compare different models it is sufficient to consider only the second term on the right-hand side, called the expected log-likelihood:

$$\begin{aligned} \varphi(\boldsymbol{\mathcal{X}}_N; G) := \mathbb{E}_{G(\boldsymbol{z})}[\log f(\boldsymbol{Z}|\hat{\boldsymbol{\theta}}(\boldsymbol{\mathcal{X}}_N))] &= \int g(\boldsymbol{z}) \log f(\boldsymbol{z}|\hat{\boldsymbol{\theta}}) d\boldsymbol{z} \\ &= \int \log f(\boldsymbol{z}|\hat{\boldsymbol{\theta}}) dG(\boldsymbol{z}). \end{aligned} \tag{C.3}$$

The larger this value is for a model, the smaller its K-L information and the closer the model is to the true one. The expected log-likelihood still depends on the true distribution g and is an unknown quantity that eludes explicit computation. A good estimate of the expected log-likelihood can be obtained from the data by replacing G with $\hat{G}$, that is,

$$\begin{aligned} \varphi(\boldsymbol{\mathcal{X}}_N; \hat{G}) &= \mathbb{E}_{\hat{G}}[\log f(\boldsymbol{Z}|\hat{\boldsymbol{\theta}})] = \int \log f(\boldsymbol{z}|\hat{\boldsymbol{\theta}}) d\hat{G}(\boldsymbol{z}) \\ &= \sum_{\alpha=1}^{N} \hat{g}(\boldsymbol{x}_\alpha) \log f(\boldsymbol{x}_\alpha|\hat{\boldsymbol{\theta}}) = \frac{1}{N}\sum_{\alpha=1}^{N} \log f(\boldsymbol{x}_\alpha|\hat{\boldsymbol{\theta}}). \end{aligned} \tag{C.4}$$

According to the law of large numbers, when the number of observations N tends to infinity, the

mean of the random variables $\boldsymbol{Y}_\alpha = \log f(\boldsymbol{X}_\alpha)$ $(\alpha = 1, \ldots, N)$ converges in probability to its expectation, that is,

$$\begin{aligned}
\varphi(\boldsymbol{\mathcal{X}}_N; \hat{G}) &= \frac{1}{N}\sum_{\alpha=1}^{N} \log f(\boldsymbol{X}_\alpha) \\
&= \frac{1}{N}\log f(\boldsymbol{\mathcal{X}}_N|\hat{\boldsymbol{\theta}}(\boldsymbol{\mathcal{X}}_N)) \xrightarrow{N\to\infty} \mathbb{E}_G[\log f(\boldsymbol{Z}|\hat{\boldsymbol{\theta}})] = \varphi(\boldsymbol{\mathcal{X}}_N; G).
\end{aligned}$$

Therefore, the estimate based on the empirical distribution function is a natural estimate of the expected log-likelihood. The estimate of the expected log-likelihood multiplied by N is the log-likelihood of the statistical model $f(\boldsymbol{z}|\hat{\boldsymbol{\theta}}(\boldsymbol{x}_N))$

$$N\int \log f(\boldsymbol{z}|\hat{\boldsymbol{\theta}})d\hat{G}(\boldsymbol{z}) = \sum_{\alpha=1}^{N} \log f(\boldsymbol{x}_\alpha|\hat{\boldsymbol{\theta}}(\boldsymbol{x}_N)) = \log f(\boldsymbol{x}_N|\hat{\boldsymbol{\theta}}(\boldsymbol{x}_N)) = \ell(\hat{\boldsymbol{\theta}}).$$

It is worth noting that the estimator of the expected log-likelihood $\mathbb{E}_G[\log f(\boldsymbol{Z}|\hat{\boldsymbol{\theta}})]$ is $\dfrac{1}{N}\ell(\hat{\boldsymbol{\theta}})$ and that the log-likelihood $\ell(\hat{\boldsymbol{\theta}})$ is an estimator of $N\,\mathbb{E}_G[\log f(\boldsymbol{Z}|\hat{\boldsymbol{\theta}})]$.

In this procedure, the log-likelihood in (C.4) was obtained by estimating the expected log-likelihood $\mathbb{E}_G[\log f(\boldsymbol{Z}|\hat{\boldsymbol{\theta}})]$ by reusing the data $\boldsymbol{x}_N$ that were initially used to estimate the model $f(\boldsymbol{Z}|\hat{\boldsymbol{\theta}})$ in place of the future data. The use of the same data twice for estimating the parameters and the evaluation measure (expected log-likelihood) of the goodness of the estimated model gives rise to bias. Specifically, the bias of the log-likelihood as an estimator of the expected log-likelihood given in (C.3) is defined as

$$\begin{aligned}
b(G) &:= \mathbb{E}_G\{\varphi(\boldsymbol{\mathcal{X}}_N; \hat{G}) - \varphi(\boldsymbol{\mathcal{X}}_N; G)\} \\
&= \mathbb{E}_{G(\boldsymbol{x}_N)}\left[\frac{1}{N}\log f(\boldsymbol{\mathcal{X}}_N|\hat{\boldsymbol{\theta}}(\boldsymbol{\mathcal{X}}_N)) - \mathbb{E}_{G(\boldsymbol{z})}[\log f(\boldsymbol{Z}|\hat{\boldsymbol{\theta}}(\boldsymbol{\mathcal{X}}_N))]\right],
\end{aligned}$$

where the expectation $\mathbb{E}_{G(\boldsymbol{x}_N)}$ is taken with respect to the joint distribution $G(\boldsymbol{x}_N) = \prod_{\alpha=1}^{N} G(\boldsymbol{x}_\alpha)$ of the sample $\boldsymbol{\mathcal{X}}_N$. The prerequisite for a fair comparison of models is thus the evaluation of and the correction for this bias term. The general form of the Generalized Information Criterion, which is defined as a bias-corrected log-likelihood, can be constructed by evaluating the bias and correcting for it as follows:

$$
\begin{aligned}
GIC(\boldsymbol{\mathcal{X}}_N;\hat{G}) &= -2N\left(\frac{1}{N}\sum_{\alpha=1}^{N}\log f(\boldsymbol{X}_\alpha|\hat{\boldsymbol{\theta}}) - b(\hat{G})\right) \\
&= -2\sum_{\alpha=1}^{N}\log f(\boldsymbol{X}_\alpha|\hat{\boldsymbol{\theta}}) + 2N\,b(\hat{G}). \qquad \text{(C.5)}
\end{aligned}
$$

The GIC represents an extension of the AIC (see Konishi & Kitagawa, 2008 for a full exposition on the topic). In the same spirit, we can formulate a Generalized Bayesian Information Criterion (GBIC) as an extension of the Bayesian Information Criterion (BIC; Schwarz, 1978)

$$
GBIC(\boldsymbol{\mathcal{X}}_N;\hat{G}) = -2\sum_{\alpha=1}^{N}\log f(\boldsymbol{X}_\alpha|\hat{\boldsymbol{\theta}}) + \log(N)N\,b(\hat{G}), \qquad \text{(C.6)}
$$

by changing the weight given to the bias term $b(\hat{G})$ from 2 to $\log(N)$ used in the BIC.

Konishi and Kitagawa (1996) showed that the asymptotic bias of the log-likelihood in the estimation of the expected log-likelihood can be represented as the integral of the product of the influence function of the employed estimator and the score function of the probability model, i.e.,

$$
\begin{aligned}
\mathbb{E}_G[\varphi(\boldsymbol{\mathcal{X}}_N;\hat{G}) - \varphi(\boldsymbol{\mathcal{X}}_N;G)] &= \left[\frac{1}{N}\sum_{\alpha=1}^{N}\log f(\boldsymbol{X}_\alpha|\hat{\boldsymbol{\theta}}) - \int \log f(\boldsymbol{z}|\hat{\boldsymbol{\theta}})dG(\boldsymbol{z})\right] \\
&= \frac{1}{N}b_1(G) + o\left(\frac{1}{N}\right),
\end{aligned}
$$

where

$$
b_1(G) = \operatorname{tr}\left\{\int \boldsymbol{T}^{(1)}(\boldsymbol{z};G)\frac{\partial \log f(\boldsymbol{z}|\boldsymbol{\theta})}{\partial \boldsymbol{\theta}^T}\Big|_{\boldsymbol{\theta}=\boldsymbol{T}(G)} dG(\boldsymbol{z})\right\}. \qquad \text{(C.7)}
$$

The quantity $\boldsymbol{T}^{(1)}(\boldsymbol{z};G)$ is the influence function of the m-dimensional functional $\boldsymbol{T}(G)$ at the true distribution G. The influence function $\boldsymbol{T}^{(1)}(\boldsymbol{z};G) = (T_1^{(1)}(\boldsymbol{z};G),\dots,T_q^{(1)}(\boldsymbol{z};G),\dots,T_m^{(1)}(\boldsymbol{z};G))^T$ describes the effect of an infinitesimal contamination at $\boldsymbol{z}$. Its components $T_q^{(1)}(\boldsymbol{z},G)$ $(q = 1,\dots,m)$ are defined in terms of the directional derivative of the functional $T_q(G)$ with respect to G, that is,

$$
\begin{aligned}
\lim_{\epsilon\to 0}\frac{T_q((1-\epsilon)G+\epsilon\delta_{\boldsymbol{z}}) - T_q(G)}{\epsilon} &= \frac{\partial}{\partial\epsilon}\{T_q((1-\epsilon)G+\epsilon\delta_{\boldsymbol{z}})\}\Big|_{\epsilon=0} \\
&= \int T_q^{(1)}(\boldsymbol{z};G)d\delta_{\boldsymbol{z}} := T_q^{(1)}(\boldsymbol{z};G),
\end{aligned}
$$

where $\delta_{\boldsymbol{z}}$ is a point mass at $\boldsymbol{z}$.

The expression of the influence function of the PMLE can be found by calculating the derivative of the corresponding functional. Firstly, substitute $(1-\epsilon)G + \epsilon\delta_{\boldsymbol{z}}$ for G in equation (C.1):

$$\begin{aligned}\int \boldsymbol{\psi}(\boldsymbol{x}, \boldsymbol{T}((1-\epsilon)G+\epsilon\delta_{\boldsymbol{z}}))d\{(1-\epsilon)G(\boldsymbol{x})+\epsilon\delta_{\boldsymbol{z}}(\boldsymbol{x})\} &= \\ \int \frac{\partial}{\partial\boldsymbol{\theta}}\left[\log f(\boldsymbol{x}|\boldsymbol{\theta}) - \frac{1}{2}\boldsymbol{\theta}^T\boldsymbol{\mathcal{S}}_{\eta}^{\mathcal{T}}(\tilde{\boldsymbol{\theta}})\boldsymbol{\theta}\right]\Bigg|_{\boldsymbol{\theta}=\boldsymbol{T}((1-\epsilon)G+\epsilon\delta_{\boldsymbol{z}})} d\{(1-\epsilon)G(\boldsymbol{x})+\epsilon\delta_{\boldsymbol{z}}(\boldsymbol{x})\} &= \mathbf{0}.\end{aligned}$$

Secondly, differentiate both sides of the equation with respect to ϵ:

$$\int \frac{\partial}{\partial\epsilon}\left\{\frac{\partial}{\partial\boldsymbol{\theta}}\left[\log f(\boldsymbol{x}|\boldsymbol{\theta}) - \frac{1}{2}\boldsymbol{\theta}^T\boldsymbol{\mathcal{S}}_{\eta}^{\mathcal{T}}(\tilde{\boldsymbol{\theta}})\boldsymbol{\theta}\right]\Bigg|_{\boldsymbol{\theta}=\boldsymbol{T}((1-\epsilon)G+\epsilon\delta_{\boldsymbol{z}})} d\{(1-\epsilon)G(\boldsymbol{x})+\epsilon\delta_{\boldsymbol{z}}(\boldsymbol{x})\}\right\} = \mathbf{0}$$

$$\begin{aligned}&\int \frac{\partial}{\partial\boldsymbol{\theta}}\left[\log f(\boldsymbol{x}|\boldsymbol{\theta}) - \frac{1}{2}\boldsymbol{\theta}^T\boldsymbol{\mathcal{S}}_{\eta}^{\mathcal{T}}(\tilde{\boldsymbol{\theta}})\boldsymbol{\theta}\right]\Bigg|_{\boldsymbol{\theta}=\boldsymbol{T}((1-\epsilon)G+\epsilon\delta_{\boldsymbol{z}})} \frac{\partial}{\partial\epsilon} d\{(1-\epsilon)G(\boldsymbol{x})+\epsilon\delta_{\boldsymbol{z}}(\boldsymbol{x})\} \\ &+ \int \frac{\partial}{\partial\epsilon}\left\{\frac{\partial}{\partial\boldsymbol{\theta}}\left[\log f(\boldsymbol{x}|\boldsymbol{\theta}) - \frac{1}{2}\boldsymbol{\theta}^T\boldsymbol{\mathcal{S}}_{\eta}^{\mathcal{T}}(\tilde{\boldsymbol{\theta}})\boldsymbol{\theta}\right]\Bigg|_{\boldsymbol{\theta}=\boldsymbol{T}((1-\epsilon)G+\epsilon\delta_{\boldsymbol{z}})}\right\} d\{(1-\epsilon)G(\boldsymbol{x})+\epsilon\delta_{\boldsymbol{z}}(\boldsymbol{x})\} = \mathbf{0}\end{aligned}$$

$$\begin{aligned}&\int \frac{\partial}{\partial\boldsymbol{\theta}}\left[\log f(\boldsymbol{x}|\boldsymbol{\theta}) - \frac{1}{2}\boldsymbol{\theta}^T\boldsymbol{\mathcal{S}}_{\eta}^{\mathcal{T}}(\tilde{\boldsymbol{\theta}})\boldsymbol{\theta}\right]\Bigg|_{\boldsymbol{\theta}=\boldsymbol{T}((1-\epsilon)G+\epsilon\delta_{\boldsymbol{z}})} d\{-G(\boldsymbol{x})+\delta_{\boldsymbol{z}}(\boldsymbol{x})\} \\ &+ \int \frac{\partial}{\partial\boldsymbol{\theta}^T}\frac{\partial}{\partial\boldsymbol{\theta}}\left[\log f(\boldsymbol{x}|\boldsymbol{\theta}) - \frac{1}{2}\boldsymbol{\theta}^T\boldsymbol{\mathcal{S}}_{\eta}^{\mathcal{T}}(\tilde{\boldsymbol{\theta}})\boldsymbol{\theta}\right]\Bigg|_{\boldsymbol{\theta}=\boldsymbol{T}((1-\epsilon)G+\epsilon\delta_{\boldsymbol{z}})} \\ &\qquad \times \frac{\partial}{\partial\epsilon}\{\boldsymbol{T}((1-\epsilon)G+\epsilon\delta_{\boldsymbol{z}})\} d\{(1-\epsilon)G(\boldsymbol{x})+\epsilon\delta_{\boldsymbol{z}}(\boldsymbol{x})\} = \mathbf{0}.\end{aligned}$$

Then set $\epsilon = 0$:

$$\begin{aligned}&\int \frac{\partial}{\partial\boldsymbol{\theta}}\left[\log f(\boldsymbol{x}|\boldsymbol{\theta}) - \frac{1}{2}\boldsymbol{\theta}^T\boldsymbol{\mathcal{S}}_{\eta}^{\mathcal{T}}(\tilde{\boldsymbol{\theta}})\boldsymbol{\theta}\right]\Bigg|_{\boldsymbol{\theta}=\boldsymbol{T}(G)} d\{\delta_{\boldsymbol{z}}(\boldsymbol{x})-G(\boldsymbol{x})\} \\ &+ \int \frac{\partial}{\partial\boldsymbol{\theta}}\frac{\partial}{\partial\boldsymbol{\theta}^T}\left[\log f(\boldsymbol{x}|\boldsymbol{\theta}) - \frac{1}{2}\boldsymbol{\theta}^T\boldsymbol{\mathcal{S}}_{\eta}^{\mathcal{T}}(\tilde{\boldsymbol{\theta}})\boldsymbol{\theta}\right]\Bigg|_{\boldsymbol{\theta}=\boldsymbol{T}(G)} \frac{\partial}{\partial\epsilon}\{\boldsymbol{T}((1-\epsilon)G+\epsilon\delta_{\boldsymbol{z}})\}\Big|_{\epsilon=0} dG(\boldsymbol{x}) = \mathbf{0}\end{aligned}$$

$$
\int \frac{\partial}{\partial \boldsymbol{\theta}} \left[\log f(\boldsymbol{x}|\boldsymbol{\theta}) - \frac{1}{2}\boldsymbol{\theta}^T \boldsymbol{\mathcal{S}}_{\boldsymbol{\eta}}^{\mathcal{T}}(\tilde{\boldsymbol{\theta}})\boldsymbol{\theta} \right] \Big|_{\boldsymbol{\theta}=\boldsymbol{T}(G)} d\delta_{\boldsymbol{z}}(\boldsymbol{x})
$$

$$
\underbrace{- \int \frac{\partial}{\partial \boldsymbol{\theta}} \left[\log f(\boldsymbol{x}|\boldsymbol{\theta}) - \frac{1}{2}\boldsymbol{\theta}^T \boldsymbol{\mathcal{S}}_{\boldsymbol{\eta}}^{\mathcal{T}}(\tilde{\boldsymbol{\theta}})\boldsymbol{\theta} \right] \Big|_{\boldsymbol{\theta}=\boldsymbol{T}(G)} dG(\boldsymbol{x})}_{=0 \text{ by eq. (C.1)}}
$$

$$
+ \int \frac{\partial^2}{\partial \boldsymbol{\theta} \partial \boldsymbol{\theta}^T} \left[\log f(\boldsymbol{x}|\boldsymbol{\theta}) - \frac{1}{2}\boldsymbol{\theta}^T \boldsymbol{\mathcal{S}}_{\boldsymbol{\eta}}^{\mathcal{T}}(\tilde{\boldsymbol{\theta}})\boldsymbol{\theta} \right] \Big|_{\boldsymbol{\theta}=\boldsymbol{T}(G)} dG(\boldsymbol{x}) \frac{\partial}{\partial \epsilon} \left\{ \boldsymbol{T}((1-\epsilon)G + \epsilon\delta_{\boldsymbol{z}}) \right\} \Big|_{\epsilon=0} = \mathbf{0}
$$

$$
\int \frac{\partial}{\partial \boldsymbol{\theta}} \left[\log f(\boldsymbol{x}|\boldsymbol{\theta}) - \frac{1}{2}\boldsymbol{\theta}^T \boldsymbol{\mathcal{S}}_{\boldsymbol{\eta}}^{\mathcal{T}}(\tilde{\boldsymbol{\theta}})\boldsymbol{\theta} \right] \Big|_{\boldsymbol{\theta}=\boldsymbol{T}(G)} d\delta_{\boldsymbol{z}}(\boldsymbol{x})
$$

$$
+ \int \frac{\partial^2}{\partial \boldsymbol{\theta} \partial \boldsymbol{\theta}^T} \left[\log f(\boldsymbol{x}|\boldsymbol{\theta}) - \frac{1}{2}\boldsymbol{\theta}^T \boldsymbol{\mathcal{S}}_{\boldsymbol{\eta}}^{\mathcal{T}}(\tilde{\boldsymbol{\theta}})\boldsymbol{\theta} \right] \Big|_{\boldsymbol{\theta}=\boldsymbol{T}(G)} dG(\boldsymbol{x}) \frac{\partial}{\partial \epsilon} \left\{ \boldsymbol{T}((1-\epsilon)G + \epsilon\delta_{\boldsymbol{z}}) \right\} \Big|_{\epsilon=0} = \mathbf{0}
$$

$$
\frac{\partial}{\partial \boldsymbol{\theta}} \left[\log f(\boldsymbol{z}|\boldsymbol{\theta}) - \frac{1}{2}\boldsymbol{\theta}^T \boldsymbol{\mathcal{S}}_{\boldsymbol{\eta}}^{\mathcal{T}}(\tilde{\boldsymbol{\theta}})\boldsymbol{\theta} \right] \Big|_{\boldsymbol{\theta}=\boldsymbol{T}(G)}
$$

$$
+ \int \frac{\partial^2}{\partial \boldsymbol{\theta} \partial \boldsymbol{\theta}^T} \left[\log f(\boldsymbol{x}|\boldsymbol{\theta}) - \frac{1}{2}\boldsymbol{\theta}^T \boldsymbol{\mathcal{S}}_{\boldsymbol{\eta}}^{\mathcal{T}}(\tilde{\boldsymbol{\theta}})\boldsymbol{\theta} \right] \Big|_{\boldsymbol{\theta}=\boldsymbol{T}(G)} dG(\boldsymbol{x}) \underbrace{\frac{\partial}{\partial \epsilon} \left\{ \boldsymbol{T}((1-\epsilon)G + \epsilon\delta_{z}) \right\} \Big|_{\epsilon=0}}_{=\boldsymbol{T}^{(1)}(\boldsymbol{z};G)} = \mathbf{0}.
$$

Consequently, the influence function $\boldsymbol{T}^{(1)}(\boldsymbol{z}; G)$ that defines the PMLE is given by

$$
\begin{aligned}
\boldsymbol{T}^{(1)}(\boldsymbol{z}; G) &:= \frac{\partial}{\partial \epsilon} \left\{ \boldsymbol{T}((1-\epsilon)G + \epsilon\delta_{\boldsymbol{z}}) \right\} \Big|_{\epsilon=0} \\
&= - \left\{ \int \frac{\partial^2}{\partial \boldsymbol{\theta} \partial \boldsymbol{\theta}^T} \left[\log f(\boldsymbol{z}|\boldsymbol{\theta}) - \frac{1}{2}\boldsymbol{\theta}^T \boldsymbol{\mathcal{S}}_{\boldsymbol{\eta}}^{\mathcal{T}}(\tilde{\boldsymbol{\theta}})\boldsymbol{\theta} \right] \Big|_{\boldsymbol{\theta}=\boldsymbol{T}(G)} dG(\boldsymbol{z}) \right\}^{-1} \\
&\qquad \times \left\{ \frac{\partial}{\partial \boldsymbol{\theta}} \left[\log f(\boldsymbol{z}|\boldsymbol{\theta}) - \frac{1}{2}\boldsymbol{\theta}^T \boldsymbol{\mathcal{S}}_{\boldsymbol{\eta}}^{\mathcal{T}}(\tilde{\boldsymbol{\theta}})\boldsymbol{\theta} \right] \Big|_{\boldsymbol{\theta}=\boldsymbol{T}(G)} \right\} \\
&= \boldsymbol{R}(\boldsymbol{\psi}, G)^{-1} \boldsymbol{\psi}(\boldsymbol{z}; \boldsymbol{T}(G)),
\end{aligned} \tag{C.8}
$$

where $\boldsymbol{R}(\boldsymbol{\psi}, G)$ is an $m \times m$ matrix defined as

$$
\begin{aligned}
\boldsymbol{R}(\boldsymbol{\psi}, G) &= - \int \frac{\partial \boldsymbol{\psi}(\boldsymbol{z}, \boldsymbol{\theta})^T}{\partial \boldsymbol{\theta}} \Big|_{\boldsymbol{\theta}=\boldsymbol{T}(G)} dG(\boldsymbol{z}) \\
&= - \int \frac{\partial^2 \log f(\boldsymbol{z}|\boldsymbol{\theta})}{\partial \boldsymbol{\theta} \partial \boldsymbol{\theta}^T} \Big|_{\boldsymbol{\theta}=\boldsymbol{T}(G)} dG(\boldsymbol{z}) + \int \frac{\partial^2}{\partial \boldsymbol{\theta} \partial \boldsymbol{\theta}^T} \left(\frac{1}{2}\boldsymbol{\theta}^T \boldsymbol{\mathcal{S}}_{\boldsymbol{\eta}}^{\mathcal{T}}(\tilde{\boldsymbol{\theta}})\boldsymbol{\theta} \right) \Big|_{\boldsymbol{\theta}=\boldsymbol{T}(G)} dG(\boldsymbol{z}).
\end{aligned}
$$

More specifically, for the normal linear factor model, if we denote $\boldsymbol{\theta} = (\boldsymbol{\theta}^\star, \check{\boldsymbol{\theta}})^T$, where $\boldsymbol{\theta}^\star$ collects the penalized parameters and $\check{\boldsymbol{\theta}}$ the unpenalized parameters, we have that:

$$\frac{\partial \boldsymbol{\psi}(\boldsymbol{z}, \boldsymbol{\theta})^T}{\partial \boldsymbol{\theta}} = \begin{bmatrix} \dfrac{\partial^2 \log f(\boldsymbol{z}|\boldsymbol{\theta})}{\partial \boldsymbol{\theta}^\star \partial \boldsymbol{\theta}^{\star T}} - \boldsymbol{\mathcal{M}}_\eta^{\mathcal{T}}(\tilde{\boldsymbol{\theta}}) & \dfrac{\partial^2 \log f(\boldsymbol{z}|\boldsymbol{\theta})}{\partial \boldsymbol{\theta}^\star \partial \check{\boldsymbol{\theta}}^T} \\ \dfrac{\partial^2 \log f(\boldsymbol{z}|\boldsymbol{\theta})}{\partial \check{\boldsymbol{\theta}} \partial \boldsymbol{\theta}^{\star T}} & \dfrac{\partial^2 \log f(\boldsymbol{z}|\boldsymbol{\theta})}{\partial \check{\boldsymbol{\theta}} \partial \check{\boldsymbol{\theta}}^T} \end{bmatrix},$$

where $\boldsymbol{\mathcal{M}}_\eta^{\mathcal{T}}(\tilde{\boldsymbol{\theta}})$ is the sub-matrix of $\boldsymbol{\mathcal{S}}_\eta^{\mathcal{T}}(\tilde{\boldsymbol{\theta}})$ corresponding to the penalized parameters defined in Section 3.1, and the tuning parameter vector $\boldsymbol{\eta}$ reduces to the scalar η.

By substituting the expression of the influence function of the PMLE into equation (C.7), we get the following expression of the bias:

$$\begin{aligned} b_1(G) &= \operatorname{tr}\left\{\int \boldsymbol{T}^{(1)}(\boldsymbol{z}; G) \frac{\partial \log f(\boldsymbol{z}|\boldsymbol{\theta})}{\partial \boldsymbol{\theta}^T}\Big|_{\boldsymbol{\theta}=\boldsymbol{T}(G)} dG(\boldsymbol{z})\right\} \\ &= \operatorname{tr}\left\{\int \boldsymbol{R}(\boldsymbol{\psi}, G)^{-1} \boldsymbol{\psi}(\boldsymbol{z}, \boldsymbol{T}(G)) \frac{\partial \log f(\boldsymbol{z}|\boldsymbol{\theta})}{\partial \boldsymbol{\theta}^T}\Big|_{\boldsymbol{\theta}=\boldsymbol{T}(G)} dG(\boldsymbol{z})\right\} \\ &= \operatorname{tr}\left\{\boldsymbol{R}(\boldsymbol{\psi}, G)^{-1} \int \boldsymbol{\psi}(\boldsymbol{z}; \boldsymbol{T}(G)) \frac{\partial \log f(\boldsymbol{z}|\boldsymbol{\theta})}{\partial \boldsymbol{\theta}^T}\Big|_{\boldsymbol{\theta}=\boldsymbol{T}(G)} dG(\boldsymbol{z})\right\} \\ &= \operatorname{tr}\left\{\boldsymbol{R}(\boldsymbol{\psi}, G)^{-1} \boldsymbol{Q}(\boldsymbol{\psi}, G)\right\}, \end{aligned}$$

where $\boldsymbol{Q}(\boldsymbol{\psi}, G)$ is an $m \times m$ matrix defined as

$$\begin{aligned} \boldsymbol{Q}(\boldsymbol{\psi}, G) &= \int \boldsymbol{\psi}(\boldsymbol{z}; \boldsymbol{T}(G)) \frac{\partial \log f(\boldsymbol{z}|\boldsymbol{\theta})}{\partial \boldsymbol{\theta}^T}\Big|_{\boldsymbol{\theta}=\boldsymbol{T}(G)} dG(\boldsymbol{z}) \\ &= \int \frac{\partial}{\partial \boldsymbol{\theta}} \left\{\log f(\boldsymbol{z}|\boldsymbol{\theta}) - \frac{1}{2} \boldsymbol{\theta}^T \boldsymbol{\mathcal{S}}_{\boldsymbol{\eta}}^{\mathcal{T}}(\tilde{\boldsymbol{\theta}}) \boldsymbol{\theta}\right\}\Big|_{\boldsymbol{\theta}=\boldsymbol{T}(G)} \frac{\partial \log f(\boldsymbol{z}|\boldsymbol{\theta})}{\partial \boldsymbol{\theta}^T}\Big|_{\boldsymbol{\theta}=\boldsymbol{T}(G)} dG(\boldsymbol{z}) \\ &= \int \frac{\partial \log f(\boldsymbol{z}|\boldsymbol{\theta})}{\partial \boldsymbol{\theta}} \frac{\partial \log f(\boldsymbol{z}|\boldsymbol{\theta})}{\partial \boldsymbol{\theta}^T}\Big|_{\boldsymbol{\theta}=\boldsymbol{T}(G)} dG(\boldsymbol{z}) \\ &\qquad - \int \boldsymbol{\mathcal{S}}_{\boldsymbol{\eta}}^{\mathcal{T}}(\tilde{\boldsymbol{\theta}}) \boldsymbol{T}(G) \frac{\partial \log f(\boldsymbol{z}|\boldsymbol{\theta})}{\partial \boldsymbol{\theta}^T}\Big|_{\boldsymbol{\theta}=\boldsymbol{T}(G)} dG(\boldsymbol{z}) \\ &= \int \frac{\partial \log f(\boldsymbol{z}|\boldsymbol{\theta})}{\partial \boldsymbol{\theta}} \frac{\partial \log f(\boldsymbol{z}|\boldsymbol{\theta})}{\partial \boldsymbol{\theta}^T}\Big|_{\boldsymbol{\theta}=\boldsymbol{T}(G)} dG(\boldsymbol{z}) \\ &= -\int \frac{\partial^2 \log f(\boldsymbol{z}|\boldsymbol{\theta})}{\partial \boldsymbol{\theta} \partial \boldsymbol{\theta}^T}\Big|_{\boldsymbol{\theta}=\boldsymbol{T}(G)} dG(\boldsymbol{z}) = \boldsymbol{Q}(G). \end{aligned}$$

The fourth line follows from the fact that as $N \to \infty$

$$\mathbf{0} = \int \frac{\partial}{\partial \boldsymbol{\theta}} \left[\log f(\boldsymbol{z}|\boldsymbol{\theta}) - \left(\frac{1}{2} \boldsymbol{\theta}^T \boldsymbol{\mathcal{S}}_{\boldsymbol{\eta}}^{\mathcal{T}}(\tilde{\boldsymbol{\theta}}) \boldsymbol{\theta} \right) \right] \bigg|_{\boldsymbol{\theta}=\boldsymbol{T}(G)} dG(\boldsymbol{z}) = \int \frac{\partial \log f(\boldsymbol{z}|\boldsymbol{\theta})}{\partial \boldsymbol{\theta}} \bigg|_{\boldsymbol{\theta}=\boldsymbol{T}(G)} dG(\boldsymbol{z}).$$

Let $b_1(\hat{G})$ be a bias estimate obtained by replacing the unknown distribution G with the empirical distribution $\hat{G}$:

$$\begin{aligned} b_1(\hat{G}) &= \operatorname{tr} \left\{ \frac{1}{N} \sum_{\alpha=1}^{N} \boldsymbol{T}^{(1)}(\boldsymbol{x}_\alpha, \hat{G}) \frac{\partial \log f(\boldsymbol{x}_\alpha|\boldsymbol{\theta})}{\partial \boldsymbol{\theta}^T} \bigg|_{\boldsymbol{\theta}=\boldsymbol{T}(\hat{G})} \right\} \\ &= \operatorname{tr} \left\{ \boldsymbol{R}(\boldsymbol{\psi}, \hat{G})^{-1} \boldsymbol{Q}(\hat{G}) \right\}. \end{aligned} \tag{C.9}$$

The quantity $\boldsymbol{T}^{(1)}(\boldsymbol{x}_\alpha, \hat{G})$ represents the vector of empirical influence functions, whose components $T_q^{(1)}(\boldsymbol{x}_\alpha, \hat{G})$ are defined as the derivative of $T_q(\hat{G})$ with respect to the probability measure $\delta_{\boldsymbol{x}_\alpha}$ being the point mass at $\boldsymbol{x}_\alpha$, that is,

$$T_q^{(1)}(\boldsymbol{x}_\alpha, \hat{G}) = \lim_{\epsilon \to 0} \frac{T_q((1-\epsilon)\hat{G} + \epsilon \delta_{\boldsymbol{x}_\alpha}) - T_q(\hat{G})}{\epsilon}.$$

The matrices $\boldsymbol{R}(\boldsymbol{\psi}, \hat{G})$ and $\boldsymbol{Q}(\hat{G})$ are as follows:

$$\begin{aligned} \boldsymbol{R}(\boldsymbol{\psi}, \hat{G}) &= -\frac{1}{N} \sum_{\alpha=1}^{N} \frac{\partial \boldsymbol{\psi}(\boldsymbol{x}_\alpha|\boldsymbol{\theta})^T}{\partial \boldsymbol{\theta}} \bigg|_{\boldsymbol{\theta}=\boldsymbol{T}(\hat{G})} \\ &= -\frac{1}{N} \sum_{\alpha=1}^{N} \left\{ \frac{\partial^2 \log f(\boldsymbol{x}_\alpha|\boldsymbol{\theta})}{\partial \boldsymbol{\theta} \partial \boldsymbol{\theta}^T} \bigg|_{\boldsymbol{\theta}=\boldsymbol{T}(\hat{G})} - \frac{\partial^2}{\partial \boldsymbol{\theta} \partial \boldsymbol{\theta}^T} \left(\frac{1}{2} \boldsymbol{\theta}^T \boldsymbol{\mathcal{S}}_{\boldsymbol{\eta}}^{\mathcal{T}}(\boldsymbol{\theta}) \boldsymbol{\theta} \right) \bigg|_{\boldsymbol{\theta}=\boldsymbol{T}(\hat{G})} \right\} \\ &= -\frac{1}{N} \left\{ \frac{\partial^2}{\partial \boldsymbol{\theta} \partial \boldsymbol{\theta}^T} \ell(\boldsymbol{\theta}) \bigg|_{\boldsymbol{\theta}=\boldsymbol{T}(\hat{G})} - N \frac{\partial^2}{\partial \boldsymbol{\theta} \partial \boldsymbol{\theta}^T} \left(\frac{1}{2} \boldsymbol{\theta}^T \boldsymbol{\mathcal{S}}_{\boldsymbol{\eta}}^{\mathcal{T}}(\boldsymbol{\theta}) \boldsymbol{\theta} \right) \bigg|_{\boldsymbol{\theta}=\boldsymbol{T}(\hat{G})} \right\} \\ &= -\frac{1}{N} \left\{ \boldsymbol{\mathcal{H}}(\hat{\boldsymbol{\theta}}) - N \boldsymbol{\mathcal{S}}_{\boldsymbol{\eta}}^{\mathcal{T}}(\hat{\boldsymbol{\theta}}) \right\} = -\frac{1}{N} \boldsymbol{\mathcal{H}}_p(\hat{\boldsymbol{\theta}}), \\ \boldsymbol{Q}(\hat{G}) &= -\frac{1}{N} \sum_{\alpha=1}^{N} \frac{\partial^2 \log f(\boldsymbol{x}_\alpha|\boldsymbol{\theta})}{\partial \boldsymbol{\theta} \partial \boldsymbol{\theta}^T} \bigg|_{\boldsymbol{\theta}=\boldsymbol{T}(\hat{G})} = -\frac{1}{N} \frac{\partial^2 \ell(\boldsymbol{\theta})}{\partial \boldsymbol{\theta} \partial \boldsymbol{\theta}^T} \bigg|_{\boldsymbol{\theta}=\boldsymbol{T}(\hat{G})} = -\frac{1}{N} \boldsymbol{\mathcal{H}}(\hat{\boldsymbol{\theta}}). \end{aligned}$$

The estimated bias $b_1(\hat{G})$ is an estimate of the effective degrees of freedom (*edf*) of the penalized model, that is,

$$edf = b_1(\hat{G}) = \text{tr}\left\{\left[-\frac{1}{N}\boldsymbol{\mathcal{H}}_p(\hat{\boldsymbol{\theta}})\right]^{-1}\left[-\frac{1}{N}\boldsymbol{\mathcal{H}}(\hat{\boldsymbol{\theta}})\right]\right\} = \text{tr}\left\{\boldsymbol{\mathcal{H}}_p(\hat{\boldsymbol{\theta}})^{-1}\boldsymbol{\mathcal{H}}(\hat{\boldsymbol{\theta}})\right\}. \tag{C.10}$$

By substituting the asymptotic bias estimate in equation (C.10) into the expressions of the GIC (eq. C.5) and the GBIC (eq. C.6), the following generalized information criteria are obtained:

$$\begin{aligned} GIC(\boldsymbol{\mathcal{X}}_N; \hat{G}) &= -2N\left\{\frac{1}{N}\sum_{\alpha=1}^{N}\log f(\boldsymbol{x}_\alpha|\hat{\boldsymbol{\theta}}) - \frac{1}{N}b_1(\hat{G})\right\} \\ &= -2\sum_{\alpha=1}^{N}\log f(\boldsymbol{x}_\alpha|\hat{\boldsymbol{\theta}}) + 2\,\text{tr}\{\boldsymbol{R}(\boldsymbol{\psi}, \hat{G})^{-1}\boldsymbol{Q}(\hat{G})\} \\ &= -2\,\ell(\hat{\boldsymbol{\theta}}) + 2\,\text{tr}\left\{\boldsymbol{\mathcal{H}}_p(\hat{\boldsymbol{\theta}})^{-1}\boldsymbol{\mathcal{H}}(\hat{\boldsymbol{\theta}})\right\}, \end{aligned}$$

$$GBIC(\boldsymbol{\mathcal{X}}_N; \hat{G}) = -2\,\ell(\hat{\boldsymbol{\theta}}) + \log(N)\,\text{tr}\left\{\boldsymbol{\mathcal{H}}_p(\hat{\boldsymbol{\theta}})^{-1}\boldsymbol{\mathcal{H}}(\hat{\boldsymbol{\theta}})\right\}.$$

The vector of tuning parameters $\boldsymbol{\eta}$ enters through the penalty matrix, which is included in $\boldsymbol{\mathcal{H}}_p$. The determination of the tuning parameter(s) can be viewed as a model selection and evaluation problem. Therefore, information criteria evaluating a penalized model can be used as tuning parameter selectors. By evaluating statistical models determined according to grid(s) of values of $\boldsymbol{\eta}$, we take the optimal vector of the tuning parameter $\hat{\boldsymbol{\eta}}$ to be the one minimizing the value of the GBIC (since the BIC generally selects more sparse models than does the AIC), that is,

$$\hat{\boldsymbol{\eta}} = \arg\min_{\boldsymbol{\eta}} GBIC(\boldsymbol{\mathcal{X}}_N; \hat{G}).$$

Online Resource D Optimization and estimation

D.1 A general expression for the PMLE

To avoid notational clutter, we omit the superscript $\mathcal{T} = \{L, A, S, M\}$ in the expression of the penalty matrix. By using a first-order Taylor expansion of $\boldsymbol{g}_p(\boldsymbol{\theta}^{[t+1]})$ at $\boldsymbol{\theta}^{[t]}$ it follows that

$$\mathbf{0} = \boldsymbol{g}_p(\boldsymbol{\theta}^{[t+1]}) \approx \boldsymbol{g}_p(\boldsymbol{\theta}^{[t]}) + \boldsymbol{\mathcal{H}}_p(\boldsymbol{\theta}^{[t]})(\boldsymbol{\theta}^{[t+1]} - \boldsymbol{\theta}^{[t]}),$$

where $\boldsymbol{g}_p(\boldsymbol{\theta}^{[t]}) = \boldsymbol{g}(\boldsymbol{\theta}^{[t]}) - N\boldsymbol{\mathcal{S}}_{\hat{\eta}}(\tilde{\boldsymbol{\theta}}^{[t]})\boldsymbol{\theta}^{[t]}$ and $\boldsymbol{\mathcal{H}}_p(\boldsymbol{\theta}^{[t]}) = \boldsymbol{\mathcal{H}}(\boldsymbol{\theta}^{[t]}) - N\boldsymbol{\mathcal{S}}_{\hat{\eta}}(\tilde{\boldsymbol{\theta}}^{[t]})$. Define $\boldsymbol{\mathcal{I}}(\boldsymbol{\theta}^{[t]}) = -\boldsymbol{\mathcal{H}}(\boldsymbol{\theta}^{[t]})$, then

$$\mathbf{0} = \boldsymbol{g}_p(\boldsymbol{\theta}^{[t]}) + \left[-\boldsymbol{\mathcal{I}}(\boldsymbol{\theta}^{[t]}) - N\boldsymbol{\mathcal{S}}_{\hat{\eta}}(\tilde{\boldsymbol{\theta}}^{[t]})\right](\boldsymbol{\theta}^{[t+1]} - \boldsymbol{\theta}^{[t]}).$$

By rearranging the above equation, we get:

$$\begin{aligned}
\boldsymbol{g}_p(\boldsymbol{\theta}^{[t]}) &= \left[\boldsymbol{\mathcal{I}}(\boldsymbol{\theta}^{[t]}) + N\boldsymbol{\mathcal{S}}_{\hat{\eta}}(\tilde{\boldsymbol{\theta}}^{[t]})\right](\boldsymbol{\theta}^{[t+1]} - \boldsymbol{\theta}^{[t]}) \\
\boldsymbol{g}(\boldsymbol{\theta}^{[t]}) - N\boldsymbol{\mathcal{S}}_{\hat{\eta}}(\tilde{\boldsymbol{\theta}}^{[t]})\boldsymbol{\theta}^{[t]} &= \left[\boldsymbol{\mathcal{I}}(\boldsymbol{\theta}^{[t]}) + N\boldsymbol{\mathcal{S}}_{\hat{\eta}}(\tilde{\boldsymbol{\theta}}^{[t]})\right]\boldsymbol{\theta}^{[t+1]} - \boldsymbol{\mathcal{I}}(\boldsymbol{\theta}^{[t]})\boldsymbol{\theta}^{[t]} - N\boldsymbol{\mathcal{S}}_{\hat{\eta}}(\tilde{\boldsymbol{\theta}}^{[t]})\boldsymbol{\theta}^{[t]} \\
\boldsymbol{\theta}^{[t+1]}[\boldsymbol{\mathcal{I}}(\boldsymbol{\theta}^{[t]}) + N\boldsymbol{\mathcal{S}}_{\hat{\eta}}(\tilde{\boldsymbol{\theta}}^{[t]})] &= \boldsymbol{\mathcal{I}}(\boldsymbol{\theta}^{[t]})\boldsymbol{\theta}^{[t]} + \boldsymbol{g}(\boldsymbol{\theta}^{[t]}) \\
\boldsymbol{\theta}^{[t+1]}[\boldsymbol{\mathcal{I}}(\boldsymbol{\theta}^{[t]}) + N\boldsymbol{\mathcal{S}}_{\hat{\eta}}(\tilde{\boldsymbol{\theta}}^{[t]})] &= \sqrt{\boldsymbol{\mathcal{I}}(\boldsymbol{\theta}^{[t]})}\left[\sqrt{\boldsymbol{\mathcal{I}}(\boldsymbol{\theta}^{[t]})}\boldsymbol{\theta}^{[t]} + \sqrt{\boldsymbol{\mathcal{I}}(\boldsymbol{\theta}^{[t]})}^{-1}\boldsymbol{g}(\boldsymbol{\theta}^{[t]})\right].
\end{aligned}$$

Therefore, the vector parameter estimator can be expressed as

$$\boldsymbol{\theta}^{[t+1]} = \left[\boldsymbol{\mathcal{I}}(\boldsymbol{\theta}^{[t]}) + N\boldsymbol{\mathcal{S}}_{\hat{\eta}}(\tilde{\boldsymbol{\theta}}^{[t]})\right]^{-1}\sqrt{\boldsymbol{\mathcal{I}}(\boldsymbol{\theta}^{[t]})}\boldsymbol{K}^{[t]},$$

where $\boldsymbol{K}^{[t]} = \boldsymbol{\mu}_{\boldsymbol{K}}^{[t]} + \boldsymbol{\vartheta}^{[t]}$ with $\boldsymbol{\mu}_{\boldsymbol{K}}^{[t]} = \sqrt{\boldsymbol{\mathcal{I}}(\boldsymbol{\theta}^{[t]})}\boldsymbol{\theta}^{[t]}$ and $\boldsymbol{\vartheta}^{[t]} = \sqrt{\boldsymbol{\mathcal{I}}(\boldsymbol{\theta}^{[t]})}^{-1}\boldsymbol{g}(\boldsymbol{\theta}^{[t]})$. The square root of $\boldsymbol{\mathcal{I}}(\boldsymbol{\theta}^{[t]})$ and its inverse are obtained via eigenvalue decomposition (see Section D.2).

D.2 Correction for positive-definiteness

An eigenvalue decomposition is a technique that allows one to express an $m \times m$ symmetric matrix $\boldsymbol{B}$ as

$$\boldsymbol{B} = \boldsymbol{U}\boldsymbol{D}\boldsymbol{U}^T,$$

where $\boldsymbol{U}$ is an orthogonal matrix with the eigenvectors in its columns, and $\boldsymbol{D}$ is a diagonal matrix with the corresponding eigenvalues $d_{11}, \ldots, d_{qq}, \ldots, d_{mm}$ in the main diagonal, sorted in descending order. If all the eigenvalues are strictly positive, the matrix is said to be positive-definite, and its inverse is found as $\boldsymbol{B}^{-1} = \boldsymbol{U}\boldsymbol{D}^{-1}\boldsymbol{U}^T$.

However, if at least one of its eigenvalues is null or negative, the matrix is non-positive definite, and it must be corrected before its inversion takes place. An effective procedure that adjusts

the problematic eigenvalues of a non-positive definite matrix, and eventually makes the matrix positive-definite, is the following.

Without loss of generality, assume that all the eigenvalues of $\boldsymbol{B}$ are strictly positive except for the last one, i.e., $d_{qq} > 0$ for $q = 1, \dots, m-1$ and $d_{mm} \leq 0$. Define $l = \sum_{q=2}^{m} d_{qq}$ and $t = 100l^2 + 1$. The non-positive eigenvalue d_{mm} is then substituted with the positive quantity

$$\tilde{d}_{mm} = d_{m-1,m-1} \frac{(l - d_{mm})^2}{t},$$

where $d_{m-1,m-1}$ is the smallest positive eigenvalue of $\boldsymbol{B}$. By defining $\tilde{\boldsymbol{D}} = \text{diag}(d_{11}, \dots, d_{qq}, \dots, \tilde{d}_{mm})$, the corrected positive-definite matrix $\tilde{\boldsymbol{B}}$ can be found as

$$\tilde{\boldsymbol{B}} = \boldsymbol{U}\tilde{\boldsymbol{D}}\boldsymbol{U}^T,$$

and its inverse as

$$\tilde{\boldsymbol{B}}^{-1} = \boldsymbol{U}\tilde{\boldsymbol{D}}^{-1}\boldsymbol{U}^T.$$

We employed this procedure to compute and, if necessary, to correct the square root of $\boldsymbol{\mathcal{I}}(\boldsymbol{\theta})$ and its inverse.

D.3 Derivation of the UBRE criterion

Let $\boldsymbol{A}_\eta = \sqrt{\boldsymbol{\mathcal{I}}(\hat{\boldsymbol{\theta}})} \left[\boldsymbol{\mathcal{I}}(\hat{\boldsymbol{\theta}}) + N\boldsymbol{\mathcal{S}}_\eta(\hat{\boldsymbol{\theta}})\right]^{-1} \sqrt{\boldsymbol{\mathcal{I}}(\hat{\boldsymbol{\theta}})}$, where $\boldsymbol{A}_\eta$ is used as a shortcut for $\boldsymbol{A}_\eta^{\mathcal{T}}$ for $\mathcal{T} = \{L, A, S, M\}$. Based on the derivation in Section D.1, we can work out the expression of the UBRE criterion, i.e., the expectation of the average squared distance of $\hat{\boldsymbol{\mu}}_{\boldsymbol{K}} = \boldsymbol{A}_\eta \boldsymbol{K}$ from its expected value $\boldsymbol{\mu}_{\boldsymbol{K}}$:

$$\begin{aligned}
\mathbb{E}\left[\frac{1}{N}||\boldsymbol{\mu}_{\boldsymbol{K}} - \hat{\boldsymbol{\mu}}_{\boldsymbol{K}}||_2^2\right] &= \mathbb{E}\left[\frac{1}{N}||(\boldsymbol{K} - \boldsymbol{\vartheta}) - \boldsymbol{A}_\eta \boldsymbol{K}||_2^2\right] \\
&= \mathbb{E}\left[\frac{1}{N}||(\boldsymbol{K} - \boldsymbol{A}_\eta \boldsymbol{K}) - \boldsymbol{\vartheta}||_2^2\right] \\
&= \frac{1}{N}\mathbb{E}\left[||\boldsymbol{K} - \boldsymbol{A}_\eta \boldsymbol{K}||_2^2 + \boldsymbol{\vartheta}^T\boldsymbol{\vartheta} - 2\boldsymbol{\vartheta}^T(\boldsymbol{K} - \boldsymbol{A}_\eta \boldsymbol{K})\right] \\
&= \frac{1}{N}\mathbb{E}\left[||\boldsymbol{K} - \boldsymbol{A}_\eta \boldsymbol{K}||_2^2\right] + \frac{1}{N}\mathbb{E}\left[\boldsymbol{\vartheta}^T\boldsymbol{\vartheta}\right] - \frac{2}{N}\mathbb{E}\left[\boldsymbol{\vartheta}^T[\boldsymbol{\mu}_{\boldsymbol{K}} + \boldsymbol{\vartheta} - \boldsymbol{A}_\eta(\boldsymbol{\mu}_{\boldsymbol{K}} + \boldsymbol{\vartheta})]\right]
\end{aligned}$$

$$= \frac{1}{N}\mathbb{E}\left[||\boldsymbol{K} - \boldsymbol{A}_\eta \boldsymbol{K}||_2^2\right] - \frac{1}{N}\mathbb{E}\left[\boldsymbol{\vartheta}^T\boldsymbol{\vartheta}\right] - \frac{2}{N}\mathbb{E}\left[\boldsymbol{\vartheta}^T\boldsymbol{\mu}_{\boldsymbol{K}}\right]$$
$$+ \frac{2}{N}\mathbb{E}\left[\boldsymbol{\vartheta}^T\boldsymbol{A}_\eta\boldsymbol{\mu}_{\boldsymbol{K}}\right] + \frac{2}{N}\mathbb{E}\left[\boldsymbol{\vartheta}^T\boldsymbol{A}_\eta\boldsymbol{\vartheta}\right].$$

We now use the following results (Wood, 2017, Section 1.8.6)

$$\mathbb{E}\left[\boldsymbol{\vartheta}^T\boldsymbol{\vartheta}\right] = \mathbb{E}\left[\sum_{\alpha=1}^{N}\boldsymbol{\vartheta}_i^2\right] = N,$$

$$\mathbb{E}\left[\boldsymbol{\vartheta}^T\boldsymbol{\mu}_{\boldsymbol{K}}\right] = \mathbb{E}\left[\boldsymbol{\vartheta}^T\right]\boldsymbol{\mu}_{\boldsymbol{K}} = \boldsymbol{0},$$

$$\mathbb{E}\left[\boldsymbol{\vartheta}^T\boldsymbol{A}_\eta\boldsymbol{\mu}_{\boldsymbol{K}}\right] = \mathbb{E}\left[\boldsymbol{\vartheta}^T\right]\boldsymbol{A}_\eta\boldsymbol{\mu}_{\boldsymbol{K}} = \boldsymbol{0},$$

$$\mathbb{E}\left[\boldsymbol{\vartheta}^T\boldsymbol{A}_\eta\boldsymbol{\vartheta}\right] = \mathbb{E}\left[\mathrm{tr}\{\boldsymbol{\vartheta}^T\boldsymbol{A}_\eta\boldsymbol{\vartheta}\}\right] = \mathbb{E}\left[\mathrm{tr}\{\boldsymbol{A}_\eta\boldsymbol{\vartheta}\boldsymbol{\vartheta}^T\}\right] = \mathrm{tr}\{\mathbb{E}\left[\boldsymbol{A}_\eta\boldsymbol{\vartheta}\boldsymbol{\vartheta}^T\right]\}$$
$$= \mathrm{tr}\left\{\boldsymbol{A}_\eta\mathbb{E}\left[\boldsymbol{\vartheta}\boldsymbol{\vartheta}^T\right]\right\} = \mathrm{tr}\{\boldsymbol{A}_\eta\boldsymbol{I}\} = \mathrm{tr}(\boldsymbol{A}_\eta).$$

Then the expression of the UBRE criterion is:

$$\mathbb{E}\left[\frac{1}{N}||\boldsymbol{\mu}_{\boldsymbol{K}} - \hat{\boldsymbol{\mu}}_{\boldsymbol{K}}||_2^2\right] = \frac{1}{N}\mathbb{E}\left[||\boldsymbol{K} - \boldsymbol{A}_\eta\boldsymbol{K}||_2^2\right] + \frac{2}{N}\mathrm{tr}(\boldsymbol{A}_\eta) - 1.$$

D.4 Equivalence to the AIC

This section shows that $\mathcal{V}(\boldsymbol{\eta})$ is approximately proportional to the Akaike information criterion (AIC). The AIC of a model is defined as

$$\mathrm{AIC} := -2\ell(\boldsymbol{\theta}) + 2m,$$

where m is the number of estimated parameters in the model. Consider the following Taylor expansion of $-2\ell(\hat{\boldsymbol{\theta}})$ about $-2\ell(\boldsymbol{\theta})$:

$$\begin{aligned} -2\ell(\hat{\boldsymbol{\theta}}) &\approx -2\ell(\boldsymbol{\theta}) + (\hat{\boldsymbol{\theta}} - \boldsymbol{\theta})^T\nabla_{\boldsymbol{\theta}}[-2\ell(\boldsymbol{\theta})] + \frac{1}{2}(\hat{\boldsymbol{\theta}} - \boldsymbol{\theta})^T\nabla_{\boldsymbol{\theta}}\nabla_{\boldsymbol{\theta}^T}[-2\ell(\boldsymbol{\theta})](\hat{\boldsymbol{\theta}} - \boldsymbol{\theta}) \\ &\approx -2\ell(\boldsymbol{\theta}) - 2(\hat{\boldsymbol{\theta}} - \boldsymbol{\theta})^T\boldsymbol{g} - (\hat{\boldsymbol{\theta}} - \boldsymbol{\theta})^T\boldsymbol{\mathcal{H}}(\hat{\boldsymbol{\theta}} - \boldsymbol{\theta}), \end{aligned} \quad \text{(D.1)}$$

where we wrote $\boldsymbol{g} := \boldsymbol{g}(\boldsymbol{\theta})$ and $\boldsymbol{\mathcal{H}} := \boldsymbol{\mathcal{H}}(\boldsymbol{\theta})$ for simplicity of notation. By denoting $\boldsymbol{\mathcal{I}} = -\boldsymbol{\mathcal{H}}$ and recalling that $\boldsymbol{K} = \sqrt{\boldsymbol{\mathcal{I}}}\boldsymbol{\theta} + \sqrt{\boldsymbol{\mathcal{I}}}^{-1}\boldsymbol{g}$, we have that

$$\begin{aligned}
(\hat{\boldsymbol{\theta}} - \boldsymbol{\theta})^T\boldsymbol{g} &= (\hat{\boldsymbol{\theta}} - \boldsymbol{\theta})^T\sqrt{\boldsymbol{\mathcal{I}}}\sqrt{\boldsymbol{\mathcal{I}}}^{-1}\boldsymbol{g} = \left[\sqrt{\boldsymbol{\mathcal{I}}}(\hat{\boldsymbol{\theta}} - \boldsymbol{\theta})\right]^T\sqrt{\boldsymbol{\mathcal{I}}}^{-1}\boldsymbol{g} \\
&= \left[\sqrt{\boldsymbol{\mathcal{I}}}\hat{\boldsymbol{\theta}} - \sqrt{\boldsymbol{\mathcal{I}}}\boldsymbol{\theta}\right]^T\sqrt{\boldsymbol{\mathcal{I}}}^{-1}\boldsymbol{g} = \left[\sqrt{\boldsymbol{\mathcal{I}}}\hat{\boldsymbol{\theta}} - \boldsymbol{K} + \sqrt{\boldsymbol{\mathcal{I}}}^{-1}\boldsymbol{g}\right]^T\sqrt{\boldsymbol{\mathcal{I}}}^{-1}\boldsymbol{g} \\
&= -\left[\boldsymbol{K} - \sqrt{\boldsymbol{\mathcal{I}}}\hat{\boldsymbol{\theta}}\right]^T\sqrt{\boldsymbol{\mathcal{I}}}^{-1}\boldsymbol{g} + \boldsymbol{g}^T\sqrt{\boldsymbol{\mathcal{I}}}^{-1}\sqrt{\boldsymbol{\mathcal{I}}}^{-1}\boldsymbol{g} \\
&= -\left[\boldsymbol{K} - \sqrt{\boldsymbol{\mathcal{I}}}\hat{\boldsymbol{\theta}}\right]^T\sqrt{\boldsymbol{\mathcal{I}}}^{-1}\boldsymbol{g} + ||\sqrt{\boldsymbol{\mathcal{I}}}^{-1}\boldsymbol{g}||_2^2 \\
&= -\left\langle \boldsymbol{K} - \sqrt{\boldsymbol{\mathcal{I}}}\hat{\boldsymbol{\theta}}, \sqrt{\boldsymbol{\mathcal{I}}}^{-1}\boldsymbol{g}\right\rangle + ||\sqrt{\boldsymbol{\mathcal{I}}}^{-1}\boldsymbol{g}||_2^2, \qquad \text{(D.2)}
\end{aligned}$$

$$\begin{aligned}
-(\hat{\boldsymbol{\theta}} - \boldsymbol{\theta})^T\boldsymbol{\mathcal{H}}(\hat{\boldsymbol{\theta}} - \boldsymbol{\theta}) &= (\hat{\boldsymbol{\theta}} - \boldsymbol{\theta})^T\boldsymbol{\mathcal{I}}(\hat{\boldsymbol{\theta}} - \boldsymbol{\theta}) = ||\sqrt{\boldsymbol{\mathcal{I}}}(\hat{\boldsymbol{\theta}} - \boldsymbol{\theta})||_2^2 \\
&= ||\sqrt{\boldsymbol{\mathcal{I}}}\hat{\boldsymbol{\theta}} - \sqrt{\boldsymbol{\mathcal{I}}}\boldsymbol{\theta}||_2^2 = ||\sqrt{\boldsymbol{\mathcal{I}}}\hat{\boldsymbol{\theta}} - \boldsymbol{K} + \sqrt{\boldsymbol{\mathcal{I}}}^{-1}\boldsymbol{g}||_2^2 \\
&= ||\left(\boldsymbol{K} - \sqrt{\boldsymbol{\mathcal{I}}}\hat{\boldsymbol{\theta}}\right) - \sqrt{\boldsymbol{\mathcal{I}}}^{-1}\boldsymbol{g}||_2^2 \\
&= ||\boldsymbol{K} - \sqrt{\boldsymbol{\mathcal{I}}}\hat{\boldsymbol{\theta}}||_2^2 + ||\sqrt{\boldsymbol{\mathcal{I}}}^{-1}\boldsymbol{g}||_2^2 - 2\left\langle \boldsymbol{K} - \sqrt{\boldsymbol{\mathcal{I}}}\hat{\boldsymbol{\theta}}, \sqrt{\boldsymbol{\mathcal{I}}}^{-1}\boldsymbol{g}\right\rangle, \qquad \text{(D.3)}
\end{aligned}$$

where we used the fact that $||\boldsymbol{a}||_2^2 = ||-\boldsymbol{a}||_2^2$ for any vector $\boldsymbol{a}$, and $\langle\cdot,\cdot\rangle$ represents the inner product. By substituting equations (D.2) and (D.3) into expression (D.1), we obtain:

$$\begin{aligned}
-2\ell(\hat{\boldsymbol{\theta}}) \approx -2\ell(\boldsymbol{\theta}) &+ 2\left\langle \boldsymbol{K} - \sqrt{\boldsymbol{\mathcal{I}}}\hat{\boldsymbol{\theta}}, \sqrt{\boldsymbol{\mathcal{I}}}^{-1}\boldsymbol{g}\right\rangle - 2||\sqrt{\boldsymbol{\mathcal{I}}}^{-1}\boldsymbol{g}||_2^2 \\
&+ ||\boldsymbol{K} - \sqrt{\boldsymbol{\mathcal{I}}}\hat{\boldsymbol{\theta}}||_2^2 + ||\sqrt{\boldsymbol{\mathcal{I}}}^{-1}\boldsymbol{g}||_2^2 - 2\left\langle \boldsymbol{K} - \sqrt{\boldsymbol{\mathcal{I}}}\hat{\boldsymbol{\theta}}, \sqrt{\boldsymbol{\mathcal{I}}}^{-1}\boldsymbol{g}\right\rangle \\
= -2\ell(\boldsymbol{\theta}) &- ||\sqrt{\boldsymbol{\mathcal{I}}}^{-1}\boldsymbol{g}||_2^2 + ||\boldsymbol{K} - \sqrt{\boldsymbol{\mathcal{I}}}\hat{\boldsymbol{\theta}}||_2^2.
\end{aligned}$$

It then follows that

$$\begin{aligned}
\text{AIC} &= -2\ell(\boldsymbol{\theta}) + 2m \approx -2\ell(\boldsymbol{\theta}) - ||\sqrt{\boldsymbol{\mathcal{I}}}^{-1}\boldsymbol{g}||_2^2 + ||\boldsymbol{K} - \sqrt{\boldsymbol{\mathcal{I}}}\hat{\boldsymbol{\theta}}||_2^2 + 2m \\
&\approx -2\ell(\boldsymbol{\theta}) - ||\sqrt{\boldsymbol{\mathcal{I}}}^{-1}\boldsymbol{g}||_2^2 + ||\boldsymbol{K} - \sqrt{\boldsymbol{\mathcal{I}}}\hat{\boldsymbol{\theta}}||_2^2 + 2\text{tr}(\boldsymbol{A}_\eta), \qquad \text{(D.4)}
\end{aligned}$$

where tr$(\boldsymbol{A}_\eta)$ denotes the number of estimated parameters in the model, and thus, $m = \text{tr}(\boldsymbol{A}_\eta)$. Since we want to optimize the criterion with respect to the tuning parameter vector $\boldsymbol{\eta}$, we ignore any terms that are not affected by it, like $-2\ell(\boldsymbol{\theta})$ and $||\sqrt{\boldsymbol{\mathcal{I}}}^{-1}\boldsymbol{g}||_2^2$. After dropping these constants, expression (D.4) becomes proportional to the AIC, that is,

$$\text{AIC} = ||\boldsymbol{K} - \sqrt{\boldsymbol{\mathcal{I}}}\hat{\boldsymbol{\theta}}||_2^2 + 2\text{tr}(\boldsymbol{A}_\eta) \propto \mathcal{V}(\boldsymbol{\eta}),$$

where $||\boldsymbol{K} - \sqrt{\boldsymbol{\mathcal{I}}}\hat{\boldsymbol{\theta}}||_2^2$ is a quadratic approximation of $-2\ell(\hat{\boldsymbol{\theta}})$ and tr$(\boldsymbol{A}_\eta)$ represents the effective degrees of freedom of the model.

D.5 Intervals

At convergence, the covariance matrix of $\hat{\boldsymbol{\theta}}$ is $\boldsymbol{V}_{\hat{\boldsymbol{\theta}}} = \boldsymbol{\mathcal{J}}_p(\hat{\boldsymbol{\theta}})^{-1}\boldsymbol{\mathcal{J}}(\hat{\boldsymbol{\theta}})\boldsymbol{\mathcal{J}}_p(\hat{\boldsymbol{\theta}})^{-1}$. However, for practical purposes it is more convenient to employ at convergence the alternative Bayesian result $\boldsymbol{V}_{\boldsymbol{\theta}} = \boldsymbol{\mathcal{J}}_p(\hat{\boldsymbol{\theta}})^{-1}$. (For an unpenalized model $\boldsymbol{V}_{\hat{\boldsymbol{\theta}}}$ and $\boldsymbol{V}_{\boldsymbol{\theta}}$ are equivalent as there is no penalty involved in the covariance matrices.) In fact, at finite sample sizes, $\boldsymbol{V}_{\boldsymbol{\theta}}$ can produce intervals with close to nominal "across-the-function" frequentist coverage probabilities (Marra & Wood, 2012) because the Bayesian covariance matrix includes both a bias and variance component in a frequentist sense, a feature not shared by $\boldsymbol{V}_{\hat{\boldsymbol{\theta}}}$. This result can be justified using the distribution of $\boldsymbol{K}$ given in Section 7, making the large sample assumption that $\boldsymbol{\mathcal{H}}(\boldsymbol{\theta})$ can be treated as fixed, and making the prior Bayesian assumption of $\boldsymbol{\theta} \sim \mathcal{N}(\mathbf{0}, (N\boldsymbol{\mathcal{S}}_\eta(\tilde{\boldsymbol{\theta}}))^{-1})$.

The goodness of fit of the penalized model can then be evaluated through confidence intervals, which are available for each model parameter, obtained from the posterior distribution $\boldsymbol{\theta}|\{\boldsymbol{x}_1, \ldots, \boldsymbol{x}_N\}, \boldsymbol{\eta} \sim \mathcal{N}(\hat{\boldsymbol{\theta}}, \boldsymbol{V}_{\boldsymbol{\theta}})$. Confidence intervals for non-linear functions of the parameter vector $\boldsymbol{\theta}$ can be conveniently obtained by simulation from the posterior of $\boldsymbol{\theta}$ as follows. Let $T(\boldsymbol{\theta})$ be any function of the parameters, then

Step 1 Draw N_{sim} random vectors $\boldsymbol{\theta}_h^\star$ (for $h = 1, \ldots, N_{\text{sim}}$) from $\mathcal{N}(\hat{\boldsymbol{\theta}}, \boldsymbol{V}_{\boldsymbol{\theta}})$;

Step 2 Compute $T_h^\star := T(\boldsymbol{\theta}_h^\star)\,\forall\, h$, and define $T_\alpha^\star$ to be the $[N_{\text{sim}} \cdot \alpha]^{\text{th}}$ smallest value of the ordered sample $\{T_1^\star, \ldots, T_{N_{\text{sim}}}^\star\}$, with $[a]$ denoting the integer part of $a \in \mathbb{R}$;

Step 3 Obtain an approximate $(1-\alpha)\%$ confidence interval for $T(\hat{\boldsymbol{\theta}})$ using $\left[T_{\frac{\alpha}{2}}^\star, T_{1-\frac{\alpha}{2}}^\star\right]$, where α is

usually set to 0.05.

Introducing penalties in the estimation process is fundamentally motivated by the belief that in the population, the factor structures are more likely to be sparse than dense, and similar across sub-populations rather than heterogeneous. This prior belief can be formalized by specifying the exponential prior exp $\left\{-\frac{N}{2}\boldsymbol{\theta}^T\boldsymbol{\mathcal{S}}_\eta(\tilde{\boldsymbol{\theta}})\boldsymbol{\theta}\right\}$ on the penalty function. This is equivalent to assuming for the parameter vector a zero-mean improper Gaussian prior distribution with precision matrix proportional to $\boldsymbol{\mathcal{S}}_\eta(\tilde{\boldsymbol{\theta}})$, i.e., $\boldsymbol{\theta} \propto \mathcal{N}(\mathbf{0}, (N\boldsymbol{\mathcal{S}}_\eta(\tilde{\boldsymbol{\theta}}))^{-1})$, where $\boldsymbol{\mathcal{S}}_\eta(\tilde{\boldsymbol{\theta}})^{-1}$ is the Moore-Penrose pseudo-inverse of $\boldsymbol{\mathcal{S}}_\eta(\tilde{\boldsymbol{\theta}})$ (Wood, 2017). The proposed penalized approach can thus be viewed as an "empirical Bayes" method that gives good frequentist properties.

Online Resource E Empirical application

The Holzinger & Swineford data set (Holzinger & Swineford, 1939; Kelley, 2019) includes the following 19 tests: visual perception (VISUAL), cubes (CUBES), paper from board (PAPER), flags (FLAGS), general information (GENERAL), paragraph comprehension (PARAGRAP), sentence completion (SENTENCE), word classification (WORDC), word meaning (WORDM), addition (ADDITION), code (CODE), counting groups of dots (COUNTING), straight and curved capitals (STRAIGHT), word recognition (WORDR), number recognition (NUMBERR), figure recognition (FIGURER), object-number (OBJECT), number-figure (NUMBERF), figure-word (FIGUREW). These tests are thought of as measuring four correlated abilities: spatial ability (VISUAL, CUBES, PAPER, FLAGS), verbal intelligence (GENERAL, PARAGRAP, SENTENCE, WORDC, WORDM), speed (ADDITION, CODE, COUNTING, STRAIGHT), and memory (WORDR, NUMBERR, FIGURER, OBJECT, NUMBERF, FIGUREW).

The parameter estimates of the best `lslx` (BIC = 7565.92; mcp, $\hat{\eta} = 0.13, \hat{a} = 3.32$) and `regsem` (BIC = 7565.21; mcp, $\hat{\eta} = 1.28, a = 3.7$) models are illustrated in Table E.3 for the single-group analysis. Fixed parameters are italic and underlined. A blank cell indicates that the corresponding estimate is zero. Two penalized cross-loadings ($\hat{\lambda}_{91}, \hat{\lambda}_{32}$) were identified as non-zero by both methods. Additionally, `lslx` detected another secondary loading ($\hat{\lambda}_{51}$). Table E.4 reports the parameter estimates for the optimal `lslx` model (BIC = 14697.72; mcp, $\hat{\eta} = 0.14, \hat{a} = 3$) in the multiple-group analysis. Non-invariant parameters across groups are starred (*).

**Measurement model**	`lslx-mcp`				`regsem-mcp`			
	Spatial	Verbal	Speed	Ψ	Spatial	Verbal	Speed	Ψ
VISUAL	0.85	*0*	*0*	0.62	0.84	*0*	*0*	0.66
CUBES	0.52			1.11	0.52			1.11
FLAGS	0.80	−0.17		0.73	0.86	−0.26		0.70
PARAGRAP	*0*	0.98	*0*	0.38	*0*	0.99	*0*	0.37
SENTENCE	−0.12	1.17		0.40		1.11		0.44
WORDM		0.91		0.36		0.91		0.36
ADDITION	*0*	*0*	0.66	0.74	*0*	*0*	0.66	0.75
COUNTING			0.81	0.37			0.81	0.36
STRAIGHT	0.37		0.45	0.57	0.38		0.44	0.57
**Structural model**	Spatial	Verbal	Speed		Spatial	Verbal	Speed	
Spatial	*1*	0.51	0.31		*1*	0.52	0.31	
Verbal	-	*1*	0.21		-	*1*	0.20	
Speed	-	-	*1*		-	-	*1*	

Table E.3: Parameter estimates of the nine mental tests from the Holzinger & Swineford data set for `lslx-mcp` ($\hat{\eta} = 0.13, \hat{a} = 3.32$) and `regsem-mcp` ($\hat{\eta} = 1.28, a = 3.7$).

	Pasteur School						Grant-White School					
**Measurement model**	$\boldsymbol{\tau}_1$	Spatial	Verbal	Speed	Memory	Ψ_1	$\boldsymbol{\tau}_2$	Spatial	Verbal	Speed	Memory	Ψ_2
VISUAL	*0*	*1*	*0*	*0*	*0*	0.48	*0*	*1*	*0*	*0*	*0*	0.45
CUBES	0.01	0.64				0.87	0.01	0.64				0.67
PAPER	0.00	0.66				0.81	0.00	0.66				0.71
FLAGS	$0.27^\star$	0.86				0.62	$-0.28^\star$	0.86				0.47
GENERAL	$-0.03^\star$		1.03		−0.15	0.26	$0.02^\star$		1.03		−0.15	0.31
PARAGRAP	$0.00^\star$		0.98			0.35	$-0.01^\star$		0.98			0.31
SENTENCE	0.00	−0.09	1.10		−0.10	0.25	0.00	−0.09	1.10		−0.10	0.21
WORDC	$-0.09^\star$	0.04	0.84			0.40	$0.09^\star$	0.04	0.84			0.44
WORDM	*0*	*0*	*1*	*0*	*0*	0.23	*0*	*0*	*1*	*0*	*0*	0.35
ADDITION	−0.02	−0.47		1.31		0.48	−0.02	−0.47		1.31		0.30
CODE	−0.01	0	0.15	0.91	0.10	0.43	−0.01		0.15	0.91	0.10	0.63
COUNTING	*0*	*0*	*0*	*1*	*0*	0.61	*0*	*0*	*0*	*1*	*0*	0.52
STRAIGHT	0.00	0.37		0.76		0.63	0.00	0.37		0.76		0.46
WORDR	*0*	*0*	*0*	*0*	*1*	0.61	*0*	*0*	*0*	*0*	*1*	0.57
NUMBERR	0.00		−0.09		0.88	0.69	0.00		−0.09		0.88	0.68
FIGURER	0.01	0.40			0.69	0.72	0.01	0.40			0.69	0.46
OBJECT	$0.12^\star$	−0.32		0.46	0.90	0.60	$-0.15^\star$	−0.32		0.46	0.90	0.45
NUMBERF	0.03				0.68	0.77	0.03			$0.49^\star$	0.68	0.62
FIGUREW	$-0.28^\star$			0.17	0.58	0.83	$0.29^\star$			0.17	0.58	0.60
**Structural model**	$\boldsymbol{\kappa}_1$	Spatial	Verbal	Speed	Memory		$\boldsymbol{\kappa}_2$	Spatial	Verbal	Speed	Memory	
Spatial	−0.07	0.54	0.25	0.17	0.16		0.07	0.55	0.32	0.29	0.21	
Verbal	−0.25		0.64	0.21	0.11		0.28		0.62	0.26	0.24	
Speed	0.12			0.36	0.12		−0.11			0.50	0.16	
Memory	−0.04				0.48		0.04				0.37	

Table E.4: Parameter estimates of the 19 mental tests from the Holzinger & Swineford data set for `lslx-mcp` ($\hat{\eta} = 0.14, \hat{a} = 3$).

Online Resource F Software implementation

The proposed methodology and estimation approach are implemented in the R package penfa to enhance reproducible research and transparent dissemination of results. In this section, we describe the main functions for fitting single and multiple-group factor analysis models according to the penalized likelihood-based estimation framework proposed in this paper. To this end, we demonstrate how users can carry out the empirical analyses presented in Sections 9.1 and 9.2. The subsequent analyses require the R package penfa to be installed and loaded.

F.1 Penalized factor analysis

The empirical analysis presented in Section 9 employs the Holzinger & Swineford data set (Holzinger & Swineford, 1939), a classical psychometric application on students' mental abilities. The data set, already scaled as described in Yuan and Bentler (2006), is contained in the R package lavaan (Rosseel, 2012; Rosseel et al., 2019). Let us load and inspect the data.

```
data <- lavaan::HolzingerSwineford1939
summary(data)

##        id             sex            ageyr        agemo
##  Min.   :  1.0   Min.   :1.000   Min.   :11   Min.   : 0.000
##  1st Qu.: 82.0   1st Qu.:1.000   1st Qu.:12   1st Qu.: 2.000
##  Median :163.0   Median :2.000   Median :13   Median : 5.000
##  Mean   :176.6   Mean   :1.515   Mean   :13   Mean   : 5.375
##  3rd Qu.:272.0   3rd Qu.:2.000   3rd Qu.:14   3rd Qu.: 8.000
##  Max.   :351.0   Max.   :2.000   Max.   :16   Max.   :11.000
##
##         school        grade             x1               x2
##  Grant-White:145   Min.   :7.000   Min.   :0.6667   Min.   :2.250
##  Pasteur    :156   1st Qu.:7.000   1st Qu.:4.1667   1st Qu.:5.250
##                    Median :7.000   Median :5.0000   Median :6.000
##                    Mean   :7.477   Mean   :4.9358   Mean   :6.088
##                    3rd Qu.:8.000   3rd Qu.:5.6667   3rd Qu.:6.750
##                    Max.   :8.000   Max.   :8.5000   Max.   :9.250
##                    NA's   :1
##        x3              x4              x5              x6
##  Min.   :0.250   Min.   :0.000   Min.   :1.000   Min.   :0.1429
##  1st Qu.:1.375   1st Qu.:2.333   1st Qu.:3.500   1st Qu.:1.4286
##  Median :2.125   Median :3.000   Median :4.500   Median :2.0000
```

```
##  Mean   :2.250   Mean   :3.061   Mean   :4.341   Mean   :2.1856
##  3rd Qu.:3.125   3rd Qu.:3.667   3rd Qu.:5.250   3rd Qu.:2.7143
##  Max.   :4.500   Max.   :6.333   Max.   :7.000   Max.   :6.1429
##
##        x7               x8               x9
##  Min.   :1.304   Min.   : 3.050   Min.   :2.778
##  1st Qu.:3.478   1st Qu.: 4.850   1st Qu.:4.750
##  Median :4.087   Median : 5.500   Median :5.417
##  Mean   :4.186   Mean   : 5.527   Mean   :5.374
##  3rd Qu.:4.913   3rd Qu.: 6.100   3rd Qu.:6.083
##  Max.   :7.435   Max.   :10.000   Max.   :9.250
##
```

The data set contains information on the test scores (items x_1 to x_9) of $N = 301$ seventh-grade and eighth-grade students on $p = 9$ mental tests. Additional information is available, such as the age of the students and the attended school (i.e., Pasteur or Grant-White). Let us select and center the data subset constituted by the nine tests.

```
data <- scale(data[,7:15], center = TRUE, scale = FALSE)
```

The following sections describe how to specify and estimate a penalized factor analysis model using the adaptive lasso penalization to encourage a sparse factor loading matrix and the automatic tuning parameter procedure to select the optimal amount of sparsity. This combination of penalty and tuning selection strategy produced the model with the superior fit in the empirical analysis (see Table 4 with the BIC ranking).

F.1.1 Model specification

Before fitting the model, users should write a "model syntax" which describes the model to be estimated and specifies the relationships between the observed variables and the latent variables (i.e., the common factors). To facilitate its formulation, the rules for the syntax specification broadly follow the ones required by the package `lavaan`. Let us have a look at the following syntax, which is enclosed in single quotes.

```
syntax <- '
# Measurement model
spatial =~   x1 + x2 + x3 + 0*x4 + x5 + x6 + 0*x7 + x8 + x9
```

```
verbal   =~ 0*x1 + x2 + x3 +   x4 + x5 + x6 + 0*x7 + x8 + x9
speed    =~ 0*x1 + x2 + x3 + 0*x4 + x5 + x6 +   x7 + x8 + x9

# Unit variances for common factors
spatial ~~ 1*spatial
verbal  ~~ 1*verbal
speed   ~~ 1*speed
'
```

The three common factors are referred to as `spatial`, `verbal` and `speed`, whereas the observed variables names range from `x1` to `x9`. The factors appear on the left-hand side, whereas the observed variables on the right-hand side. The special operator "=~" is read as "is measured by", and is used to list the observed variables loading on each factor. The factor variances and covariances are specified using the double tilde operator "~~". In order to fix a parameter to a given value, we pre-multiply (through the symbol "*") the corresponding variable in the formula by the specific numerical value.

The above syntax specifies a factor model with $r = 3$ common factors, where each observed variable loads on each of the factors, apart from the ones whose loadings are fixed to zero for identification purposes. The scales of the factors are specified by fixing their variances to 1.0. By default, the unique variances are automatically added to the model, and the common factors are allowed to correlate. These specifications can be easily modified by altering the syntax according to one's own preferences.

F.1.2 Model fitting

We now show how to estimate the factor analysis model specified in the syntax according to the penalized likelihood-based approach presented in this work. The estimation process is demonstrated for the alasso penalty and the automatic tuning procedure, but the rationale is similar for other choices of penalty functions. The alasso employs a set of adaptive weights correcting the bias issue of the lasso. A common choice for the weights is given by the maximum likelihood estimates from the unpenalized factor model. The unpenalized model can be estimated through the function `penfa` as follows:

```
fit.mle <- penfa(model = syntax,
                 data  = data,
                 information = "fisher",
                 pen.shrink = "none",
                 pen.diff = "none",
                 eta = list(shrink = c("none" = 0),
                            diff   = c("none" = 0)),
                 strategy = "fixed",
                 verbose = FALSE)
```

The function `penfa` takes as first argument the user-specified model syntax, and as second argument the data set with the observed variables. The `information` argument allows users to choose between the penalized expected Fisher information ("fisher") or the penalized Hessian matrix ("hessian") as second-order derivatives to be used in the trust-region algorithm. In the `pen.shrink` and `pen.diff` arguments, users can specify the penalty functions for sparsity and parameter equivalence (for multiple-group analyses); when they are both set equal to "none", no penalization is applied, and the model is estimated by ordinary maximum likelihood. We specify `strategy` equal to "fixed" to prompt an analysis using as tuning values the ones defined in the `eta` argument. We can get an overview of the data set and the optimization process by printing the `fit.mle` object.

```
fit.mle

## penfa reached convergence
##
##   Number of observations                                   301
##
##   Estimator                                                MLE
##   Optimization method                             trust-region
##   Information                                           fisher
##   Strategy                                               fixed
##   Number of iterations                                      15
##   Effective degrees of freedom                          33.000
##
```

The trust-region algorithm required a small number of iterations to converge. Since no penalization is imposed, the effective degrees of freedom coincide with the number of model parameters, that is, $edf = m = 33$. The parameter estimates can be extracted through the function `coef` together with their names. Each name is composed of three parts and reflects the part of the

formula in which a given parameter is involved. The variable name appears on the left-hand side of the formula, the operator is placed in the middle, and the variable corresponding to the parameter is on the right-hand side.

```
weights <- coef(fit.mle)
weights

##     spatial=~x1     spatial=~x2     spatial=~x3     spatial=~x5
##           0.814           0.652           0.909          -0.134
##     spatial=~x6     spatial=~x8     spatial=~x9      verbal=~x2
##           0.067           0.296           0.540          -0.118
##      verbal=~x3      verbal=~x4      verbal=~x5      verbal=~x6
##          -0.330           0.987           1.193           0.875
##      verbal=~x8      verbal=~x9       speed=~x2       speed=~x3
##          -0.158          -0.141          -0.161          -0.012
##       speed=~x5       speed=~x6       speed=~x7       speed=~x8
##           0.008          -0.020           0.767           0.680
##       speed=~x9          x1~~x1          x2~~x2          x3~~x3
##           0.433           0.696           1.035           0.692
##          x4~~x4          x5~~x5          x6~~x6          x7~~x7
##           0.377           0.403           0.365           0.594
##          x8~~x8          x9~~x9 spatial~~verbal  spatial~~speed
##           0.479           0.551           0.585           0.173
##   verbal~~speed
##           0.220
```

The estimation of the penalized factor model is again carried out through the `penfa` function, but with some new arguments. The alasso penalty is specified in the `pen.shrink` argument (`pen.diff` is still equal to "none"), whereas the adaptive weights are given in the `weights` argument. The value of the additional tuning parameter a of the alasso can be assigned through the `a.alasso` argument, whereas the `eta` argument allows users to provide a starting value for the shrinkage parameter η. The name given to the starting value - "lambda" in this case - reflects the parameter matrix or vector to be penalized. By default, all of its elements are penalized, which means here that the penalization is applied to all of the factor loadings. If "strategy" is specified equal to "fixed", then a penalized model with the value of η given in `eta` is estimated, whereas the automatic tuning parameter procedure is carried out when `strategy` is set equal to "auto". Lastly, users can choose a specific value of the influence factor γ through the `gamma` argument.

```r
fit <- penfa(## factor model
             model = syntax,
             data  = data,
             information = "fisher",
             # penalization
             pen.shrink = "alasso",
             pen.diff = "none",
             eta = list(shrink = c("lambda" = 0.01),
                        diff = c("none" = 0)),
             # automatic procedure
             strategy = "auto",
             gamma = 4.5,
             # alasso
             a.alasso = 1,
             weights = weights,
             verbose = FALSE)
fit

## penfa reached convergence
##
##   Number of observations                                301
##
##   Estimator                                            PMLE
##   Optimization method                          trust-region
##   Information                                        fisher
##   Strategy                                             auto
##   Number of iterations (total)                           32
##   Number of two-steps (automatic)                         1
##   Effective degrees of freedom                       22.843
##
##   Penalty function:
##     Sparsity                                         alasso
##
```

Printing the fitted object gives an overview of the optimization and penalization processes, including the employed optimizer and penalty function, the total number of iterations and the number of outer iterations of the automatic procedure. The automatic procedure is very fast, as it required a single outer iteration to reach convergence. The number of effective degrees of freedom of the penalized model is $edf = 22.843$, which is a fractional number, as opposed to the integer number that existing penalized factor analytic techniques report for the degrees of freedom.

The `summary` function details information on the model characteristics, the optimization and the penalization procedures, as well as the parameter estimates with associated standard errors and

confidence intervals. The optimal value of the tuning parameter is $\hat{\eta} = 0.017$. The data set well supported the introduction of sparsity, as demonstrated by the reduction in the Generalized Bayesian Information Criterion (GBIC) when moving from the unpenalized model `fit.mle` (7601.416) to its penalized counterpart `fit` (7558.026). The *Type* column distinguishes between the *fixed* parameters that have been set to specific values for identification purposes, the *free* parameters that have been estimated through ordinary maximum likelihood, and the penalized parameters (denoted as *pen*). The standard errors are computed as the square root of the inverse of the penalized Fisher information matrix (or alternatively, of the penalized Hessian if `information` = "hessian"). The last columns report 95% confidence intervals for the model parameters. The standard errors and the confidence intervals of the penalized parameters that were shrunken to zero are not reported. A different significance level can be specified through the `level` argument in the `summary` call.

```
summary(fit)

## penfa reached convergence
##
##   Number of observations                                   301
##   Number of groups                                           1
##   Number of observed variables                               9
##   Number of latent factors                                   3
##
##   Estimator                                               PMLE
##   Optimization method                             trust-region
##   Information                                           fisher
##   Strategy                                                auto
##   Number of iterations (total)                              32
##   Number of two-steps (automatic)                            1
##   Influence factor                                         4.5
##   Number of parameters:
##     Free                                                    12
##     Penalized                                               21
##   Effective degrees of freedom                          22.843
##   GIC                                                 7473.346
##   GBIC                                                7558.026
##
##   Penalty function:
##     Sparsity                                            alasso
##
##   Additional tuning parameter
##     alasso                                                   1
##
##   Optimal tuning parameter:
##     Sparsity
```

```
##      - Factor loadings                                     0.017
##
## Parameter Estimates:
##
## Latent Variables:
##                  Type    Estimate    Std.Err       2.5%      97.5%
##   spatial =~
##     x1           pen        0.829      0.073      0.685      0.972
##     x2           pen        0.493      0.073      0.350      0.636
##     x3           pen        0.758      0.086      0.591      0.926
##     x4           fixed      0.000                 0.000      0.000
##     x5           pen       -0.060      0.034     -0.128      0.007
##     x6           pen        0.000
##     x7           fixed      0.000                 0.000      0.000
##     x8           pen        0.124      0.059      0.008      0.239
##     x9           pen        0.410      0.062      0.290      0.531
##   verbal =~
##     x1           fixed      0.000                 0.000      0.000
##     x2           pen       -0.000
##     x3           pen       -0.157      0.066     -0.286     -0.029
##     x4           pen        0.960      0.055      0.852      1.069
##     x5           pen        1.114      0.065      0.987      1.240
##     x6           pen        0.889      0.052      0.787      0.992
##     x7           fixed      0.000                 0.000      0.000
##     x8           pen       -0.000
##     x9           pen       -0.000
##   speed =~
##     x1           fixed      0.000                 0.000      0.000
##     x2           pen       -0.013
##     x3           pen        0.000
##     x4           fixed      0.000                 0.000      0.000
##     x5           pen        0.000
##     x6           pen        0.000
##     x7           pen        0.697      0.078      0.544      0.850
##     x8           pen        0.704      0.077      0.553      0.854
##     x9           pen        0.423      0.060      0.305      0.541
##
## Covariances:
##                  Type    Estimate    Std.Err       2.5%      97.5%
##   spatial ~~
##     verbal       free       0.481      0.065      0.354      0.609
##     speed        free       0.196      0.098      0.004      0.389
##   verbal ~~
##     speed        free       0.160      0.077      0.008      0.312
##
## Variances:
##                  Type    Estimate    Std.Err       2.5%      97.5%
##     spatial      fixed      1.000                 1.000      1.000
##     verbal       fixed      1.000                 1.000      1.000
##     speed        fixed      1.000                 1.000      1.000
##    .x1           free       0.623      0.095      0.438      0.809
```

```
##     .x2           free        1.110       0.099       0.917       1.304
##     .x3           free        0.748       0.092       0.567       0.930
##     .x4           free        0.380       0.048       0.287       0.473
##     .x5           free        0.418       0.059       0.303       0.533
##     .x6           free        0.363       0.043       0.279       0.447
##     .x7           free        0.669       0.097       0.479       0.859
##     .x8           free        0.444       0.087       0.273       0.616
##     .x9           free        0.560       0.059       0.444       0.676
```

The penalty matrix $\mathcal{S}_{\hat{\eta}}(\hat{\boldsymbol{\theta}})$ at convergence is stored in the slot `@Penalize`. It is a diagonal matrix with the elements on the diagonal quantifying the extent to which each model parameter has been penalized.

```
round(diag(fit@Penalize@Sh.info$S.h), 2)

##     spatial=~x1     spatial=~x2     spatial=~x3     spatial=~x5
##            7.64           16.02            7.47          639.57
##     spatial=~x6     spatial=~x8     spatial=~x9      verbal=~x2
##       626389.20          140.69           23.27       427303.89
##      verbal=~x3      verbal=~x4      verbal=~x5      verbal=~x6
##           99.47            5.44            3.88            6.62
##      verbal=~x8      verbal=~x9       speed=~x2       speed=~x3
##       246589.16       347789.43         2446.04      4332622.32
##       speed=~x5       speed=~x6       speed=~x7       speed=~x8
##      6419433.77      2587290.17            9.63           10.76
##       speed=~x9          x1~~x1          x2~~x2          x3~~x3
##           28.16            0.00            0.00            0.00
##          x4~~x4          x5~~x5          x6~~x6          x7~~x7
##            0.00            0.00            0.00            0.00
##          x8~~x8          x9~~x9 spatial~~verbal  spatial~~speed
##            0.00            0.00            0.00            0.00
##   verbal~~speed
##            0.00
```

The values corresponding to the factor loadings are different from zero, as these are the parameters that have been penalized, whereas the values for the unique variances (`x1~~x1` to `x9~~x9`) and the factor covariances (`spatial~~verbal`, `spatial~~speed`, `verbal~~speed`) are zero, as these elements were not affected by the penalization. The magnitude of the penalization varied depending on the size of the factor loading to be penalized: small loadings received a considerable penalty, whereas large loadings a little one. Figure F.3 shows the heat map of the penalty matrix $\mathcal{S}^{A}_{\hat{\eta}}(\hat{\boldsymbol{\theta}})$ on a log-scale, given the wide range of its elements (from 0 to over 6×10^6).

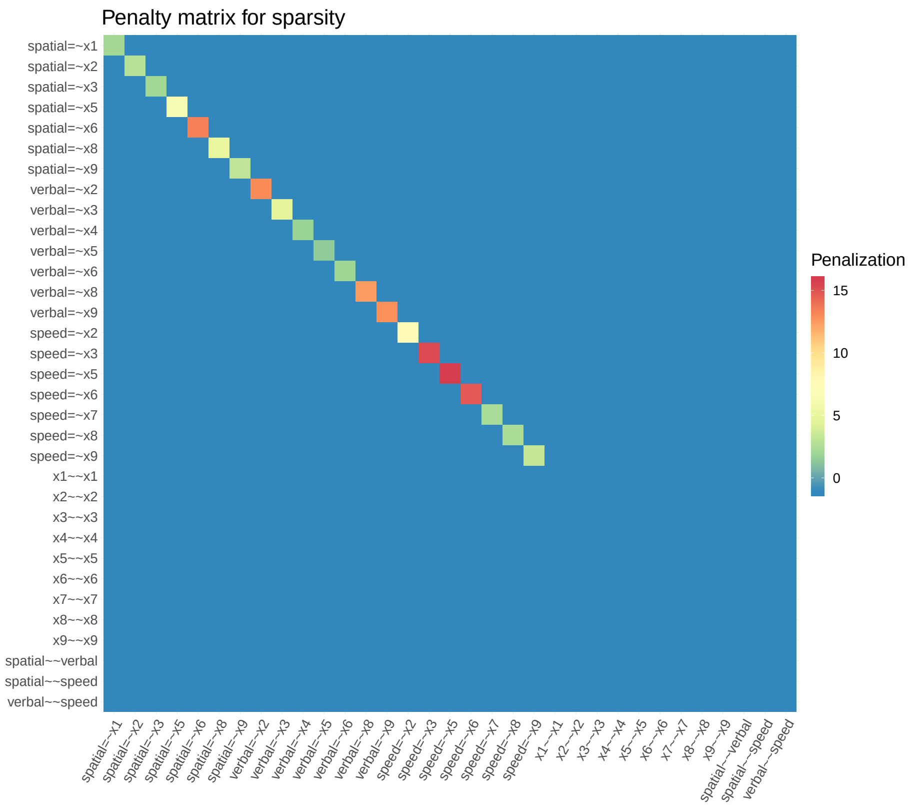

Figure F.3: Heat map of the penalty matrix $\mathcal{S}_{\hat{\eta}}^{A}(\hat{\boldsymbol{\theta}})$ on a log-scale for `penfa-alasso` ($a = 1, \gamma = 4.5$) on the Holzinger & Swineford data set.

F.2 Penalized multiple-group factor analysis

As a followup, we consider the penalized estimation of a multiple-group factor model with the alasso penalty and the automatic multiple tuning procedure (Section 9.2). Interestingly, there are now multiple tuning parameters: one of them introduces sparsity in the factor loading matrices of each group, whereas the other two encourage cross-group invariance of loadings and intercepts. For this example, we use the complete version of the Holzinger & Swineford data set in the `R` package `MBESS` (Kelley, 2019). An inspection at the data set reveals that it contains the scores on 26 tests from $N = 301$ students attending the Pasteur and Grant-White schools. We analyze the subset consisting of the first $p = 19$ tests, which we standardized to handle the scaling effect. The variables were also renamed for convenience when formulating the syntax.

```
library(MBESS)
data(HS)
data <- HS[,8:27]
colnames(data) <- c("school","visual","cubes","paper","flags", "general",
```

```r
                     "paragrap", "sentence", "wordc", "wordm", "addition",
                     "code", "counting", "straight", "wordr", "numberr",
                     "figurer", "object", "numberf", "figurew")
summary(data)
```

```
##          school         visual           cubes            paper      
##  Grant-White:145   Min.   : 4.00   Min.   : 9.00   Min.   : 6.00  
##  Pasteur    :156   1st Qu.:25.00   1st Qu.:21.00   1st Qu.:12.00  
##                    Median :30.00   Median :24.00   Median :14.00  
##                    Mean   :29.61   Mean   :24.35   Mean   :14.23  
##                    3rd Qu.:34.00   3rd Qu.:27.00   3rd Qu.:16.00  
##                    Max.   :51.00   Max.   :37.00   Max.   :25.00  
##      flags       general          paragrap         sentence    
##  Min.   : 2   Min.   : 8.00   Min.   : 0.000   Min.   : 4.00  
##  1st Qu.:11   1st Qu.:31.00   1st Qu.: 7.000   1st Qu.:14.00  
##  Median :17   Median :41.00   Median : 9.000   Median :18.00  
##  Mean   :18   Mean   :40.62   Mean   : 9.183   Mean   :17.36  
##  3rd Qu.:25   3rd Qu.:49.00   3rd Qu.:11.000   3rd Qu.:21.00  
##  Max.   :36   Max.   :84.00   Max.   :19.000   Max.   :28.00  
##      wordc           wordm          addition           code       
##  Min.   :10.00   Min.   : 1.0   Min.   : 30.00   Min.   : 19.00  
##  1st Qu.:23.00   1st Qu.:10.0   1st Qu.: 80.00   1st Qu.: 60.00  
##  Median :26.00   Median :14.0   Median : 94.00   Median : 68.00  
##  Mean   :26.13   Mean   :15.3   Mean   : 96.24   Mean   : 69.16  
##  3rd Qu.:30.00   3rd Qu.:19.0   3rd Qu.:113.00   3rd Qu.: 79.00  
##  Max.   :43.00   Max.   :43.0   Max.   :171.00   Max.   :118.00  
##     counting        straight         wordr          numberr   
##  Min.   : 61.0   Min.   :100.0   Min.   :121.0   Min.   : 68  
##  1st Qu.: 97.0   1st Qu.:171.0   1st Qu.:168.0   1st Qu.: 84  
##  Median :110.0   Median :195.0   Median :176.0   Median : 90  
##  Mean   :110.5   Mean   :193.4   Mean   :175.2   Mean   : 90  
##  3rd Qu.:122.0   3rd Qu.:219.0   3rd Qu.:184.0   3rd Qu.: 96  
##  Max.   :200.0   Max.   :333.0   Max.   :198.0   Max.   :112  
##     figurer          object          numberf          figurew     
##  Min.   : 58.0   Min.   : 0.000   Min.   : 0.000   Min.   : 3.00  
##  1st Qu.: 98.0   1st Qu.: 5.000   1st Qu.: 6.000   1st Qu.:11.00  
##  Median :103.0   Median : 8.000   Median : 9.000   Median :14.00  
##  Mean   :102.5   Mean   : 8.216   Mean   : 9.395   Mean   :14.02  
##  3rd Qu.:107.0   3rd Qu.:11.000   3rd Qu.:12.000   3rd Qu.:17.00  
##  Max.   :119.0   Max.   :26.000   Max.   :20.000   Max.   :20.00
```

```r
data[, 2:20]          <- scale(data[, 2:20])
colnames(data)[2:20] <- paste0("x", 1:19)
```

F.2.1 Model specification

The syntax becomes more elaborate, due to the additional specification of the mean structure.

```
syntax.mg <-
'
# Measurement model
spatial =~ 1*x1  + x2 + x3 + x4 + x5 + x6 + x7 + x8 + 0*x9 + x10 +
             x11 + 0*x12 + x13 + 0*x14 + x15 + x16 + x17 + x18 + x19
verbal  =~ 0*x1  + x2 + x3 + x4 + x5 + x6 + x7 + x8 + 1*x9 + x10 +
             x11 + 0*x12 + x13 + 0*x14 + x15 + x16 + x17 + x18 + x19
speed   =~ 0*x1  + x2 + x3 + x4 + x5 + x6 + x7 + x8 + 0*x9 + x10 +
             x11 + 1*x12 + x13 + 0*x14 + x15 + x16 + x17 + x18 + x19
memory  =~ 0*x1  + x2 + x3 + x4 + x5 + x6 + x7 + x8 + 0*x9 + x10 +
             x11 + 0*x12 + x13 + 1*x14 + x15 + x16 + x17 + x18 + x19

# Estimate intercepts
x2 + x3 +  x4 + x5 + x6 + x7 + x8 + x10 + x11 +
                             x13 + x15 + x16 + x17 + x18 + x19 ~ 1

# Fixed intercepts
x1 + x9 + x12 + x14 ~ 0*1

# Structural model
spatial ~~ NA*spatial
verbal  ~~ NA*verbal
speed   ~~ NA*speed
memory  ~~ NA*memory

spatial ~ NA*1
verbal  ~ NA*1
speed   ~ NA*1
memory  ~ NA*1 '
```

The mean structure can be explicitly introduced by including "intercept formulas" in the model syntax. These expressions are constituted by the name of the variable, followed by the tilde operator "~", and the number 1. If the variable appearing in the formula is an observed variable, then the formula specifies the intercept term for that item; if the variable is latent (i.e., a common factor), then the formula specifies a factor mean. To avoid clutter, if users desire to introduce intercepts for multiple variables, they can specify on the left-hand side all the variables of interest, followed by plus ("+") signs. By default, the factor means are fixed to zero. Provided that identification restrictions are applied, users can force the estimation of any model parameter by pre-multiplying

the variable name on the right-hand side by NA. This is done in the syntax for the means and the variances of the common factors.

The syntax above specifies a factor model with $r = 4$ factors and $p = 19$ observed variables. The metric of the factors is accommodated through the "marker-variable" approach, with the markers being x_1, x_9, x_{12}, x_{14}. The structural model is freely estimated. The fact that the syntax should prompt a multiple-group analysis is communicated to penfa through proper arguments (see below for details). The model in the syntax is fitted to all groups.

Before carrying out the penalized estimation, we fit the unpenalized model to obtain the maximum likelihood estimates to be used as weights for the alasso. To facilitate the estimation process, we can provide informative starting values to (some of) the parameters. This can be done through the pre-multiplication mechanism employed to fix some parameter values, but the numeric constant becomes the argument of the function start. To fix parameters or provide starting values in case of multiple groups, we use the same pre-multiplication mechanism, but the numeric argument is a vector of arguments, one for each group. When users provide a single value instead of a vector of values, that element is applied for all groups. The syntax below provides a starting value equal to 0.8 to the primary loadings of all factors.

```
syntax.mle.mg <- '
# Measurement model + starting values
spatial =~  1*x1 + start(0.8)*x2 + start(0.8)*x3 + start(0.8)*x4 +
            x5 + x6 + x7 + x8 + 0*x9 + x10 + x11 + 0*x12 + x13 +
            0*x14 + x15 + x16 + x17 + x18 + x19

verbal  =~  0*x1 + x2 + x3 + x4 + start(0.8)*x5 + start(0.8)*x6 +
            start(0.8)*x7 + start(0.8)*x8 + 1*x9 + x10 + x11 +
            0*x12 + x13 + 0*x14 + x15 + x16 + x17 + x18 + x19

speed   =~  0*x1 + x2 + x3 + x4 + x5 + x6 + x7 + x8 + 0*x9 +
            start(0.8)*x10 + start(0.8)*x11 + 1*x12 +
            start(0.8)*x13 + 0*x14 + x15 + x16 + x17 + x18 + x19

memory  =~  0*x1 + x2 + x3 + x4 + x5 + x6 + x7 + x8 + 0*x9 + x10 +
            x11 + 0*x12 + x13 + 1*x14 + start(0.8)*x15 +
            start(0.8)*x16 + start(0.8)*x17 + start(0.8)*x18 +
            start(0.8)*x19

# Estimate intercepts
x2 + x3 +  x4 + x5 + x6 + x7 + x8 + x10 + x11 + x13 + x15 + x16 +
```

```
                                                 x17 + x18 + x19 ~ 1
# Fix intercepts
x1 + x9 + x12 + x14 ~ 0*1

# Structural model
spatial ~~ NA*spatial
verbal  ~~ NA*verbal
speed   ~~ NA*speed
memory  ~~ NA*memory

spatial ~ NA*1
verbal  ~ NA*1
speed   ~ NA*1
memory  ~ NA*1 '
```

As for the single-group analysis, the fit of the unpenalized multiple-group factor model is carried out through the `penfa` function, with the specification of two new arguments: `meanstructure` and `group`. The argument `meanstructure` is set to `TRUE` to obtain the estimates of the means of the observed and the latent variables. In the `group` argument, we indicate the name of the group variable in the data set, which is the "school" attended by the students.

```
fit.mle.mg <- penfa(# factor model
                    model = syntax.mle.mg,
                    data  = data,
                    information = "fisher",
                    meanstructure = TRUE,
                    group = "school",
                    # No penalization
                    pen.shrink = "none",
                    pen.diff = "none",
                    eta = list(shrink = c("none" = 0),
                               diff   = c("none" = 0)),
                    strategy = "fixed",
                    verbose = FALSE)
weights.mg <- coef(fit.mle.mg)
fit.mle.mg

## penfa reached convergence
##
##   Number of observations per group:
##     Pasteur                                                156
##     Grant-White                                            145
##
```

```
##   Estimator                                              MLE
##   Optimization method                          trust-region
##   Information                                        fisher
##   Strategy                                            fixed
##   Number of iterations                                   21
##   Effective degrees of freedom                      216.000
##
```

F.2.2 Model fitting

We can now proceed with the estimation of the penalized multiple-group factor model with the alasso penalization and the automatic tuning procedure to find the optimal value of the tuning parameter vector $\boldsymbol{\eta} = (\eta_1, \eta_2, \eta_3)^T$. The penalty function employed to shrink the pairwise group differences of the factor loadings and the intercepts can be specified through the `diff` argument. The argument `eta` is now a list that determines the starting values for each of the tuning parameters on the specified parameter matrices and vectors.

```
fit.mg <- penfa(# factor model
                model = syntax.mg,
                data  = data,
                information = "fisher",
                meanstructure = TRUE,
                group = "school",
                # penalization
                pen.shrink = "alasso",
                pen.diff = "alasso",
                eta = list(shrink = c("lambda" = 0.01),
                           diff   = c("lambda" = 0.1, "tau" = 0.01)),
                # automatic procedure
                strategy = "auto",
                gamma = 4,
                # alasso
                a.alasso = 1,
                weights = weights.mg,
                verbose = FALSE)
```

From the summary of the fitted object, we can notice that the automatic tuning procedure required just a couple of iterations to converge. The optimal tuning parameters are $\hat{\eta}_1 = 0.006$, $\hat{\eta}_2 = 16221.852$ and $\hat{\eta}_3 = 0.013$. The analysis benefited from the encouragement of sparsity and loading

and intercept invariance, as it is evident from the reduction in the GBIC after the penalization (from 15123.43 for the unpenalized model to 14658 for the penalized model).

```
summary(fit.mg)

## penfa reached convergence
##
##   Number of observations per group:
##     Pasteur                                                   156
##     Grant-White                                               145
##   Number of groups                                              2
##   Number of observed variables                                 19
##   Number of latent factors                                      4
##
##   Estimator                                                  PMLE
##   Optimization method                                trust-region
##   Information                                              fisher
##   Strategy                                                   auto
##   Number of iterations (total)                                347
##   Number of two-steps (automatic)                               5
##   Influence factor                                              4
##   Number of parameters:
##     Free                                                       66
##     Penalized                                                 150
##   Effective degrees of freedom                            109.242
##   GIC                                                   14253.027
##   GBIC                                                  14657.998
##
##   Penalty functions:
##     Sparsity                                               alasso
##     Invariance                                             alasso
##
##   Additional tuning parameter
##     alasso                                                      1
##
##   Optimal tuning parameters:
##     Sparsity
##      - Factor loadings                                      0.006
##     Invariance
##      - Factor loadings                                  16221.852
##      - Intercepts                                           0.013
##
##
## Parameter Estimates:
##
## Group 1 [Pasteur]:
##
## Latent Variables:
##                   Type    Estimate    Std.Err       2.5%      97.5%
##   spatial =~
```

```
##     x1            fixed      1.000                1.000       1.000
##     x2            pen        0.583      0.082     0.423       0.744
##     x3            pen        0.618      0.082     0.457       0.779
##     x4            pen        0.863      0.094     0.678       1.047
##     x5            pen       -0.000
##     x6            pen        0.000
##     x7            pen       -0.121      0.045    -0.210      -0.032
##     x8            pen        0.000
##     x9            fixed      0.000                0.000       0.000
##     x10           pen       -0.401      0.095    -0.588      -0.215
##     x11           pen        0.000
##     x12           fixed      0.000                0.000       0.000
##     x13           pen        0.397      0.078     0.245       0.550
##     x14           fixed      0.000                0.000       0.000
##     x15           pen        0.018
##     x16           pen        0.367      0.080     0.211       0.523
##     x17           pen       -0.231      0.077    -0.382      -0.080
##     x18           pen        0.001
##     x19           pen        0.059      0.042     0.024       0.142
##   verbal =~
##     x1            fixed      0.000                0.000       0.000
##     x2            pen       -0.000
##     x3            pen        0.000
##     x4            pen       -0.087      0.051    -0.187       0.013
##     x5            pen        1.020      0.056     0.910       1.130
##     x6            pen        0.957      0.055     0.849       1.064
##     x7            pen        1.075      0.059     0.960       1.191
##     x8            pen        0.839      0.058     0.725       0.952
##     x9            fixed      1.000                1.000       1.000
##     x10           pen        0.141      0.064     0.015       0.267
##     x11           pen        0.168      0.052     0.066       0.270
##     x12           fixed      0.000                0.000       0.000
##     x13           pen       -0.000
##     x14           fixed      0.000                0.000       0.000
##     x15           pen       -0.143      0.055    -0.250      -0.036
##     x16           pen       -0.000
##     x17           pen        0.000
##     x18           pen        0.000
##     x19           pen        0.000
##   speed =~
##     x1            fixed      0.000                0.000       0.000
##     x2            pen       -0.000
##     x3            pen        0.000
##     x4            pen       -0.000
##     x5            pen        0.000
##     x6            pen       -0.000
##     x7            pen       -0.000
##     x8            pen        0.000
##     x9            fixed      0.000                0.000       0.000
##     x10           pen        0.988      0.113     0.765       1.210
##     x11           pen        0.744      0.089     0.570       0.918
```

```
##     x12          fixed      1.000                 1.000      1.000
##     x13          pen        0.677      0.087      0.506      0.848
##     x14          fixed      0.000                 0.000      0.000
##     x15          pen        0.000
##     x16          pen        0.000
##     x17          pen        0.321      0.078      0.168      0.475
##     x18          pen        0.245      0.070      0.108      0.382
##     x19          pen        0.093      0.045      0.005      0.181
##   memory =~
##     x1           fixed      0.000                 0.000      0.000
##     x2           pen       -0.000
##     x3           pen       -0.000
##     x4           pen        0.000
##     x5           pen       -0.109      0.045     -0.198     -0.020
##     x6           pen        0.009
##     x7           pen       -0.000
##     x8           pen        0.028
##     x9           fixed      0.000                 0.000      0.000
##     x10          pen        0.145      0.073      0.002      0.288
##     x11          pen        0.267      0.079      0.113      0.422
##     x12          fixed      0.000                 0.000      0.000
##     x13          pen       -0.000
##     x14          fixed      1.000                 1.000      1.000
##     x15          pen        0.838      0.110      0.624      1.053
##     x16          pen        0.632      0.100      0.435      0.828
##     x17          pen        0.875      0.115      0.649      1.100
##     x18          pen        0.647      0.098      0.455      0.840
##     x19          pen        0.533      0.093      0.351      0.714
##
## Covariances:
##                  Type    Estimate    Std.Err       2.5%      97.5%
##   spatial ~~
##     verbal       free       0.281      0.067      0.150      0.411
##     speed        free       0.158      0.062      0.037      0.278
##     memory       free       0.174      0.064      0.049      0.300
##   verbal ~~
##     speed        free       0.185      0.059      0.071      0.300
##     memory       free       0.104      0.059     -0.012      0.220
##   speed ~~
##     memory       free       0.075      0.057     -0.038      0.187
##
##                  Type    Estimate    Std.Err       2.5%      97.5%
##    .x2           pen        0.009      0.056     -0.100      0.119
##    .x3           pen        0.001      0.056     -0.108      0.110
##    .x4           pen        0.137      0.070      0.001      0.273
##    .x5           pen       -0.012      0.044     -0.099      0.074
##    .x6           pen       -0.007      0.044     -0.094      0.079
##    .x7           pen       -0.006      0.043     -0.091      0.079
##    .x8           pen       -0.081      0.055     -0.188      0.026
##    .x10          pen        0.145      0.078     -0.008      0.298
##    .x11          pen        0.000      0.053     -0.104      0.104
```

```
##    .x13           pen       -0.002     0.052    -0.104     0.099
##    .x15           pen        0.000     0.060    -0.117     0.118
##    .x16           pen        0.016     0.054    -0.090     0.121
##    .x17           pen        0.164     0.077     0.012     0.316
##    .x18           pen       -0.002     0.057    -0.114     0.110
##    .x19           pen       -0.204     0.075    -0.352    -0.057
##    .x1            fixed      0.000               0.000     0.000
##    .x9            fixed      0.000               0.000     0.000
##    .x12           fixed      0.000               0.000     0.000
##    .x14           fixed      0.000               0.000     0.000
##     spatial       free      -0.021     0.077    -0.173     0.130
##     verbal        free      -0.259     0.073    -0.402    -0.116
##     speed         free       0.089     0.074    -0.055     0.234
##     memory        free      -0.046     0.077    -0.198     0.105
##
## Variances:
##                   Type    Estimate    Std.Err      2.5%     97.5%
##     spatial       free       0.591     0.106     0.384     0.798
##     verbal        free       0.656     0.091     0.477     0.834
##     speed         free       0.441     0.087     0.271     0.612
##     memory        free       0.519     0.104     0.315     0.722
##    .x1            free       0.437     0.079     0.283     0.591
##    .x2            free       0.886     0.107     0.677     1.095
##    .x3            free       0.814     0.099     0.619     1.008
##    .x4            free       0.612     0.087     0.442     0.781
##    .x5            free       0.257     0.038     0.183     0.331
##    .x6            free       0.348     0.046     0.258     0.439
##    .x7            free       0.254     0.039     0.179     0.330
##    .x8            free       0.407     0.051     0.307     0.506
##    .x9            free       0.230     0.035     0.162     0.298
##    .x10           free       0.523     0.085     0.356     0.689
##    .x11           free       0.441     0.061     0.321     0.561
##    .x12           free       0.543     0.084     0.378     0.707
##    .x13           free       0.617     0.082     0.456     0.778
##    .x14           free       0.580     0.091     0.402     0.758
##    .x15           free       0.676     0.092     0.495     0.857
##    .x16           free       0.735     0.094     0.550     0.919
##    .x17           free       0.625     0.089     0.450     0.800
##    .x18           free       0.778     0.096     0.589     0.966
##    .x19           free       0.846     0.101     0.648     1.044
##
## Group 2 [Grant-White]:
##
## Latent Variables:
##                   Type    Estimate    Std.Err      2.5%     97.5%
##   spatial =~
##     x1            fixed      1.000               1.000     1.000
##     x2            pen        0.583     0.082     0.423     0.744
##     x3            pen        0.618     0.082     0.457     0.779
##     x4            pen        0.863     0.094     0.678     1.047
##     x5            pen       -0.000
```

```
##     x6            pen        0.000
##     x7            pen       -0.121      0.045     -0.210     -0.032
##     x8            pen        0.000
##     x9            fixed      0.000                 0.000      0.000
##     x10           pen       -0.401      0.095     -0.588     -0.215
##     x11           pen        0.000
##     x12           fixed      0.000                 0.000      0.000
##     x13           pen        0.397      0.078      0.245      0.550
##     x14           fixed      0.000                 0.000      0.000
##     x15           pen        0.018
##     x16           pen        0.367      0.080      0.211      0.523
##     x17           pen       -0.231      0.077     -0.382     -0.080
##     x18           pen        0.001
##     x19           pen        0.059      0.042     -0.024      0.142
##   verbal =~
##     x1            fixed      0.000                 0.000      0.000
##     x2            pen       -0.000
##     x3            pen        0.000
##     x4            pen       -0.087      0.051     -0.187      0.013
##     x5            pen        1.020      0.056      0.910      1.130
##     x6            pen        0.957      0.055      0.849      1.064
##     x7            pen        1.075      0.059      0.960      1.191
##     x8            pen        0.839      0.058      0.725      0.952
##     x9            fixed      1.000                 1.000      1.000
##     x10           pen        0.141      0.064      0.015      0.267
##     x11           pen        0.168      0.052      0.066      0.270
##     x12           fixed      0.000                 0.000      0.000
##     x13           pen       -0.000
##     x14           fixed      0.000                 0.000      0.000
##     x15           pen       -0.143      0.055     -0.250     -0.036
##     x16           pen       -0.000
##     x17           pen        0.000
##     x18           pen        0.000
##     x19           pen        0.000
##   speed =~
##     x1            fixed      0.000                 0.000      0.000
##     x2            pen       -0.000
##     x3            pen        0.000
##     x4            pen       -0.000
##     x5            pen        0.000
##     x6            pen       -0.000
##     x7            pen       -0.000
##     x8            pen        0.000
##     x9            fixed      0.000                 0.000      0.000
##     x10           pen        0.988      0.113      0.765      1.210
##     x11           pen        0.744      0.089      0.570      0.918
##     x12           fixed      1.000                 1.000      1.000
##     x13           pen        0.677      0.087      0.506      0.848
##     x14           fixed      0.000                 0.000      0.000
##     x15           pen        0.000
##     x16           pen        0.000
```

```
##     x17           pen         0.321      0.078      0.168      0.475
##     x18           pen         0.245      0.070      0.108      0.382
##     x19           pen         0.093      0.045      0.005      0.181
##   memory =~
##     x1            fixed       0.000                 0.000      0.000
##     x2            pen        -0.000
##     x3            pen        -0.000
##     x4            pen         0.000
##     x5            pen        -0.109      0.045     -0.198     -0.020
##     x6            pen         0.009
##     x7            pen        -0.000
##     x8            pen         0.028
##     x9            fixed       0.000                 0.000      0.000
##     x10           pen         0.145      0.073      0.002      0.288
##     x11           pen         0.267      0.079      0.113      0.422
##     x12           fixed       0.000                 0.000      0.000
##     x13           pen        -0.000
##     x14           fixed       1.000                 1.000      1.000
##     x15           pen         0.838      0.110      0.624      1.053
##     x16           pen         0.632      0.100      0.435      0.828
##     x17           pen         0.875      0.115      0.649      1.100
##     x18           pen         0.647      0.098      0.455      0.840
##     x19           pen         0.533      0.093      0.351      0.714
##
## Covariances:
##                   Type    Estimate    Std.Err       2.5%      97.5%
##   spatial ~~
##     verbal        free        0.363      0.071      0.223      0.503
##     speed         free        0.289      0.074      0.143      0.434
##     memory        free        0.242      0.064      0.117      0.367
##   verbal ~~
##     speed         free        0.231      0.067      0.100      0.362
##     memory        free        0.257      0.061      0.138      0.375
##   speed ~~
##     memory        free        0.158      0.062      0.037      0.279
##
## Intercepts:
##                   Type    Estimate    Std.Err       2.5%      97.5%
##    .x2            pen         0.011      0.056     -0.098      0.121
##    .x3            pen         0.001      0.056     -0.108      0.110
##    .x4            pen        -0.163      0.067     -0.294     -0.032
##    .x5            pen        -0.008      0.044     -0.095      0.079
##    .x6            pen        -0.007      0.044     -0.094      0.079
##    .x7            pen        -0.006      0.043     -0.091      0.079
##    .x8            pen         0.074      0.059     -0.041      0.189
##    .x10           pen        -0.179      0.072     -0.319     -0.038
##    .x11           pen        -0.000      0.053     -0.104      0.104
##    .x13           pen        -0.002      0.052     -0.104      0.099
##    .x15           pen        -0.000      0.060     -0.118      0.117
##    .x16           pen         0.016      0.054     -0.089      0.121
##    .x17           pen        -0.191      0.073     -0.335     -0.048
```

```
##    .x18          pen       -0.002     0.057    -0.114     0.110
##    .x19          pen        0.235     0.068     0.102     0.369
##    .x1           fixed      0.000               0.000     0.000
##    .x9           fixed      0.000               0.000     0.000
##    .x12          fixed      0.000               0.000     0.000
##    .x14          fixed      0.000               0.000     0.000
##     spatial      free       0.023     0.080    -0.134     0.180
##     verbal       free       0.289     0.075     0.141     0.436
##     speed        free      -0.085     0.082    -0.246     0.075
##     memory       free       0.052     0.075    -0.095     0.199
##
## Variances:
##                 Type    Estimate   Std.Err      2.5%     97.5%
##     spatial      free       0.597     0.108     0.385     0.808
##     verbal       free       0.625     0.092     0.445     0.805
##     speed        free       0.627     0.115     0.402     0.851
##     memory       free       0.420     0.088     0.248     0.591
##    .x1           free       0.435     0.074     0.290     0.579
##    .x2           free       0.683     0.086     0.515     0.851
##    .x3           free       0.712     0.090     0.536     0.888
##    .x4           free       0.472     0.070     0.334     0.610
##    .x5           free       0.311     0.045     0.222     0.400
##    .x6           free       0.313     0.044     0.226     0.400
##    .x7           free       0.219     0.037     0.147     0.292
##    .x8           free       0.446     0.058     0.333     0.560
##    .x9           free       0.346     0.049     0.250     0.442
##    .x10          free       0.335     0.067     0.203     0.467
##    .x11          free       0.615     0.082     0.454     0.775
##    .x12          free       0.442     0.075     0.295     0.590
##    .x13          free       0.443     0.065     0.315     0.570
##    .x14          free       0.556     0.084     0.392     0.719
##    .x15          free       0.674     0.091     0.496     0.851
##    .x16          free       0.471     0.065     0.344     0.598
##    .x17          free       0.464     0.069     0.328     0.601
##    .x18          free       0.649     0.083     0.487     0.812
##    .x19          free       0.600     0.075     0.453     0.746
```

The diagonal elements of the penalty matrix $\boldsymbol{\mathcal{S}}^{A}_{\hat{\eta}}(\hat{\boldsymbol{\theta}})$ are roughly in the range $[-3\times10^{12}, 3\times10^{12}]$. In Figure F.4a, we find the heat map of the penalty matrix $\boldsymbol{\mathcal{D}}^{A}_{\hat{\eta}_1}(\hat{\boldsymbol{\theta}})$, which shrinks the small factor loadings of each group to zero. Because the range of the diagonal elements of the penalty matrix is very wide, we employed the log-scale. The non-zero diagonal elements correspond to the factor loadings of the two groups. All of the remaining entries of the penalty matrix are equal to zero. Figure F.4b represents the heat map of the penalty matrix $\boldsymbol{\mathcal{D}}^{A}_{\hat{\eta}_2}(\hat{\boldsymbol{\theta}})$, which shrinks the pairwise group differences of the factor loadings towards zero. Similarly, the heat map of the penalty matrix $\boldsymbol{\mathcal{D}}^{A}_{\hat{\eta}_3}(\hat{\boldsymbol{\theta}})$ shrinks the pairwise group differences of the intercepts, and is depicted in Figure F.4c.

Further details, examples, and options can be found in the documentation of the `R` package `penfa`.

R session info: `mgcv` (version 1.8-24), `GJRM` (version 0.2).

(a) *Heat map of* $\mathcal{D}^{A}_{\hat{\eta}_1}(\hat{\boldsymbol{\theta}})$ *on a log-scale.*

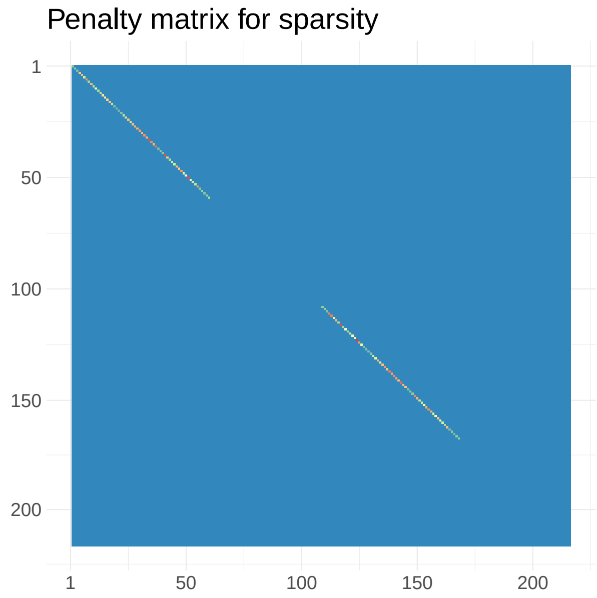

(b) *Heat map of* $\mathcal{D}^{A}_{\hat{\eta}_2}(\hat{\boldsymbol{\theta}})$ *on a log-scale.*

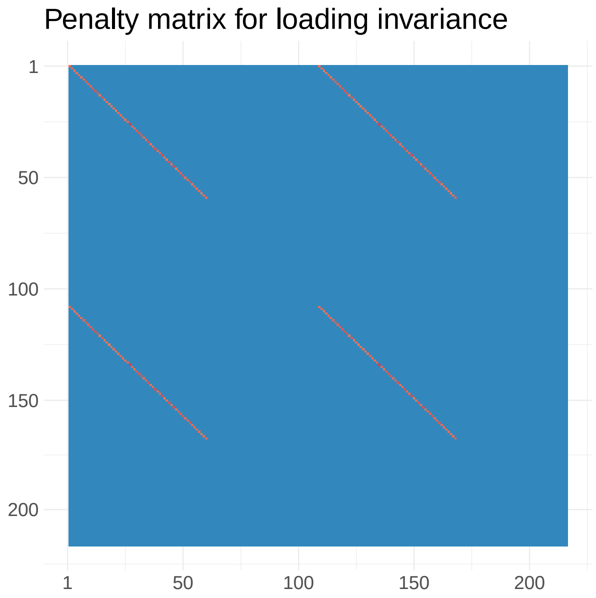

(c) *Heat map of* $\mathcal{D}^{A}_{\hat{\eta}_3}(\hat{\boldsymbol{\theta}})$ *on a log-scale.*

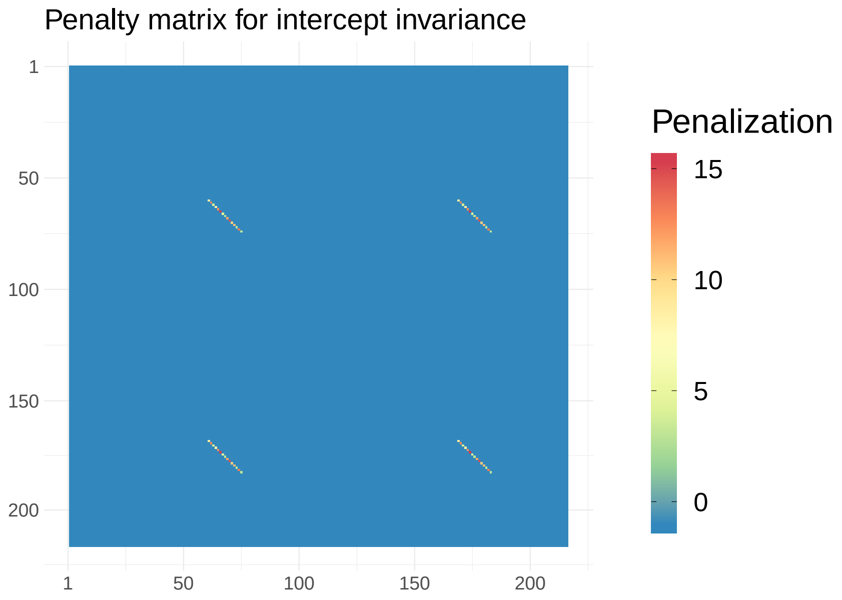

Figure F.4: Representation of the penalty matrices for sparsity of the factor loadings and loading and intercept invariance on a log-scale for `penfa-alasso` ($a = 1, \gamma = 4$) on the Holzinger & Swineford data set.

References

Akaike, H. (1974). A new look at the statistical model identification. *IEEE Transactions on Automatic Control*, *19*(6), 716–723.

Danaher, P., Wang, P. & Witten, D. M. (2014). The joint graphical lasso for inverse covariance estimation across multiple classes. *Journal of the Royal Statistical Society: Series B (Statistical Methodology)*, *76*(2), 373–397.

Fan, J. & Li, R. (2001). Variable selection via nonconcave penalized likelihood and its oracle properties. *Journal of the American Statistical Association*, *96*(456), 1348–1360.

Holzinger, K. J. & Swineford, F. (1939). *A study in factor analysis: The stability of a bi-factor solution.* Supplementary Educational Monographs, 48, University of Chicago.

Huang, P. (2018). A penalized likelihood method for multi-group structural equation modelling. *British Journal of Mathematical and Statistical Psychology*, *71*(3), 499–522.

Huang, P., Chen, H. & Weng, L. (2017). A penalized likelihood method for structural equation modeling. *Psychometrika*, *82*(2), 329–354.

Kelley, K. (2019). MBESS: The MBESS R package [Computer software manual]. Retrieved from https://CRAN.R-project.org/package=MBESS

Koch, I. (1996). On the asymptotic performance of median smoothers in image analysis and nonparametric regression. *The Annals of Statistics*, *24*(4), 1648–1666.

Konishi, S. & Kitagawa, G. (1996). Generalised information criteria in model selection. *Biometrika*, *83*(4), 875–890.

Konishi, S. & Kitagawa, G. (2008). *Information criteria and statistical modeling*. Springer Science & Business Media.

Marra, G. & Wood, S. N. (2012). Coverage properties of confidence intervals for generalized additive model components. *Scandinavian Journal of Statistics*, *39*(1), 53–74.

Petry, S. (2011). *Regularization approaches for generalized linear models and single index models* (Doctoral dissertation, Ludwig–Maximilians–Universität München). Retrieved from https://edoc.ub.uni-muenchen.de/14398/

Rosseel, Y. (2012). lavaan: An R package for structural equation modeling and more. *Journal of*

Statistical Software, *48*(2), 1–36.

Rosseel, Y. et al. (2019). lavaan: Latent Variable Analysis [Computer software manual]. Retrieved from https://CRAN.R-project.org/package=lavaan/ (R package version 0.6-5)

Schwarz, G. (1978). Estimating the dimension of a model. *The Annals of Statistics*, *6*(2), 461–464.

Ulbricht, J. (2010). *Variable selection in generalized linear models*. (Doctoral dissertation, Ludwig–Maximilians–Universität München). Verlag Dr. Hut.

Wood, S. N. (2017). *Generalized additive models: An introduction with R*. Chapman and Hall/CRC.

Yuan, K. & Bentler, P. M. (2006). Structural equation modeling. In C. Rao & S. Sinharay (Eds.), *Handbook of Statistics* (Vol. 10, pp. 297–358). Elsevier.

Zou, H. (2006). The adaptive lasso and its oracle properties. *Journal of the American Statistical Association*, *101*(476), 1418–1429.
